# Whole-genome sequencing reveals host factors underlying critical COVID-19

**DOI:** 10.1038/s41586-022-04576-6

**Published:** 2022-03-07

**Authors:** Athanasios Kousathanas, Erola Pairo-Castineira, Konrad Rawlik, Alex Stuckey, Christopher A. Odhams, Susan Walker, Clark D. Russell, Tomas Malinauskas, Yang Wu, Jonathan Millar, Xia Shen, Katherine S. Elliott, Fiona Griffiths, Wilna Oosthuyzen, Kirstie Morrice, Sean Keating, Bo Wang, Daniel Rhodes, Lucija Klaric, Marie Zechner, Nick Parkinson, Afshan Siddiq, Peter Goddard, Sally Donovan, David Maslove, Alistair Nichol, Malcolm G. Semple, Tala Zainy, Fiona Maleady-Crowe, Linda Todd, Shahla Salehi, Julian Knight, Greg Elgar, Georgia Chan, Prabhu Arumugam, Christine Patch, Augusto Rendon, David Bentley, Clare Kingsley, Jack A. Kosmicki, Julie E. Horowitz, Aris Baras, Goncalo R. Abecasis, Manuel A. R. Ferreira, Anne Justice, Tooraj Mirshahi, Matthew Oetjens, Daniel J. Rader, Marylyn D. Ritchie, Anurag Verma, Tom A. Fowler, Manu Shankar-Hari, Charlotte Summers, Charles Hinds, Peter Horby, Lowell Ling, Danny McAuley, Hugh Montgomery, Peter J. M. Openshaw, Paul Elliott, Timothy Walsh, Albert Tenesa, GenOMICC investigators, J. Kenneth Baillie, Colin Begg, Sara Clohisey Hendry, Charles Hinds, Peter Horby, Julian Knight, Lowell Ling, David Maslove, Danny McAuley, Johnny Millar, Hugh Montgomery, Alistair Nichol, Peter J. M. Openshaw, Alexandre C. Pereira, Chris P. Ponting, Kathy Rowan, Malcolm G. Semple, Manu Shankar-Hari, Charlotte Summers, Timothy Walsh, Latha Aravindan, Ruth Armstrong, Heather Biggs, Ceilia Boz, Adam Brown, Richard Clark, Audrey Coutts, Judy Coyle, Louise Cullum, Sukamal Das, Nicky Day, Lorna Donnelly, Esther Duncan, Angie Fawkes, Paul Finernan, Max Head Fourman, Anita Furlong, James Furniss, Bernadette Gallagher, Tammy Gilchrist, Ailsa Golightly, Fiona Griffiths, Katarzyna Hafezi, Debbie Hamilton, Ross Hendry, Andy Law, Dawn Law, Rachel Law, Sarah Law, Rebecca Lidstone-Scott, Louise Macgillivray, Alan Maclean, Hanning Mal, Sarah McCafferty, Ellie Mcmaster, Jen Meikle, Shona C. Moore, Kirstie Morrice, Lee Murphy, Sheena Murphy, Mybaya Hellen, Wilna Oosthuyzen, Chenqing Zheng, Jiantao Chen, Nick Parkinson, Trevor Paterson, Katherine Schon, Andrew Stenhouse, Mihaela Das, Maaike Swets, Helen Szoor-McElhinney, Filip Taneski, Lance Turtle, Tony Wackett, Mairi Ward, Jane Weaver, Nicola Wrobel, Marie Zechner, Gill Arbane, Aneta Bociek, Sara Campos, Neus Grau, Tim Owen Jones, Rosario Lim, Martina Marotti, Marlies Ostermann, Manu Shankar-Hari, Christopher Whitton, Zoe Alldis, Raine Astin-Chamberlain, Fatima Bibi, Jack Biddle, Sarah Blow, Matthew Bolton, Catherine Borra, Ruth Bowles, Maudrian Burton, Yasmin Choudhury, David Collier, Amber Cox, Amy Easthope, Patrizia Ebano, Stavros Fotiadis, Jana Gurasashvili, Rosslyn Halls, Pippa Hartridge, Delordson Kallon, Jamila Kassam, Ivone Lancoma-Malcolm, Maninderpal Matharu, Peter May, Oliver Mitchelmore, Tabitha Newman, Mital Patel, Jane Pheby, Irene Pinzuti, Zoe Prime, Oleksandra Prysyazhna, Julian Shiel, Melanie Taylor, Carey Tierney, Suzanne Wood, Anne Zak, Olivier Zongo, Stephen Bonner, Keith Hugill, Jessica Jones, Steven Liggett, Evie Headlam, Nageswar Bandla, Minnie Gellamucho, Michelle Davies, Christopher Thompson, Marwa Abdelrazik, Dhanalakshmi Bakthavatsalam, Munzir Elhassan, Arunkumar Ganesan, Anne Haldeos, Jeronimo Moreno-Cuesta, Dharam Purohit, Rachel Vincent, Kugan Xavier, Rohit Kumar, Alasdair Frater, Malik Saleem, David Carter, Samuel Jenkins, Zoe Lamond, Alanna Wall, Jaime Fernandez-Roman, David O. Hamilton, Emily Johnson, Brian Johnston, Maria Lopez Martinez, Suleman Mulla, David Shaw, Alicia A. C. Waite, Victoria Waugh, Ingeborg D. Welters, Karen Williams, Anna Cavazza, Maeve Cockrell, Eleanor Corcoran, Maria Depante, Clare Finney, Ellen Jerome, Mark McPhail, Monalisa Nayak, Harriet Noble, Kevin O’Reilly, Evita Pappa, Rohit Saha, Sian Saha, John Smith, Abigail Knighton, David Antcliffe, Dorota Banach, Stephen Brett, Phoebe Coghlan, Ziortza Fernandez, Anthony Gordon, Roceld Rojo, Sonia Sousa Arias, Maie Templeton, Megan Meredith, Lucy Morris, Lucy Ryan, Amy Clark, Julia Sampson, Cecilia Peters, Martin Dent, Margaret Langley, Saima Ashraf, Shuying Wei, Angela Andrew, Archana Bashyal, Neil Davidson, Paula Hutton, Stuart McKechnie, Jean Wilson, David Baptista, Rebecca Crowe, Rita Fernandes, Rosaleen Herdman-Grant, Anna Joseph, Denise O’Connor, Meryem Allen, Adam Loveridge, India McKenley, Eriko Morino, Andres Naranjo, Richard Simms, Kathryn Sollesta, Andrew Swain, Harish Venkatesh, Jacyntha Khera, Jonathan Fox, Gillian Andrew, J. Kenneth Baillie, Lucy Barclay, Marie Callaghan, Rachael Campbell, Sarah Clark, Dave Hope, Lucy Marshall, Corrienne McCulloch, Kate Briton, Jo Singleton, Sophie Birch, Lutece Brimfield, Zoe Daly, David Pogson, Steve Rose, Angela Nown, Ceri Battle, Elaine Brinkworth, Rachel Harford, Carl Murphy, Luke Newey, Tabitha Rees, Marie Williams, Sophie Arnold, Petra Polgarova, Katerina Stroud, Charlotte Summers, Eoghan Meaney, Megan Jones, Anthony Ng, Shruti Agrawal, Nazima Pathan, Deborah White, Esther Daubney, Kay Elston, Lina Grauslyte, Musarat Hussain, Mandeep Phull, Tatiana Pogreban, Lace Rosaroso, Erika Salciute, George Franke, Joanna Wong, Aparna George, Laura Ortiz-Ruiz de Gordoa, Emily Peasgood, Claire Phillips, Michelle Bates, Jo Dasgin, Jaspret Gill, Annette Nilsson, James Scriven, Amy Collins, Waqas Khaliq, Estefania Treus Gude, Carlos Castro Delgado, Deborah Dawson, Lijun Ding, Georgia Durrant, Obiageri Ezeobu, Sarah Farnell-Ward, Abiola Harrison, Rebecca Kanu, Susannah Leaver, Elena Maccacari, Soumendu Manna, Romina Pepermans Saluzzio, Joana Queiroz, Tinashe Samakomva, Christine Sicat, Joana Texeira, Edna Fernandes Da Gloria, Ana Lisboa, John Rawlins, Jisha Mathew, Ashley Kinch, William James Hurt, Nirav Shah, Victoria Clark, Maria Thanasi, Nikki Yun, Kamal Patel, Sara Bennett, Emma Goodwin, Matthew Jackson, Alissa Kent, Clare Tibke, Wiesia Woodyatt, Ahmed Zaki, Azmerelda Abraheem, Peter Bamford, Kathryn Cawley, Charlie Dunmore, Maria Faulkner, Rumanah Girach, Helen Jeffrey, Rhianna Jones, Emily London, Imrun Nagra, Farah Nasir, Hannah Sainsbury, Clare Smedley, Tahera Patel, Matthew Smith, Srikanth Chukkambotla, Aayesha Kazi, Janice Hartley, Joseph Dykes, Muhammad Hijazi, Sarah Keith, Meherunnisa Khan, Janet Ryan-Smith, Philippa Springle, Jacqueline Thomas, Nick Truman, Samuel Saad, Dabheoc Coleman, Christopher Fine, Roseanna Matt, Bethan Gay, Jack Dalziel, Syamlan Ali, Drew Goodchild, Rhiannan Harling, Ravi Bhatterjee, Wendy Goddard, Chloe Davison, Stephen Duberly, Jeanette Hargreaves, Rachel Bolton, Miriam Davey, David Golden, Rebecca Seaman, Shiney Cherian, Sean Cutler, Anne Emma Heron, Anna Roynon-Reed, Tamas Szakmany, Gemma Williams, Owen Richards, Yusuf Cheema, Hollie Brooke, Sarah Buckley, Jose Cebrian Suarez, Ruth Charlesworth, Karen Hansson, John Norris, Alice Poole, Alastair Rose, Rajdeep Sandhu, Brendan Sloan, Elizabeth Smithson, Muthu Thirumaran, Veronica Wagstaff, Alexandra Metcalfe, Mark Brunton, Jess Caterson, Holly Coles, Matthew Frise, Sabi Gurung Rai, Nicola Jacques, Liza Keating, Emma Tilney, Shauna Bartley, Parminder Bhuie, Sian Gibson, Amanda Lyle, Fiona McNeela, Jayachandran Radhakrishnan, Alistair Hughes, Bryan Yates, Jessica Reynolds, Helen Campbell, Maria Thompsom, Steve Dodds, Stacey Duffy, Sandra Greer, Karen Shuker, Ascanio Tridente, Reena Khade, Ashok Sundar, George Tsinaslanidis, Isobel Birkinshaw, Joseph Carter, Kate Howard, Joanne Ingham, Rosie Joy, Harriet Pearson, Samantha Roche, Zoe Scott, Hollie Bancroft, Mary Bellamy, Margaret Carmody, Jacqueline Daglish, Faye Moore, Joanne Rhodes, Mirriam Sangombe, Salma Kadiri, James Scriven, Maria Croft, Ian White, Victoria Frost, Maia Aquino, Rajeev Jha, Vinodh Krishnamurthy, Lai Lim, Li Lim, Edward Combes, Teishel Joefield, Sonja Monnery, Valerie Beech, Sallyanne Trotman, Christine Almaden-Boyle, Pauline Austin, Louise Cabrelli, Stephen Cole, Matt Casey, Susan Chapman, Clare Whyte, Yolanda Baird, Aaron Butler, Indra Chadbourn, Linda Folkes, Heather Fox, Amy Gardner, Raquel Gomez, Gillian Hobden, Luke Hodgson, Kirsten King, Michael Margarson, Tim Martindale, Emma Meadows, Dana Raynard, Yvette Thirlwall, David Helm, Jordi Margalef, Kristine Criste, Rebecca Cusack, Kim Golder, Hannah Golding, Oliver Jones, Samantha Leggett, Michelle Male, Martyna Marani, Kirsty Prager, Toran Williams, Belinda Roberts, Karen Salmon, Peter Anderson, Katie Archer, Karen Austin, Caroline Davis, Alison Durie, Olivia Kelsall, Jessica Thrush, Charlie Vigurs, Laura Wild, Hannah-Louise Wood, Helen Tranter, Alison Harrison, Nicholas Cowley, Michael McAlindon, Andrew Burtenshaw, Stephen Digby, Emma Low, Aled Morgan, Naiara Cother, Tobias Rankin, Sarah Clayton, Alex McCurdy, Cecilia Ahmed, Balvinder Baines, Sarah Clamp, Julie Colley, Risna Haq, Anne Hayes, Jonathan Hulme, Samia Hussain, Sibet Joseph, Rita Kumar, Zahira Maqsood, Manjit Purewal, Leonie Benham, Zena Bradshaw, Joanna Brown, Melanie Caswell, Jason Cupitt, Sarah Melling, Stephen Preston, Nicola Slawson, Emma Stoddard, Scott Warden, Bethan Deacon, Ceri Lynch, Carla Pothecary, Lisa Roche, Gwenllian Sera Howe, Jayaprakash Singh, Keri Turner, Hannah Ellis, Natalie Stroud, Jodie Hunt, Joy Dearden, Emma Dobson, Andy Drummond, Michelle Mulcahy, Sheila Munt, Grainne O’Connor, Jennifer Philbin, Chloe Rishton, Redmond Tully, Sarah Winnard, Susanne Cathcart, Katharine Duffy, Alex Puxty, Kathryn Puxty, Lynne Turner, Jane Ireland, Gary Semple, Kate Long, Simon Whiteley, Elizabeth Wilby, Bethan Ogg, Amanda Cowton, Andrea Kay, Melanie Kent, Kathryn Potts, Ami Wilkinson, Suzanne Campbell, Ellen Brown, Julie Melville, Jay Naisbitt, Rosane Joseph, Maria Lazo, Olivia Walton, Alan Neal, Peter Alexander, Schvearn Allen, Joanne Bradley-Potts, Craig Brantwood, Jasmine Egan, Timothy Felton, Grace Padden, Luke Ward, Stuart Moss, Susannah Glasgow, Lynn Abel, Michael Brett, Brian Digby, Lisa Gemmell, James Hornsby, Patrick MacGoey, Pauline O’Neil, Richard Price, Natalie Rodden, Kevin Rooney, Radha Sundaram, Nicola Thomson, Bridget Hopkins, James Scriven, Laura Thrasyvoulou, Heather Willis, Martyn Clark, Martina Coulding, Edward Jude, Jacqueline McCormick, Oliver Mercer, Darsh Potla, Hafiz Rehman, Heather Savill, Victoria Turner, Charlotte Downes, Kathleen Holding, Katie Riches, Mary Hilton, Mel Hayman, Deepak Subramanian, Priya Daniel, Oluronke Adanini, Nikhil Bhatia, Maines Msiska, Rebecca Collins, Ian Clement, Bijal Patel, A. Gulati, Carole Hays, K. Webster, Anne Hudson, Andrea Webster, Elaine Stephenson, Louise McCormack, Victoria Slater, Rachel Nixon, Helen Hanson, Maggie Fearby, Sinead Kelly, Victoria Bridgett, Philip Robinson, Julie Camsooksai, Charlotte Humphrey, Sarah Jenkins, Henrik Reschreiter, Beverley Wadams, Yasmin Death, Victoria Bastion, Daphene Clarke, Beena David, Harriet Kent, Rachel Lorusso, Gamu Lubimbi, Sophie Murdoch, Melchizedek Penacerrada, Alastair Thomas, Jennifer Valentine, Ana Vochin, Retno Wulandari, Brice Djeugam, Gillian Bell, Katy English, Amro Katary, Louise Wilcox, Michelle Bruce, Karen Connolly, Tracy Duncan, Helen T. Michael, Gabriella Lindergard, Samuel Hey, Claire Fox, Jordan Alfonso, Laura Jayne Durrans, Jacinta Guerin, Bethan Blackledge, Jade Harris, Martin Hruska, Ayaa Eltayeb, Thomas Lamb, Tracey Hodgkiss, Lisa Cooper, Joanne Rothwell, Angela Allan, Felicity Anderson, Callum Kaye, Jade Liew, Jasmine Medhora, Teresa Scott, Erin Trumper, Adriana Botello, Liana Lankester, Nikitas Nikitas, Colin Wells, Bethan Stowe, Kayleigh Spencer, Craig Brandwood, Lara Smith, Richard Clark, Katie Birchall, Laurel Kolakaluri, Deborah Baines, Anila Sukumaran, Elena Apetri, Cathrine Basikolo, Bethan Blackledge, Laura Catlow, Bethan Charles, Paul Dark, Reece Doonan, Jade Harris, Alice Harvey, Daniel Horner, Karen Knowles, Stephanie Lee, Diane Lomas, Chloe Lyons, Tracy Marsden, Danielle McLaughlan, Liam McMorrow, Jessica Pendlebury, Jane Perez, Maria Poulaka, Nicola Proudfoot, Melanie Slaughter, Kathryn Slevin, Melanie Taylor, Vicky Thomas, Danielle Walker, Angiy Michael, Matthew Collis, Tracey Cosier, Gemma Millen, Neil Richardson, Natasha Schumacher, Heather Weston, James Rand, Nicola Baxter, Steven Henderson, Sophie Kennedy-Hay, Christopher McParland, Laura Rooney, Malcolm Sim, Gordan McCreath, Louise Akeroyd, Shereen Bano, Matt Bromley, Lucy Gurr, Tom Lawton, James Morgan, Kirsten Sellick, Deborah Warren, Brian Wilkinson, Janet McGowan, Camilla Ledgard, Amelia Stacey, Kate Pye, Ruth Bellwood, Michael Bentley, Jeremy Bewley, Zoe Garland, Lisa Grimmer, Bethany Gumbrill, Rebekah Johnson, Katie Sweet, Denise Webster, Georgia Efford, Karen Convery, Deirdre Fottrell-Gould, Lisa Hudig, Jocelyn Keshet-Price, Georgina Randell, Katie Stammers, Maria Bokhari, Vanessa Linnett, Rachael Lucas, Wendy McCormick, Jenny Ritzema, Amanda Sanderson, Helen Wild, Anthony Rostron, Alistair Roy, Lindsey Woods, Sarah Cornell, Fiona Wakinshaw, Kimberley Rogerson, Jordan Jarmain, Robert Parker, Amie Reddy, Ian Turner-Bone, Laura Wilding, Peter Harding, Caroline Abernathy, Louise Foster, Andrew Gratrix, Vicky Martinson, Priyai Parkinson, Elizabeth Stones, Llucia Carbral-Ortega, Georgia Bercades, David Brealey, Ingrid Hass, Niall MacCallum, Gladys Martir, Eamon Raith, Anna Reyes, Deborah Smyth, Letizia Zitter, Sarah Benyon, Suzie Marriott, Linda Park, Samantha Keenan, Elizabeth Gordon, Helen Quinn, Kizzy Baines, Lenka Cagova, Adama Fofano, Lucie Garner, Helen Holcombe, Sue Mepham, Alice Michael Mitchell, Lucy Mwaura, Krithivasan Praman, Alain Vuylsteke, Julie Zamikula, Bally Purewal, Vanessa Rivers, Stephanie Bell, Hayley Blakemore, Borislava Borislavova, Beverley Faulkner, Emma Gendall, Elizabeth Goff, Kati Hayes, Matt Thomas, Ruth Worner, Kerry Smith, Deanna Stephens, Louise Mew, Esther Mwaura, Richard Stewart, Felicity Williams, Lynn Wren, Sara-Beth Sutherland, Emily Bevan, Jane Martin, Dawn Trodd, Geoff Watson, Caroline Wrey Brown, Olugbenga Akinkugbe, Alasdair Bamford, Emily Beech, Holly Belfield, Michael Bell, Charlene Davies, Gareth A. L. Jones, Tara McHugh, Hamza Meghari, Lauran O’Neill, Mark J. Peters, Samiran Ray, Ana Luisa Tomas, Iona Burn, Geraldine Hambrook, Katarina Manso, Ruth Penn, Pradeep Shanmugasundaram, Julie Tebbutt, Danielle Thornton, Jade Cole, Michelle Davies, Rhys Davies, Donna Duffin, Helen Hill, Ben Player, Emma Thomas, Angharad Williams, Denise Griffin, Nycola Muchenje, Mcdonald Mupudzi, Richard Partridge, Jo-Anna Conyngham, Rachel Thomas, Mary Wright, Maria Alvarez Corral, Reni Jacob, Cathy Jones, Craig Denmade, Sarah Beavis, Katie Dale, Rachel Gascoyne, Joanne Hawes, Kelly Pritchard, Lesley Stevenson, Amanda Whileman, Patricia Doble, Joanne Hutter, Corinne Pawley, Charmaine Shovelton, Marius Vaida, Deborah Butcher, Susie O’Sullivan, Nicola Butterworth-Cowin, Norfaizan Ahmad, Joann Barker, Kris Bauchmuller, Sarah Bird, Kay Cawthron, Kate Harrington, Yvonne Jackson, Faith Kibutu, Becky Lenagh, Shamiso Masuko, Gary H. Mills, Ajay Raithatha, Matthew Wiles, Jayne Willson, Helen Newell, Alison Lye, Lorenza Nwafor, Claire Jarman, Sarah Rowland-Jones, David Foote, Joby Cole, Roger Thompson, James Watson, Lisa Hesseldon, Irene Macharia, Luke Chetam, Jacqui Smith, Amber Ford, Samantha Anderson, Kathryn Birchall, Kay Housley, Sara Walker, Leanne Milner, Helena Hanratty, Helen Trower, Patrick Phillips, Simon Oxspring, Ben Donne, Catherine Jardine, Dewi Williams, Alasdair Hay, Rebecca Flanagan, Gareth Hughes, Scott Latham, Emma McKenna, Jennifer Anderson, Robert Hull, Kat Rhead, Carina Cruz, Natalie Pattison, Rob Charnock, Denise McFarland, Denise Cosgrove, Ashar Ahmed, Anna Morris, Srinivas Jakkula, Arvind Nune, Asifa Ali, Megan Brady, Sam Dale, Annalisa Dance, Lisa Gledhill, Jill Greig, Kathryn Hanson, Kelly Holdroyd, Marie Home, Diane Kelly, Ross Kitson, Lear Matapure, Deborah Melia, Samantha Mellor, Tonicha Nortcliffe, Jez Pinnell, Matthew Robinson, Lisa Shaw, Ryan Shaw, Lesley Thomis, Alison Wilson, Tracy Wood, Lee-Ann Bayo, Ekta Merwaha, Tahira Ishaq, Sarah Hanley, Bethan Deacon, Meg Hibbert, Carla Pothecary, Dariusz Tetla, Christopher Woodford, Latha Durga, Gareth Kennard-Holden, Debbie Branney, Jordan Frankham, Sally Pitts, Nigel White, Shondipon Laha, Mark Verlander, Alexandra Williams, Abdelhakim Altabaibeh, Ana Alvaro, Kayleigh Gilbert, Louise Ma, Loreta Mostoles, Chetan Parmar, Kathryn Simpson, Champa Jetha, Lauren Booker, Anezka Pratley, Colene Adams, Anita Agasou, Tracie Arden, Amy Bowes, Pauline Boyle, Mandy Beekes, Heather Button, Nigel Capps, Mandy Carnahan, Anne Carter, Danielle Childs, Denise Donaldson, Kelly Hard, Fran Hurford, Yasmin Hussain, Ayesha Javaid, James Jones, Sanal Jose, Michael Leigh, Terry Martin, Helen Millward, Nichola Motherwell, Rachel Rikunenko, Jo Stickley, Julie Summers, Louise Ting, Helen Tivenan, Louise Tonks, Rebecca Wilcox, Denise Skinner, Jane Gaylard, Dee Mullan, Julie Newman, Maureen Holland, Natalie Keenan, Marc Lyons, Helen Wassall, Chris Marsh, Mervin Mahenthran, Emma Carter, Thomas Kong, Helen Blackman, Ben Creagh-Brown, Sinead Donlon, Natalia Michalak-Glinska, Sheila Mtuwa, Veronika Pristopan, Armorel Salberg, Eleanor Smith, Sarah Stone, Charles Piercy, Jerik Verula, Dorota Burda, Rugia Montaser, Lesley Harden, Irving Mayangao, Cheryl Marriott, Paul Bradley, Celia Harris, Susan Anderson, Eleanor Andrews, Janine Birch, Emma Collins, Kate Hammerton, Ryan O’Leary, Michele Clark, Sarah Purvis, Russell Barber, Claire Hewitt, Annette Hilldrith, Karen Jackson-Lawrence, Sarah Shepardson, Maryanne Wills, Susan Butler, Silvia Tavares, Amy Cunningham, Julia Hindale, Sarwat Arif, Sarah Bean, Karen Burt, Michael Spivey, Carrie Demetriou, Charlotte Eckbad, Sarah Hierons, Lucy Howie, Sarah Mitchard, Lidia Ramos, Alfredo Serrano-Ruiz, Katie White, Fiona Kelly, Daniele Cristiano, Natalie Dormand, Zohreh Farzad, Mahitha Gummadi, Kamal Liyanage, Brijesh Patel, Sara Salmi, Geraldine Sloane, Vicky Thwaites, Mathew Varghese, Anelise C. Zborowski, John Allan, Tim Geary, Gordon Houston, Alistair Meikle, Peter O’Brien, Miranda Forsey, Agilan Kaliappan, Anne Nicholson, Joanne Riches, Mark Vertue, Elizabeth Allan, Kate Darlington, Ffyon Davies, Jack Easton, Sumit Kumar, Richard Lean, Daniel Menzies, Richard Pugh, Xinyi Qiu, Llinos Davies, Hannah Williams, Jeremy Scanlon, Gwyneth Davies, Callum Mackay, Joanne Lewis, Stephanie Rees, Metod Oblak, Monica Popescu, Mini Thankachen, Andrew Higham, Kerry Simpson, Jayne Craig, Rosie Baruah, Sheila Morris, Susie Ferguson, Amy Shepherd, Luke Stephen Prockter Moore, Marcela Paola Vizcaychipi, Laura Gomes de Almeida Martins, Jaime Carungcong, Inthakab Ali Mohamed Ali, Karen Beaumont, Mark Blunt, Zoe Coton, Hollie Curgenven, Mohamed Elsaadany, Kay Fernandes, Sameena Mohamed Ally, Harini Rangarajan, Varun Sarathy, Sivarupan Selvanayagam, Dave Vedage, Matthew White, Mandy Gill, Paul Paul, Valli Ratnam, Sarah Shelton, Inez Wynter, Siobhain Carmody, Valerie Joan Page, Claire Marie Beith, Karen Black, Suzanne Clements, Alan Morrison, Dominic Strachan, Margaret Taylor, Michelle Clarkson, Stuart D’Sylva, Kathryn Norman, Fiona Auld, Joanne Donnachie, Ian Edmond, Lynn Prentice, Nikole Runciman, Dario Salutous, Lesley Symon, Anne Todd, Patricia Turner, Abigail Short, Laura Sweeney, Euan Murdoch, Dhaneesha Senaratne, Michaela Hill, Thogulava Kannan, Laura Wild, Rikki Crawley, Abigail Crew, Mishell Cunningham, Allison Daniels, Laura Harrison, Susan Hope, Ken Inweregbu, Sian Jones, Nicola Lancaster, Jamie Matthews, Alice Nicholson, Gemma Wray, Helen Langton, Rachel Prout, Malcolm Watters, Catherine Novis, Anthony Barron, Ciara Collins, Sundeep Kaul, Heather Passmore, Claire Prendergast, Anna Reed, Paula Rogers, Rajvinder Shokkar, Meriel Woodruff, Hayley Middleton, Oliver Polgar, Claire Nolan, Vicky Thwaites, Kanta Mahay, Dawn Collier, Anil Hormis, Victoria Maynard, Cheryl Graham, Rachel Walker, Ellen Knights, Alicia Price, Alice Thomas, Chris Thorpe, Teresa Behan, Caroline Burnett, Jonathan Hatton, Elaine Heeney, Atideb Mitra, Maria Newton, Rachel Pollard, Rachael Stead, Vishal Amin, Elena Anastasescu, Vikram Anumakonda, Komala Karthik, Rizwana Kausar, Karen Reid, Jacqueline Smith, Janet Imeson-Wood, Alison Brown, Vikki Crickmore, Gabor Debreceni, Joy Wilkins, Liz Nicol, Waqas Khaliq, Rosie Reece-Anthony, Mark Birt, Alison Ghosh, Emma Williams, Louise Allen, Eva Beranova, Nikki Crisp, Joanne Deery, Tracy Hazelton, Alicia Knight, Carly Price, Sorrell Tilbey, Salah Turki, Sharon Turney, Joshua Cooper, Cheryl Finch, Sarah Liderth, Alison Quinn, Natalia Waddington, Tina Coventry, Susan Fowler, Michael MacMahon, Amanda McGregor, Anne Cowley, Judith Highgate, Alison Brown, Jane Gregory, Susan O’Connell, Tim Smith, Luigi Barberis, Shameer Gopal, Nichola Harris, Victoria Lake, Stella Metherell, Elizabeth Radford, Amelia Daniel, Joanne Finn, Rajnish Saha, Nikki White, Amy Easthope, Phil Donnison, Fiona Trim, Beena Eapen, Jenny Birch, Laura Bough, Josie Goodsell, Rebecca Tutton, Patricia Williams, Sarah Williams, Barbara Winter-Goodwin, Ailstair Nichol, Kathy Brickell, Michelle Smyth, Lorna Murphy, Samantha Coetzee, Alistair Gales, Igor Otahal, Meena Raj, Craig Sell, Paula Hilltout, Jayne Evitts, Amanda Tyler, Joanne Waldron, Kate Beesley, Sarah Board, Agnieszka Kubisz-Pudelko, Alison Lewis, Jess Perry, Lucy Pippard, Di Wood, Clare Buckley, Peter Barry, Neil Flint, Patel Rekha, Dawn Hales, Lara Bunni, Claire Jennings, Monica Latif, Rebecca Marshall, Gayathri Subramanian, Peter J. McGuigan, Christopher Wasson, Stephanie Finn, Jackie Green, Erin Collins, Bernadette King, Andy Campbell, Sara Smuts, Joseph Duffield, Oliver Smith, Lewis Mallon, Claire Watkins, Liam Botfield, Joanna Butler, Catherine Dexter, Jo Fletcher, Atul Garg, Aditya Kuravi, Poonam Ranga, Emma Virgilio, Zakaula Belagodu, Bridget Fuller, Anca Gherman, Olumide Olufuwa, Remi Paramsothy, Carmel Stuart, Naomi Oakley, Charlotte Kamundi, David Tyl, Katy Collins, Pedro Silva, June Taylor, Laura King, Charlotte Coates, Maria Crowley, Phillipa Wakefield, Jane Beadle, Laura Johnson, Janet Sargeant, Madeleine Anderson, Ailbhe Brady, Rebekah Chan, Jeff Little, Shane McIvor, Helena Prady, Helen Whittle, Bijoy Mathew, Ben Attwood, Penny Parsons, Geraldine Ward, Pamela Bremmer, West Joe, Baird Tracy, Ruddy Jim, Ellie Davies, Lisa Roche, Sonia Sathe, Catherine Dennis, Alastair McGregor, Victoria Parris, Sinduya Srikaran, Anisha Sukha, Rachael Campbell, Noreen Clarke, Jonathan Whiteside, Mairi Mascarenhas, Avril Donaldson, Joanna Matheson, Fiona Barrett, Marianne O’Hara, Laura Okeefe, Clare Bradley, Christine Eastgate-Jackson, Helder Filipe, Daniel Martin, Amitaa Maharajh, Sara Mingo Garcia, Glykeria Pakou, Mark De Neef, Kathy Dent, Elizabeth Horsley, Muhammad Nauman Akhtar, Sandra Pearson, Dorota Potoczna, Sue Spencer, Melanie Clapham, Rosemary Harper, Una Poultney, Polly Rice, Tim Smith, Rachel Mutch, Luigi Barberis, Lisa Armstrong, Hayley Bates, Emma Dooks, Fiona Farquhar, Brigid Hairsine, Chantal McParland, Sophie Packham, Rehana Bi, Barney Scholefield, Lydia Ashton, Linsha George, Sophie Twiss, David Wright, Manish Chablani, Amy Kirkby, Kimberley Netherton, Kim Davies, Linda O’Brien, Zohra Omar, Igor Otahal, Emma Perkins, Tracy Lewis, Isobel Sutherland, Karen Burns, Andrew Higham, Ben Chandler, Kerry Elliott, Janine Mallinson, Alison Turnbull, Prisca Gondo, Bernard Hadebe, Abdul Kayani, Bridgett Masunda, Taya Anderson, Dan Hawcutt, Laura O’Malley, Laura Rad, Naomi Rogers, Paula Saunderson, Kathryn Sian Allison, Deborah Afolabi, Jennifer Whitbread, Dawn Jones, Rachael Dore, Matthew Halkes, Pauline Mercer, Lorraine Thornton, Joy Dawson, Sweyn Garrioch, Melanie Tolson, Jonathan Aldridge, Ritoo Kapoor, David Loader, Karen Castle, Sally Humphreys, Ruth Tampsett, Katherine Mackintosh, Amanda Ayers, Wendy Harrison, Julie North, Suzanne Allibone, Roman Genetu, Vidya Kasipandian, Amit Patel, Ainhi Mac, Anthony Murphy, Parisa Mahjoob, Roonak Nazari, Lucy Worsley, Andrew Fagan, Thomas Bemand, Ethel Black, Arnold Dela Rosa, Ryan Howle, Shaman Jhanji, Ravishankar Rao Baikady, Kate Colette Tatham, Benjamin Thomas, Dina Bell, Rosalind Boyle, Katie Douglas, Lynn Glass, Emma Lee, Liz Lennon, Austin Rattray, Abigail Taylor, Rachel Anne Hughes, Helen Thomas, Alun Rees, Michaela Duskova, Janet Phipps, Suzanne Brooks, Michelle Edwards, Victoria Parris, Sheena Quaid, Ekaterina Watson, Adam Brayne, Emma Fisher, Jane Hunt, Peter Jackson, Duncan Kaye, Nicholas Love, Juliet Parkin, Victoria Tuckey, Lynne van Koutrik, Sasha Carter, Benedict Andrew, Louise Findlay, Katie Adams, Jen Service, Alison Williams, Claire Cheyne, Anne Saunderson, Sam Moultrie, Miranda Odam, Kathryn Hall, Isheunesu Mapfunde, Charlotte Willis, Alex Lyon, Chunda Sri-Chandana, Joslan Scherewode, Lorraine Stephenson, Sarah Marsh, David Brealey, John Hardy, Henry Houlden, Eleanor Moncur, Eamon Raith, Ambreen Tariq, Arianna Tucci, Maria Hobrok, Ronda Loosley, Heather McGuinness, Helen Tench, Rebecca Wolf-Roberts, Val Irvine, Benjamin Shelley, Amy Easthope, Claire Gorman, Abhinav Gupta, Elizabeth Timlick, Rebecca Brady, Barry Milligan, Arianna Bellini, Jade Bryant, Anton Mayer, Amy Pickard, Nicholas Roe, Jason Sowter, Alex Howlett, Katy Fidler, Emma Tagliavini, Kevin Donnelly, 23andMe investigators, Janie F. Shelton, Anjali J. Shastri, Chelsea Ye, Catherine H. Weldon, Teresa Filshtein-Sonmez, Daniella Coker, Antony Symons, Jorge Esparza-Gordillo, Stella Aslibekyan, Adam Auton, COVID-19 Human Genetics Initiative, Gita A. Pathak, Juha Karjalainen, Christine Stevens, Shea J. Andrews, Masahiro Kanai, Mattia Cordioli, Renato Polimanti, Matti Pirinen, Nadia Harerimana, Kumar Veerapen, Brooke Wolford, Huy Nguyen, Matthew Solomonson, Rachel G. Liao, Karolina Chwialkowska, Amy Trankiem, Mary K. Balaconis, Caroline Hayward, Anne Richmond, Archie Campbell, Marcela Morris, Chloe Fawns-Ritchie, Joseph T. Glessner, Douglas M. Shaw, Xiao Chang, Hannah Polikowski, Lauren E. Petty, Hung-Hsin Chen, Zhu Wanying, Hakon Hakonarson, David J. Porteous, Jennifer Below, Kari North, Joseph B. McCormick, Paul R. H. J. Timmers, James F. Wilson, Albert Tenesa, Kenton D’Mellow, Shona M. Kerr, Mari E. K. Niemi, Lindokuhle Nkambul, Kathrin Aprile von Hohenstaufen, Ali Sobh, Madonna M. Eltoukhy, Amr M. Yassen, Mohamed A. F. Hegazy, Kamal Okasha, Mohammed A. Eid, Hanteera S. Moahmed, Doaa Shahin, Yasser M. El-Sherbiny, Tamer A. Elhadidy, Mohamed S. Abd Elghafar, Jehan J. El-Jawhari, Attia A. S. Mohamed, Marwa H. Elnagdy, Amr Samir, Mahmoud Abdel-Aziz, Walid T. Khafaga, Walaa M. El-Lawaty, Mohamed S. Torky, Mohamed R. El-shanshory, Chiara Batini, Paul H. Lee, Nick Shrine, Alexander T. Williams, Martin D. Tobin, Anna L. Guyatt, Catherine John, Richard J. Packer, Altaf Ali, Robert C. Free, Xueyang Wang, Louise V. Wain, Edward J. Hollox, Laura D. Venn, Catherine E. Bee, Emma L. Adams, Ahmadreza Niavarani, Bahareh Sharififard, Rasoul Aliannejad, Ali Amirsavadkouhi, Zeinab Naderpour, Hengameh Ansari Tadi, Afshar Etemadi Aleagha, Saeideh Ahmadi, Seyed Behrooz Mohseni Moghaddam, Alireza Adamsara, Morteza Saeedi, Hamed Abdollahi, Abdolmajid Hosseini, Pajaree Chariyavilaskul, Monpat Chamnanphon, Thitima B. Suttichet, Vorasuk Shotelersuk, Monnat Pongpanich, Chureerat Phokaew, Wanna Chetruengchai, Watsamon Jantarabenjakul, Opass Putchareon, Pattama Torvorapanit, Thanyawee Puthanakit, Pintip Suchartlikitwong, Nattiya Hirankarn, Voraphoj Nilaratanakul, Pimpayao Sodsai, Ben M. Brumpton, Kristian Hveem, Cristen Willer, Wei Zhou, Tormod Rogne, Erik Solligard, Bjørn Olav Åsvold, Malak Abedalthagafi, Manal Alaamery, Saleh Alqahtani, Duna Barakeh, Fawz Al Harthi, Ebtehal Alsolm, Leen Abu Safieh, Albandary M. Alowayn, Fatimah Alqubaishi, Amal Al Mutairi, Serghei Mangul, Abdulraheem Alshareef, Mona Sawaji, Mansour Almutairi, Nora Aljawini, Nour Albesher, Yaseen M. Arabi, Ebrahim S. Mahmoud, Amin K. Khattab, Roaa T. Halawani, Ziab Z. Alahmadey, Jehad K. Albakri, Walaa A. Felemban, Bandar A. Suliman, Rana Hasanato, Laila Al-Awdah, Jahad Alghamdi, Deema AlZahrani, Sameera AlJohani, Hani Al-Afghani, May Alrashed, Nouf AlDhawi, Hadeel AlBardis, Sarah Alkwai, Moneera Alswailm, Faisal Almalki, Maha Albeladi, Iman Almohammed, Eman Barhoush, Anoud Albader, Salam Massadeh, Abdulaziz AlMalik, Sara Alotaibi, Bader Alghamdi, Junghyun Jung, Mohammad S. Fawzy, Yunsung Lee, Per Magnus, Lill-Iren S. Trogstad, Øyvind Helgeland, Jennifer R. Harris, Massimo Mangino, Tim D. Spector, Emma Duncan, Sandra P. Smieszek, Bartlomiej P. Przychodzen, Christos Polymeropoulos, Vasilios Polymeropoulos, Mihael H. Polymeropoulos, Israel Fernandez-Cadenas, Jordi Perez-Tur, Laia Llucià-Carol, Natalia Cullell, Elena Muiño, Jara Cárcel-Márquez, Marta L. DeDiego, Lara Lloret Iglesias, Anna M. Planas, Alex Soriano, Veronica Rico, Daiana Agüero, Josep L. Bedini, Francisco Lozano, Carlos Domingo, Veronica Robles, Francisca Ruiz-Jaén, Leonardo Márquez, Juan Gomez, Eliecer Coto, Guillermo M. Albaiceta, Marta García-Clemente, David Dalmau, Maria J. Arranz, Beatriz Dietl, Alex Serra-Llovich, Pere Soler, Roger Colobrán, Andrea Martín-Nalda, Alba Parra Martínez, David Bernardo, Silvia Rojo, Aida Fiz-López, Elisa Arribas, Paloma de la Cal-Sabater, Tomás Segura, Esther González-Villa, Gemma Serrano-Heras, Joan Martí-Fàbregas, Elena Jiménez-Xarrié, Alicia de Felipe Mimbrera, Jaime Masjuan, Sebastian García-Madrona, Anna Domínguez-Mayoral, Joan Montaner Villalonga, Paloma Menéndez-Valladares, Daniel I. Chasman, Julie E. Buring, Paul M. Ridker, Giulianini Franco, Howard D. Sesso, JoAnn E. Manson, Joseph R. Glessner, Hakon Hakonarson, Carolina Medina-Gomez, Andre G. Uitterlinden, M. Arfan Ikram, Kati Kristiansson, Sami Koskelainen, Markus Perola, Kati Donner, Katja Kivinen, Aarno Palotie, Samuli Ripatti, Sanni Ruotsalainen, Mari Kaunisto, Tomoko Nakanishi, Guillaume Butler-Laporte, Vincenzo Forgetta, David R. Morrison, Biswarup Ghosh, Laetitia Laurent, Alexandre Belisle, Danielle Henry, Tala Abdullah, Olumide Adeleye, Noor Mamlouk, Nofar Kimchi, Zaman Afrasiabi, Nardin Rezk, Branka Vulesevic, Meriem Bouab, Charlotte Guzman, Louis Petitjean, Chris Tselios, Xiaoqing Xue, Erwin Schurr, Jonathan Afilalo, Marc Afilalo, Maureen Oliveira, Bluma Brenner, Pierre Lepage, Jiannis Ragoussis, Daniel Auld, Nathalie Brassard, Madeleine Durand, Michaël Chassé, Daniel E. Kaufmann, G. Mark Lathrop, Vincent Mooser, J. Brent Richards, Rui Li, Darin Adra, Souad Rahmouni, Michel Georges, Michel Moutschen, Benoit Misset, Gilles Darcis, Julien Guiot, Julien Guntz, Samira Azarzar, Stéphanie Gofflot, Yves Beguin, Sabine Claassen, Olivier Malaise, Pascale Huynen, Christelle Meuris, Marie Thys, Jessica Jacques, Philippe Léonard, Frederic Frippiat, Jean-Baptiste Giot, Anne-Sophie Sauvage, Christian von Frenckell, Yasmine Belhaj, Bernard Lambermont, Sara Pigazzini, Lindokuhle Nkambule, Michelle Daya, Jonathan Shortt, Nicholas Rafaels, Stephen J. Wicks, Kristy Crooks, Kathleen C. Barnes, Christopher R. Gignoux, Sameer Chavan, Triin Laisk, Kristi Läll, Maarja Lepamets, Reedik Mägi, Tõnu Esko, Ene Reimann, Lili Milani, Helene Alavere, Kristjan Metsalu, Mairo Puusepp, Andres Metspalu, Paul Naaber, Edward Laane, Jaana Pesukova, Pärt Peterson, Kai Kisand, Jekaterina Tabri, Raili Allos, Kati Hensen, Joel Starkopf, Inge Ringmets, Anu Tamm, Anne Kallaste, Pierre-Yves Bochud, Carlo Rivolta, Stéphanie Bibert, Mathieu Quinodoz, Dhryata Kamdar, Noémie Boillat, Semira Gonseth Nussle, Werner Albrich, Noémie Suh, Dionysios Neofytos, Véronique Erard, Cathy Voide, Rafael de Cid, Iván Galván-Femenía, Natalia Blay, Anna Carreras, Beatriz Cortés, Xavier Farré, Lauro Sumoy, Victor Moreno, Josep Maria Mercader, Marta Guindo-Martinez, David Torrents, Manolis Kogevinas, Judith Garcia-Aymerich, Gemma Castaño-Vinyals, Carlota Dobaño, Alessandra Renieri, Francesca Mari, Chiara Fallerini, Sergio Daga, Elisa Benetti, Margherita Baldassarri, Francesca Fava, Elisa Frullanti, Floriana Valentino, Gabriella Doddato, Annarita Giliberti, Rossella Tita, Sara Amitrano, Mirella Bruttini, Susanna Croci, Ilaria Meloni, Maria Antonietta Mencarelli, Caterina Lo Rizzo, Anna Maria Pinto, Giada Beligni, Andrea Tommasi, Laura Di Sarno, Maria Palmieri, Miriam Lucia Carriero, Diana Alaverdian, Stefano Busani, Raffaele Bruno, Marco Vecchia, Mary Ann Belli, Nicola Picchiotti, Maurizio Sanarico, Marco Gori, Simone Furini, Stefania Mantovani, Serena Ludovisi, Mario Umberto Mondelli, Francesco Castelli, Eugenia Quiros-Roldan, Melania Degli Antoni, Isabella Zanella, Massimo Vaghi, Stefano Rusconi, Matteo Siano, Francesca Montagnani, Arianna Emiliozzi, Massimiliano Fabbiani, Barbara Rossetti, Elena Bargagli, Laura Bergantini, Miriana D’Alessandro, Paolo Cameli, David Bennett, Federico Anedda, Simona Marcantonio, Sabino Scolletta, Federico Franchi, Maria Antonietta Mazzei, Susanna Guerrini, Edoardo Conticini, Luca Cantarini, Bruno Frediani, Danilo Tacconi, Chiara Spertilli, Marco Feri, Alice Donati, Raffaele Scala, Luca Guidelli, Genni Spargi, Marta Corridi, Cesira Nencioni, Leonardo Croci, Maria Bandini, Gian Piero Caldarelli, Paolo Piacentini, Elena Desanctis, Silvia Cappelli, Anna Canaccini, Agnese Verzuri, Valentina Anemoli, Agostino Ognibene, Alessandro Pancrazzi, Maria Lorubbio, Antonella D’Arminio Monforte, Federica Gaia Miraglia, Massimo Girardis, Sophie Venturelli, Andrea Cossarizza, Andrea Antinori, Alessandra Vergori, Arianna Gabrieli, Agostino Riva, Daniela Francisci, Elisabetta Schiaroli, Francesco Paciosi, Pier Giorgio Scotton, Francesca Andretta, Sandro Panese, Renzo Scaggiante, Francesca Gatti, Saverio Giuseppe Parisi, Stefano Baratti, Matteo Della Monica, Carmelo Piscopo, Mario Capasso, Roberta Russo, Immacolata Andolfo, Achille Iolascon, Giuseppe Fiorentino, Massimo Carella, Marco Castori, Giuseppe Merla, Gabriella Maria Squeo, Filippo Aucella, Pamela Raggi, Carmen Marciano, Rita Perna, Matteo Bassetti, Antonio Di Biagio, Maurizio Sanguinetti, Luca Masucci, Serafina Valente, Marco Mandalà, Alessia Giorli, Lorenzo Salerni, Patrizia Zucchi, Pierpaolo Parravicini, Elisabetta Menatti, Tullio Trotta, Ferdinando Giannattasio, Gabriella Coiro, Fabio Lena, Domenico A. Coviello, Cristina Mussini, Enrico Martinelli, Sandro Mancarella, Luisa Tavecchia, Lia Crotti, Chiara Gabbi, Marco Rizzi, Franco Maggiolo, Diego Ripamonti, Tiziana Bachetti, Maria Teresa La Rovere, Simona Sarzi-Braga, Maurizio Bussotti, Stefano Ceri, Pietro Pinoli, Francesco Raimondi, Filippo Biscarini, Alessandra Stella, Kristina Zguro, Katia Capitani, Claudia Suardi, Simona Dei, Gianfranco Parati, Sabrina Ravaglia, Rosangela Artuso, Giordano Bottà, Paolo Di Domenico, Ilaria Rancan, Antonio Perrella, Francesco Bianchi, Davide Romani, Paola Bergomi, Emanuele Catena, Riccardo Colombo, Marco Tanfoni, Antonella Vincenti, Claudio Ferri, Davide Grassi, Gloria Pessina, Mario Tumbarello, Massimo Di Pietro, Ravaglia Sabrina, Sauro Luchi, Chiara Barbieri, Donatella Acquilini, Elena Andreucci, Francesco Vladimiro Segala, Giusy Tiseo, Marco Falcone, Mirjam Lista, Monica Poscente, Oreste De Vivo, Paola Petrocelli, Alessandra Guarnaccia, Silvia Baroni, Albert V. Smith, Andrew P. Boughton, Kevin W. Li, Jonathon LeFaive, Aubrey Annis, Anne E. Justice, Tooraj Mirshahi, Geetha Chittoor, Navya Shilpa Josyula, Jack A. Kosmicki, Manuel A. R. Ferreira, Joseph B. Leader, Dave J. Carey, Matthew C. Gass, Julie E. Horowitz, Michael N. Cantor, Ashish Yadav, Aris Baras, Goncalo R. Abecasis, David A. van Heel, Karen A. Hunt, Dan Mason, Qin Qin Huang, Sarah Finer, Bhavi Trivedi, Christopher J. Griffiths, Hilary C. Martin, John Wright, Richard C. Trembath, Nicole Soranzo, Jing Hua Zhao, Adam S. Butterworth, John Danesh, Emanuele Di Angelantonio, Lude Franke, Marike Boezen, Patrick Deelen, Annique Claringbould, Esteban Lopera, Robert Warmerdam, Judith M. Vonk, Irene van Blokland, Pauline Lanting, Anil P. S. Ori, Sebastian Zöllner, Jiongming Wang, Andrew Beck, Gina Peloso, Yuk-Lam Ho, Yan V. Sun, Jennifer E. Huffman, Christopher J. O’Donnell, Kelly Cho, Phil Tsao, J. Michael Gaziano, Michel Nivard, Eco de Geus, Meike Bartels, Jouke Jan Hottenga, Scott T. Weiss, Elizabeth W. Karlson, Jordan W. Smoller, Robert C. Green, Yen-Chen Anne Feng, Josep Mercader, Shawn N. Murphy, James B. Meigs, Ann E. Woolley, Emma F. Perez, Daniel Rader, Anurag Verma, Marylyn D. Ritchie, Binglan Li, Shefali S. Verma, Anastasia Lucas, Yuki Bradford, Hugo Zeberg, Robert Frithiof, Michael Hultström, Miklos Lipcsey, Lindo Nkambul, Nicolas Tardif, Olav Rooyackers, Jonathan Grip, Tomislav Maricic, Konrad J. Karczewski, Elizabeth G. Atkinson, Kristin Tsuo, Nikolas Baya, Patrick Turley, Rahul Gupta, Shawneequa Callier, Raymond K. Walters, Duncan S. Palmer, Gopal Sarma, Nathan Cheng, Wenhan Lu, Sam Bryant, Claire Churchhouse, Caroline Cusick, Jacqueline I. Goldstein, Daniel King, Cotton Seed, Hilary Finucane, Alicia R. Martin, F. Kyle Satterstrom, Daniel J. Wilson, Jacob Armstrong, Justine K. Rudkin, Gavin Band, Sarah G. Earle, Shang-Kuan Lin, Nicolas Arning, Derrick W. Crook, David H. Wyllie, Anne Marie O’Connell, Chris C. A. Spencer, Nils Koelling, Mark J. Caulfield, Richard H. Scott, Tom Fowler, Loukas Moutsianas, Athanasios Kousathanas, Dorota Pasko, Susan Walker, Augusto Rendon, Alex Stuckey, Christopher A. Odhams, Daniel Rhodes, Georgia Chan, Prabhu Arumugam, Catherine A. Ball, Eurie L. Hong, Kristin Rand, Ahna Girshick, Harendra Guturu, Asher Haug Baltzell, Genevieve Roberts, Danny Park, Marie Coignet, Shannon McCurdy, Spencer Knight, Raghavendran Partha, Brooke Rhead, Miao Zhang, Nathan Berkowitz, Michael Gaddis, Keith Noto, Luong Ruiz, Milos Pavlovic, Laura G. Sloofman, Alexander W. Charney, Noam D. Beckmann, Eric E. Schadt, Daniel M. Jordan, Ryan C. Thompson, Kyle Gettler, Noura S. Abul-Husn, Steven Ascolillo, Joseph D. Buxbaum, Kumardeep Chaudhary, Judy H. Cho, Yuval Itan, Eimear E. Kenny, Gillian M. Belbin, Stuart C. Sealfon, Robert P. Sebra, Irene Salib, Brett L. Collins, Tess Levy, Bari Britvan, Katherine Keller, Lara Tang, Michael Peruggia, Liam L. Hiester, Kristi Niblo, Alexandra Aksentijevich, Alexander Labkowsky, Avrohom Karp, Menachem Zlatopolsky, Michael Preuss, Ruth J. F. Loos, Girish N. Nadkarni, Ron Do, Clive Hoggart, Sam Choi, Slayton J. Underwood, Paul O’Reilly, Laura M. Huckins, Marissa Zyndorf, Mark J. Daly, Benjamin M. Neale, Andrea Ganna, Angie Fawkes, Lee Murphy, Kathy Rowan, Chris P. Ponting, Veronique Vitart, James F. Wilson, Jian Yang, Andrew D. Bretherick, Richard H. Scott, Sara Clohisey Hendry, Loukas Moutsianas, Andy Law, Mark J. Caulfield, J. Kenneth Baillie

**Affiliations:** 1 Genomics England, London, UK; 2 Roslin Institute, University of Edinburgh, Edinburgh, UK; 3 MRC Human Genetics Unit, Institute of Genetics and Cancer, University of Edinburgh, Western General Hospital, Edinburgh, UK; 4 Centre for Inflammation Research, The Queen’s Medical Research Institute, University of Edinburgh, Edinburgh, UK; 5 Wellcome Centre for Human Genetics, University of Oxford, Oxford, UK; 6 Institute for Molecular Bioscience, The University of Queensland, Brisbane, Queensland Australia; 7 Biostatistics Group, Greater Bay Area Institute of Precision Medicine (Guangzhou), Fudan University, Guangzhou, China; 8 Centre for Global Health Research, Usher Institute of Population Health Sciences and Informatics, Edinburgh, UK; 9 Edinburgh Clinical Research Facility, Western General Hospital, University of Edinburgh, Edinburgh, UK; 10 Intensive Care Unit, Royal Infirmary of Edinburgh, Edinburgh, UK; 11 Department of Critical Care Medicine, Queen’s University and Kingston Health Sciences Centre, Kingston, Ontario Canada; 12 Clinical Research Centre at St Vincent’s University Hospital, University College Dublin, Dublin, Ireland; 13 NIHR Health Protection Research Unit for Emerging and Zoonotic Infections, Institute of Infection, Veterinary and Ecological Sciences, University of Liverpool, Liverpool, UK; 14 Respiratory Medicine and Institute in the Park, Alder Hey Children’s Hospital and University of Liverpool, Liverpool, UK; 15 Illumina Cambridge, Great Abington, UK; 16 Regeneron Genetics Center, Tarrytown, NY USA; 17 Geisinger, Danville, PA USA; 18 Department of Genetics, Perelman School of Medicine, University of Pennsylvania, Philadelphia, PA USA; 19 Test and Trace, the Health Security Agency, Department of Health and Social Care, London, UK; 20 Department of Intensive Care Medicine, Guy’s and St Thomas’ NHS Foundation Trust, London, UK; 21 Department of Medicine, University of Cambridge, Cambridge, UK; 22 William Harvey Research Institute, Barts and the London School of Medicine and Dentistry, Queen Mary University of London, London, UK; 23 Centre for Tropical Medicine and Global Health, Nuffield Department of Medicine, University of Oxford, Oxford, UK; 24 Department of Anaesthesia and Intensive Care, The Chinese University of Hong Kong, Prince of Wales Hospital, Hong Kong, China; 25 Wellcome–Wolfson Institute for Experimental Medicine, Queen’s University Belfast, Belfast, UK; 26 Department of Intensive Care Medicine, Royal Victoria Hospital, Belfast, UK; 27 UCL Centre for Human Health and Performance, London, UK; 28 National Heart and Lung Institute, Imperial College London, London, UK; 29 Imperial College Healthcare NHS Trust: London, London, UK; 30 Imperial College, London, UK; 31 Intensive Care National Audit and Research Centre, London, UK; 32 School of Life Sciences, Westlake University, Hangzhou, China; 33 Westlake Laboratory of Life Sciences and Biomedicine, Hangzhou, China; 34 Great Ormond Street Hospital, London, UK; 35 William Harvey Research Institute, Queen Mary University of London, London, UK; 36 Roslin Institute, University of Edinburgh, Edinburgh, UK; 37 Intensive Care Unit, Royal Infirmary of Edinburgh, Edinburgh, UK; 38 Royal Hospital for Children, Glasgow, UK; 39 William Harvey Research Institute, Barts and the London School of Medicine and Dentistry, Queen Mary University of London, London, UK; 40 Centre for Tropical Medicine and Global Health, Nuffield Department of Medicine, University of Oxford, Oxford, UK; 41 Wellcome Centre for Human Genetics, University of Oxford, Oxford, UK; 42 Prince of Wales Hospital, Hong Kong, China; 43 Department of Critical Care Medicine, Queen’s University and Kingston Health Sciences Centre, Kingston, Ontario Canada; 44 Wellcome–Wolfson Institute for Experimental Medicine, Queen’s University Belfast, Belfast, UK; 45 Department of Intensive Care Medicine, Royal Victoria Hospital, Belfast, UK; 46 UCL Centre for Human Health and Performance, London, UK; 47 Clinical Research Centre at St Vincent’s University Hospital, University College Dublin, Dublin, Ireland; 48 National Heart and Lung Institute, Imperial College London, London, UK; 49 Imperial College Healthcare NHS Trust: London, London, UK; 50 Heart Institute, University of São Paulo, São Paulo, Brazil; 51 MRC Human Genetics Unit, Institute of Genetics and Molecular Medicine, University of Edinburgh, Western General Hospital, Edinburgh, UK; 52 Intensive Care National Audit and Research Centre, London, UK; 53 NIHR Health Protection Research Unit for Emerging and Zoonotic Infections, Institute of Infection, Veterinary and Ecological Sciences, University of Liverpool, Liverpool, UK; 54 Respiratory Medicine and Institute in the Park, Alder Hey Children’s Hospital and University of Liverpool, Liverpool, UK; 55 Department of Intensive Care Medicine, Guy’s and St Thomas’ NHS Foundation Trust, London, UK; 56 Department of Medicine, University of Cambridge, Cambridge, UK; 57 NIHR Clinical Research Network (CRN), Hammersmith Hospital, London, UK; 58 Cambridge University Hospitals NHS Foundation Trust, Cambridge, UK; 59 Edinburgh Clinical Research Facility, Western General Hospital, University of Edinburgh, Edinburgh, UK; 60 Biostatistics Group, State Key Laboratory of Biocontrol, School of Life Sciences, Sun Yat-sen University, Guangzhou, China; 61 Department of Infectious Diseases, Leiden University Medical Center, Leiden, The Netherlands; 62 Guys and St Thomas’ Hospital, London, UK; 63 Barts Health NHS Trust, London, UK; 64 James Cook University Hospital, Middlesbrough, UK; 65 Royal Stoke University Hospital, Stoke-on-Trent, UK; 66 North Middlesex University Hospital NHS Trust, London, UK; 67 North Middlesex University Hospital NHS Trust, London, UK; 68 The Royal Liverpool University Hospital, Liverpool, UK; 69 King’s College Hospital, London, UK; 70 Charing Cross Hospital, St Mary’s Hospital and Hammersmith Hospital, London, UK; 71 Nottingham University Hospital, Nottingham, UK; 72 John Radcliffe Hospital, Oxford, UK; 73 Kingston Hospital, Kingston-upon-Thames, UK; 74 Kingston Hospital, Kingston-upon-Thames, UK; 75 Royal Infirmary of Edinburgh, Edinburgh, UK; 76 Queen Alexandra Hospital, Portsmouth, UK; 77 Morriston Hospital, Swansea, UK; 78 Addenbrooke’s Hospital, Cambridge, UK; 79 BHRUT (Barking Havering)—Queen’s Hospital and King George Hospital, Romford, UK; 80 Royal Sussex County Hospital, Brighton, UK; 81 Queen Elizabeth Hospital, Birmingham, UK; 82 Queen Elizabeth Hospital, Woolwich, London, UK; 83 St George’s Hospital, London, UK; 84 Stepping Hill Hospital, Stockport, UK; 85 Countess of Chester Hospital, Chester, UK; 86 Royal Blackburn Teaching Hospital, Blackburn, UK; 87 The Tunbridge Wells Hospital and Maidstone Hospital, Tunbridge Wells, UK; 88 Royal Gwent Hospital, Newport, UK; 89 Pinderfields General Hospital, Wakefield, UK; 90 Royal Berkshire NHS Foundation Trust, Reading, UK; 91 Broomfield Hospital, Chelmsford, UK; 92 Northumbria Healthcare NHS Foundation Trust, North Shields, UK; 93 Whiston Hospital, Prescot, UK; 94 Croydon University Hospital, Croydon, UK; 95 York Hospital, York, UK; 96 Heartlands Hospital, Birmingham, UK; 97 Ashford and St Peter’s Hospital, Chertsey, UK; 98 Barnet Hospital, London, UK; 99 East Surrey Hospital, Redhill, UK; 100 Ninewells Hospital, Dundee, UK; 101 Worthing Hospital, Worthing, UK; 102 St Richard’s Hospital, Chichester, UK; 103 Southampton General Hospital, Southampton, UK; 104 The Alexandra Hospital, Redditch and Worcester Royal Hospital, Worcester, UK; 105 Sandwell General Hospital and City Hospital, Birmingham, UK; 106 Blackpool Victoria Hospital, Blackpool, UK; 107 Royal Glamorgan Hospital, Pontyclun, UK; 108 The Royal Oldham Hospital, Manchester, UK; 109 Glasgow Royal Infirmary, Glasgow, UK; 110 St James’s University Hospital and Leeds General Infirmary, Leeds, UK; 111 University Hospital North Durham, Durham, UK; 112 Darlington Memorial Hospital, Darlington, UK; 113 Fairfield General Hospital, Bury, UK; 114 Wythenshawe Hospital, Manchester, UK; 115 Royal Alexandra Hospital, Paisley, UK; 116 Good Hope Hospital, Birmingham, UK; 117 Tameside General Hospital, Ashton-under-Lyne, UK; 118 Royal Derby Hospital, Derby, UK; 119 Medway Maritime Hospital, Gillingham, UK; 120 Royal Victoria Infirmary, Newcastle-upon-Tyne, UK; 121 Poole Hospital, Poole, UK; 122 Bedford Hospital, Bedford, UK; 123 Queens Hospital Burton, Burton-on-Trent, UK; 124 North Manchester General Hospital, Manchester, UK; 125 Aberdeen Royal Infirmary, Aberdeen, UK; 126 Derriford Hospital, Plymouth, UK; 127 Manchester Royal Infirmary, Manchester, UK; 128 Salford Royal Hospital, Manchester, UK; 129 William Harvey Hospital, Ashford, UK; 130 Queen Elizabeth University Hospital, Glasgow, UK; 131 Bradford Royal Infirmary, Bradford, UK; 132 Bristol Royal Infirmary, Bristol, UK; 133 Norfolk and Norwich University Hospital (NNUH), Norwich, UK; 134 Queen Elizabeth Hospital Gateshead, Gateshead, UK; 135 Sunderland Royal Hospital, Sunderland, UK; 136 Aintree University Hospital, Liverpool, UK; 137 Hull Royal Infirmary, Hull, UK; 138 Hull Royal Infirmary, Hull, UK; 139 University College Hospital, London, UK; 140 Royal Devon and Exeter Hospital, Exeter, UK; 141 The Royal Papworth Hospital, Cambridge, UK; 142 Ipswich Hospital, Ipswich, UK; 143 Southmead Hospital, Bristol, UK; 144 Milton Keynes University Hospital, Milton Keynes, UK; 145 Royal Hampshire County Hospital, Winchester, UK; 146 Great Ormond St Hospital and UCL Great Ormond St Institute of Child Health NIHR Biomedical Research Centre, London, UK; 147 Stoke Mandeville Hospital, Aylesbury, UK; 148 University Hospital of Wales, Cardiff, UK; 149 Basingstoke and North Hampshire Hospital, Basingstoke, UK; 150 Arrowe Park Hospital, Wirral, UK; 151 Chesterfield Royal Hospital Foundation Trust, Chesterfield, UK; 152 Musgrove Park Hospital, Taunton, UK; 153 Peterborough City Hospital, Peterborough, UK; 154 Hinchingbrooke Hospital, Huntingdon, UK; 155 Royal Hallamshire Hospital and Northern General Hospital, Sheffield, UK; 156 Dumfries and Galloway Royal Infirmary, Dumfries, UK; 157 Royal Bolton Hospital, Bolton, UK; 158 Lister Hospital, Stevenage, UK; 159 Craigavon Area Hospital, Craigavon, UK; 160 Southport and Formby District General Hospital, Ormskirk, UK; 161 Calderdale Royal Hospital, Halifax, UK; 162 Huddersfield Royal Infirmary, Huddersfield, UK; 163 Prince Charles Hospital, Merthyr Tydfil, UK; 164 Royal Bournemouth Hospital, Bournemouth, UK; 165 Royal Preston Hospital, Preston, UK; 166 Whittington Hospital, London, UK; 167 Princess Royal Hospital, Telford and Royal Shrewsbury Hospital, Shrewsbury, UK; 168 Princess Royal Hospital, Haywards Heath, UK; 169 Macclesfield District General Hospital, Macclesfield, UK; 170 Royal Surrey County Hospital, Guildford, UK; 171 Hereford County Hospital, Hereford, UK; 172 University Hospital of North Tees, Stockton-on-Tees, UK; 173 Lincoln County Hospital, Lincoln, UK; 174 Royal Cornwall Hospital, Truro, UK; 175 Royal United Hospital, Bath, UK; 176 Royal Brompton Hospital, London, UK; 177 University Hospital Crosshouse, Kilmarnock, UK; 178 Basildon Hospital, Basildon, UK; 179 Glan Clwyd Hospital, Bodelwyddan, UK; 180 West Middlesex Hospital, Isleworth, UK; 181 Royal Lancaster Infirmary, Lancaster, UK; 182 Western General Hospital, Edinburgh, UK; 183 Chelsea and Westminster NHS Foundation Trust, London, UK; 184 The Queen Elizabeth Hospital, King’s Lynn, UK; 185 King’s Mill Hospital, Nottingham, UK; 186 Watford General Hospital, Watford, UK; 187 University Hospital Wishaw, Wishaw, UK; 188 Forth Valley Royal Hospital, Falkirk, UK; 189 George Eliot Hospital NHS Trust, Nuneaton, UK; 190 Barnsley Hospital, Barnsley, UK; 191 The Great Western Hospital, Swindon, UK; 192 Harefield Hospital, London, UK; 193 Rotherham General Hospital, Rotherham, UK; 194 Ysbyty Gwynedd, Bangor, UK; 195 Diana Princess of Wales Hospital, Grimsby, UK; 196 Russell’s Hall Hospital, Dudley, UK; 197 St Mary’s Hospital, Newport, UK; 198 University Hospital Lewisham, London, UK; 199 Colchester General Hospital, Colchester, UK; 200 Queen Elizabeth the Queen Mother Hospital, Margate, UK; 201 Royal Albert Edward Infirmary, Wigan, UK; 202 Victoria Hospital, Kirkcaldy, UK; 203 Eastbourne District General Hospital, Eastbourne, UK; 204 Conquest Hospital, St Leonards-on-Sea, UK; 205 Cumberland Infirmary, Carlisle, UK; 206 New Cross Hospital, Wolverhampton, UK; 207 The Princess Alexandra Hospital, Harlow, UK; 208 Salisbury District Hospital, Salisbury, UK; 209 Dorset County Hospital, Dorchester, UK; 210 University College Dublin, St Vincent’s University Hospital, Dublin, Ireland; 211 Glangwili General Hospital, Carmarthen, UK; 212 Gloucestershire Royal Hospital, Gloucester, UK; 213 Yeovil Hospital, Yeovil, UK; 214 Leicester Royal Infirmary, Leicester, UK; 215 Royal Manchester Children’s Hospital, Manchester, UK; 216 Royal Victoria Hospital, Belfast, UK; 217 Wrexham Maelor Hospital, Wrexham, UK; 218 Walsall Manor Hospital, Walsall, UK; 219 Darent Valley Hospital, Dartford, UK; 220 Warrington General Hospital, Warrington, UK; 221 Warwick Hospital, Warwick, UK; 222 University Hospitals Coventry and Warwickshire NHS Trust, Coventry, UK; 223 University Hospital Monklands, Airdrie, UK; 224 Princess of Wales Hospital, Llantrisant, UK; 225 Northwick Park Hospital, London, UK; 226 Raigmore Hospital, Inverness, UK; 227 Royal Free Hospital, London, UK; 228 Scunthorpe General Hospital, Scunthorpe, UK; 229 West Cumberland Hospital, Whitehaven, UK; 230 Airedale General Hospital, Keighley, UK; 231 Birmingham Children’s Hospital, Birmingham, UK; 232 Liverpool Heart and Chest Hospital, Liverpool, UK; 233 Pilgrim Hospital, Lincoln, UK; 234 Prince Philip Hospital, Llanelli, UK; 235 Furness General Hospital, Barrow-in-Furness, UK; 236 Scarborough General Hospital, Scarborough, UK; 237 Southend University Hospital, Westcliff-on-Sea, UK; 238 Alder Hey Children’s Hospital, Liverpool, UK; 239 Torbay Hospital, Torquay, UK; 240 Borders General Hospital, Melrose, UK; 241 Kent and Canterbury Hospital, Canterbury, UK; 242 West Suffolk Hospital, Bury St Edmunds, UK; 243 James Paget University Hospital NHS Trust, Great Yarmouth, UK; 244 The Christie NHS Foundation Trust, Manchester, UK; 245 The Royal Marsden Hospital, London, UK; 246 University Hospital Hairmyres, East Kilbride, UK; 247 Withybush General Hospital, Haverfordwest, Wales UK; 248 Ealing Hospital, Southall, UK; 249 North Devon District Hospital, Barnstaple, UK; 250 St John’s Hospital Livingston, Livingston, UK; 251 Northampton General Hospital NHS Trust, Northampton, UK; 252 Harrogate and District NHS Foundation Trust, Harrogate, UK; 253 National Hospital for Neurology and Neurosurgery, London, UK; 254 Bronglais General Hospital, Aberystwyth, UK; 255 Golden Jubilee National Hospital, Clydebank, UK; 256 Homerton University Hospital Foundation NHS Trust, London, UK; 257 Sheffield Children’s Hospital, Sheffield, UK; 258 The Royal Alexandra Children’s Hospital, Brighton, UK; 259 23andMe, Sunnyvale, CA USA; 260 Human Genetics R&D and Target Sciences R&D, GSK Medicines Research Centre, Stevenage, UK; 261 Yale University, New Haven, CT USA; 262 Institute for Molecular Medicine Finland (FIMM), University of Helsinki, Helsinki, Finland; 263 Broad Institute of MIT and Harvard, Cambridge, MA USA; 264 Icahn School of Medicine at Mount Sinai, New York, NY USA; 265 University of Michigan, Ann Arbor, MI USA; 266 Centre for Bioinformatics and Data Analysis, Medical University of Bialystok, Bialystok, Poland; 267 Institute of Genetics and Cancer, University of Edinburgh, Western General Hospital, Edinburgh, UK; 268 University of Texas Health, Houston, TX USA; 269 Center for Applied Genomics, Children’s Hospital of Philadelphia, Philadelphia, PA USA; 270 Department of Pediatrics, Perelman School of Medicine, University of Pennsylvania, Philadelphia, PA USA; 271 Vanderbilt University Medical Center, Nashville, TN USA; 272 University of North Carolina at Chapel Hill, Chapel Hill, NC USA; 273 Roslin Institute, The Royal (Dick) School of Veterinary Studies, University of Edinburgh, Edinburgh, UK; 274 Analytic and Translational Genetics Unit, Massachusetts General Hospital, Boston, MA USA; 275 Genolier Innovation Network and Hub, Swiss Medical Network, Genolier Healthcare Campus, Genolier, Switzerland; 276 Department of Pediatrics, Faculty of Medicine, Mansoura University, Mansoura, Egypt; 277 Department of Clinical Pathology, Faculty of Medicine, Tanta University, Tanta, Egypt; 278 Department of Anaethesia and Critical Care, Faculty of Medicine, Mansoura University, Mansoura, Egypt; 279 Department of Surgery, Faculty of Medicine, Mansoura University, Mansoura, Egypt; 280 Department of Internal Medicine, Faculty of Medicine, Tanta University, Tanta, Egypt; 281 Faculty of Science, Tanta University, Tanta, Egypt; 282 Chest Department, Faculty of Medicine, Tanta University, Tanta, Egypt; 283 Department of Clinical Pathology, Faculty of Medicine, Mansoura University, Mansoura, Egypt; 284 Department of Biosciences, School of Science and Technology, Nottingham Trent University, Nottingham, UK; 285 Chest Department, Faculty of Medicine, Mansoura University, Mansoura, Egypt; 286 Anesthesia, Surgical Intensive Care and Pain Management Department, Faculty of Medicine, Tanta University, Tanta, Egypt; 287 Department of Medical Biochemistry, Faculty of Medicine, Mansoura University, Mansoura, Egypt; 288 Department of Tropical Medicine, Faculty of Medicine, Mansoura University, Mansoura, Egypt; 289 Pediatric and Neonatology, Kafr El-Zayat General Hospital, Kafr El-Zayat, Egypt; 290 Pediatrics Department, Faculty of Medicine, Tanta University, Tanta, Egypt; 291 Department of Health Sciences, University of Leicester, Leicester, UK; 292 Leicester NIHR Biomedical Research Centre, Leicester, UK; 293 Department of Respiratory Sciences, University of Leicester, Leicester, UK; 294 University of Leicester, Leicester, UK; 295 Digestive Oncology Research Center, Digestive Disease Research Institute, Shariati Hospital, Tehran University of Medical Sciences, Tehran, Iran; 296 Department of Pulmonology, School of Medicine, Shariati Hospital, Tehran University of Medical Sciences, Tehran, Iran; 297 Department of Critical Care Medicine, Noorafshar Hospital, Tehran, Iran; 298 Department of Emergency Intensive Care Unit, School of Medicine, Shariati Hospital, Tehran University of Medical Sciences, Tehran, Iran; 299 Department of Anesthesiology, School of Medicine, Amir Alam Hospital, Tehran University of Medical Sciences, Tehran, Iran; 300 Department of Pulmonology, School of Medicine, Tehran University of Medical Sciences, Tehran, Iran; 301 Department of Pathology, Parseh Pathobiology and Genetics Laboratory, Tehran, Iran; 302 Department of Microbiology, Health and Family Research Center, NIOC Hospital, Tehran, Iran; 303 Department of Emergency Medicine, School of Medicine, Shariati Hospital, Tehran University of Medical Sciences, Tehran, Iran; 304 Department of Anesthesiology, School of Medicine, Tehran University of Medical Sciences, Tehran, Iran; 305 Department of Pathology, Faculty of Medicine, Tehran Azad University, Tehran, Iran; 306 Clinical Pharmacokinetics and Pharmacogenomics Research Unit, Faculty of Medicine, Chulalongkorn University, Bangkok, Thailand; 307 Department of Pharmacology, Faculty of Medicine, Chulalongkorn University, Bangkok, Thailand; 308 Department of Pathology, Faculty of Medicine, Nakornnayok, Srinakharinwirot University, Bangkok, Thailand; 309 Center of Excellence for Medical Genomics, Medical Genomics Cluster, Faculty of Medicine, Chulalongkorn University, Bangkok, Thailand; 310 Excellence Center for Genomics and Precision Medicine, King Chulalongkorn Memorial Hospital, The Thai Red Cross Society, Bangkok, Thailand; 311 Department of Mathematics and Computer Science, Faculty of Science, Chulalongkorn University, Bangkok, Thailand; 312 Omics Sciences and Bioinfomatics Center, Faculty of Science, Chulalongkorn University, Bangkok, Thailand; 313 Research Affairs, Faculty of Medicine, Chulalongkorn University, Bangkok, Thailand; 314 Thai Red Cross Emerging Infectious Diseases Clinical Centre, King Chulalongkorn Memorial Hospital, Bangkok, Thailand; 315 Department of Pediatrics, Faculty of Medicine, Chulalongkorn University, Bangkok, Thailand; 316 Division of Infectious Diseases, Department of Medicine, Faculty of Medicine, Chulalongkorn University, Bangkok, Thailand; 317 Center of Excellence in Pediatric Infectious Diseases and Vaccines, Chulalongkorn University, Bangkok, Thailand; 318 Department of Microbiology, Faculty of Medicine, Chulalongkorn University, Bangkok, Thailand; 319 Immunology Division, Department of Microbiology, Faculty of Medicine, Chulalongkorn University, Bangkok, Thailand; 320 Center of Excellence in Immunology and Immune-mediated Diseases, Department of Microbiology, Faculty of Medicine, Chulalongkorn University, Bangkok, Thailand; 321 Healthcare-associated Infection Research Group STAR (Special Task Force for Activating Research), Chulalongkorn University, Bangkok, Thailand; 322 K.G. Jebsen Center for Genetic Epidemiology, Department of Public Health and Nursing, Norwegian University of Science and Technology (NTNU), Trondheim, Norway; 323 HUNT Research Center, Department of Public Health and Nursing, Norwegian University of Science and Technology (NTNU), Levanger, Norway; 324 Clinic of Medicine, St Olav’s Hospital, Trondheim University Hospital, Trondheim, Norway; 325 Division of Cardiovascular Medicine, Department of Internal Medicine, University of Michigan, Ann Arbor, MI USA; 326 Department of Computational Medicine and Bioinformatics, University of Michigan, Ann Arbor, MI USA; 327 Program in Medical and Population Genetics, Broad Institute of Harvard and MIT, Cambridge, MA USA; 328 Gemini Center for Sepsis Research, Department of Circulation and Medical Imaging, Norwegian University of Science and Technology (NTNU), Trondheim, Norway; 329 Department of Chronic Disease Epidemiology and Center for Perinatal, Pediatric and Environmental Epidemiology, Yale School of Public Health, New Haven, CT USA; 330 Clinic of Anaesthesia and Intensive Care, St Olav’s Hospital, Trondheim University Hospital, Trondheim, Norway; 331 Genomics Research Department, Saudi Human Genome Project, King Fahad Medical City and King Abdulaziz City for Science and Technology (KACST), Riyadh, Saudi Arabia; 332 Developmental Medicine Department, King Abdullah International Medical Research Center, King Saud Bin Abdulaziz University for Health Sciences, Ministry of National Guard Health Affairs, Riyadh, Saudi Arabia; 333 Saudi Human Genome Project (SHGP), King Abdulaziz City for Science and Technology (KACST), Satellite Lab at King Abdulaziz Medical City, Ministry of National Guard Health Affairs, Riyadh, Saudi Arabia; 334 The Liver Transplant Unit, King Faisal Specialist Hospital and Research Centre, Riyadh, Saudi Arabia; 335 The Division of Gastroenterology and Hepatology, Johns Hopkins University, Baltimore, MD USA; 336 Department of Pathology, College of Medicine, King Saud University, Riyadh, Saudi Arabia; 337 Titus Family Department of Clinical Pharmacy, USC School of Pharmacy, University of Southern California, Los Angeles, CA USA; 338 College of Applied Medical Sciences, Taibah University, Madina, Saudi Arabia; 339 Developmental Medicine Department, King Abdullah International Medical Research Center, King Saud Bin Abdulaziz University for Health Sciences, Ministry of National Guard Health Affairs, Riyadh, Saudi Arabia; 340 KACST-BWH Centre of Excellence for Biomedicine, Joint Centers of Excellence Program, King Abdulaziz City for Science and Technology (KACST), Riyadh, Saudi Arabia; 341 Ministry of the National Guard Health Affairs, King Abdullah International Medical Research Center and King Saud Bin Abdulaziz University for Health Sciences, Riyadh, Saudi Arabia; 342 Ohud Hospital, Ministry of Health, Madinah, Saudi Arabia; 343 Pediatric Infectious Diseases, Children’s Specialized Hospital, King Fahad Medical City, Riyadh, Saudi Arabia; 344 The Saudi Biobank, King Abdullah International Medical Research Center, King Saud bin Abdulaziz University for Health Sciences, Ministry of National Guard Health Affairs, Riyadh, Saudi Arabia; 345 Developmental Medicine Department, King Abdullah International Medical Research Center and King Saud Bin Abdulaziz University for Health Sciences, King Abdulaziz Medical City, Ministry of National Guard Health Affairs, Riyadh, Saudi Arabia; 346 Department of Pathology and Laboratory Medicine, King Abdulaziz Medical City, Ministry of National Guard Health Affairs, King Saud Bin Abdulaziz University for Health Sciences and King Abdullah International Medical Research Center, Riyadh, Saudi Arabia; 347 Laboratory Department, Security Forces Hospital, General Directorate of Medical Services, Ministry of Interior, Riyadh, Saudi Arabia; 348 Department of Clinical Laboratory Sciences, College of Applied Medical Sciences, King Saud University, Riyadh, Saudi Arabia; 349 King Abdulaziz City for Science and Technology (KACST), Riyadh, Saudi Arabia; 350 Life Science and Environmental Institute, King Abdulaziz City for Science and Technology (KACST), Riyadh, Saudi Arabia; 351 Department of Developmental Medicine, King Abdullah International Medical Research Center, King Saud Bin Abdulaziz University for Health Sciences, King Abdulaziz Medical City, Ministry of National Guard Health Affairs, Riyadh, Saudi Arabia; 352 Titus Family Department of Clinical Pharmacy, USC School of Pharmacy University of Southern California, Los Angeles, CA USA; 353 Centre for Fertility and Health, Norwegian Institute of Public Health, Oslo, Norway; 354 Department of Method Development and Analytics, Norwegian Institute of Public Health, Oslo, Norway; 355 Department of Genetics and Bioinformatics, Norwegian Institute of Public Health, Oslo, Norway; 356 Department of Twin Research and Genetic Epidemiology, King’s College London, London, UK; 357 NIHR Biomedical Research Centre at Guy’s and St Thomas’ Foundation Trust, London, UK; 358 Vanda Pharmaceuticals, London, UK; 359 Stroke Pharmacogenomics and Genetics, Biomedical Research Institute Sant Pau, Sant Pau Hospital, Barcelona, Spain; 360 Institute of Biomedicine of Valencia (IBV), National Spanish Research Council (CSIC), València, Spain; 361 Network Center for Biomedical Research on Neurodegenerative Diseases (CIBERNED), València, Spain; 362 Neurology and Genetic Mixed Unit, La Fe Health Research Institute, València, Spain; 363 Institute for Biomedical Research of Barcelona (IIBB), National Spanish Research Council (CSIC), Barcelona, Spain; 364 Department of Neurology, Hospital Universitari MútuaTerrassa, Fundació Docència i Recerca MútuaTerrassa, Terrassa, Spain; 365 Department of Molecular and Cell Biology, Centro Nacional de Biotecnología (CNB-CSIC), Campus Universidad Autónoma de Madrid, Madrid, Spain; 366 Instituto de Física de Cantabria (IFCA-CSIC), Santander, Spain; 367 Institut d’Investigacions Biomèdiques August Pi i Sunyer (IDIBAPS), Barcelona, Spain; 368 Hospital Clínic, Barcelona, Spain; 369 Hospital Clínic, IDIBAPS, School of Medicine, University of Barcelona, Barcelona, Spain; 370 IDIBAPS, Barcelona, Spain; 371 IIBB-CSIC, Barcelona, Spain; 372 Servicio de Salud del Principado de Asturias, Oviedo, Spain; 373 Hospital Mutua de Terrassa, Terrassa, Spain; 374 Hospital Valle Hebrón, Barcelona, Spain; 375 Instituto de Biomedicina y Genética Molecular (IBGM), CSIC-Universidad de Valladolid, Valladolid, Spain; 376 Hospital Clínico Universitario de Valladolid (SACYL), Valladolid, Spain; 377 University Hospital of Albacete, Albacete, Spain; 378 Department of Neurology, Biomedical Research Institute Sant Pau (IIB Sant Pau), Hospital de la Santa Creu i Sant Pau, Barcelona, Spain; 379 Hospital Universitario Ramon y Cajal, IRYCIS, Madrid, Spain; 380 Institute of Biomedicine of Seville (IBiS), Hospital Universitario Virgen del Rocío, CSIC and University of Seville, Seville, Spain; 381 Department of Neurology, Hospital Universitario Virgen Macarena, Seville, Spain; 382 Brigham and Women’s Hospital, Boston, MA USA; 383 Harvard Medical School, Boston, MA USA; 384 Division of Human Genetics, Department of Pediatrics, The Perelman School of Medicine, University of Pennsylvania, Philadelphia, PA USA; 385 Faculty of Medicine, University of Iceland, Reykjavik, Iceland; 386 Erasmus MC, Rotterdam, The Netherlands; 387 Finnish Institute for Health and Welfare (THL), Helsinki, Finland; 388 University of Helsinki, Faculty of Medicine, Clinical and Molecular Metabolism Research Program, Helsinki, Finland; 389 Public Health, Faculty of Medicine, University of Helsinki, Helsinki, Finland; 390 Department of Human Genetics, McGill University, Montréal, Québec Canada; 391 Lady Davis Institute, Jewish General Hospital, McGill University, Montréal, Québec Canada; 392 Kyoto–McGill International Collaborative School in Genomic Medicine, Graduate School of Medicine, Kyoto University, Kyoto, Japan; 393 Research Fellow, Japan Society for the Promotion of Science, Kyoto, Japan; 394 University of Liege, Liege, Belgium; 395 CHU of Liege, Liege, Belgium; 396 5BHUL (Liege Biobank), CHU of Liege, Liege, Belgium; 397 CHC Mont-Legia, Liege, Belgium; 398 University of Colorado Anschutz Medical Campus, Aurora, CO USA; 399 Estonian Genome Centre, Institute of Genomics, University of Tartu, Tartu, Estonia; 400 University of Tartu, Tartu, Estonia; 401 Kuressaare Hospital, Kuressaare, Estonia; 402 Tartu University Hospital, Tartu, Estonia; 403 Institute of Biomedicine and Translational Medicine, University of Tartu, Tartu, Estonia; 404 West Tallinn Central Hospital, Tallinn, Estonia; 405 Estonian Health Insurance Fund, Tallinn, Estonia; 406 Infectious Diseases Service, Department of Medicine, University Hospital and University of Lausanne, Lausanne, Switzerland; 407 Institute of Molecular and Clinical Ophthalmology Basel (IOB), Basel, Switzerland; 408 Department of Ophthalmology, University of Basel, Basel, Switzerland; 409 Centre for Primary Care and Public Health, University of Lausanne, Lausanne, Switzerland; 410 Division of Infectious Diseases and Hospital Epidemiology, Cantonal Hospital St Gallen, St Gallen, Switzerland; 411 Division of Intensive Care, Geneva University Hospitals and the University of Geneva Faculty of Medicine, Geneva, Switzerland; 412 Infectious Disease Service, Department of Internal Medicine, Geneva University Hospital, Geneva, Switzerland; 413 Clinique de Médecine et Spécialités, Infectiologie, HFR-Fribourg, Fribourg, Switzerland; 414 Infectious Diseases Division, University Hospital Centre of the canton of Vaud, Hospital of Valais, Sion, Switzerland; 415 GCAT-Genomes for Life, Germans Trias i Pujol Health Sciences Research Institute (IGTP), Badalona, Spain; 416 Catalan Institute of Oncology, Bellvitge Biomedical Research Institute, Consortium for Biomedical Research in Epidemiology and Public Health and University of Barcelona, Barcelona, Spain; 417 Massachusetts General Hospital, Boston, MA USA; 418 Life and Medical Sciences, Barcelona Supercomputing Center–Centro Nacional de Supercomputación (BSC-CNS), Barcelona, Spain; 419 ISGlobal, Barcelona, Spain; 420 IMIM (Hospital del Mar Medical Research Institute), Barcelona, Spain; 421 Universitat Pompeu Fabra (UPF), Barcelona, Spain; 422 CIBER Epidemiología y Salud Pública (CIBERESP), Madrid, Spain; 423 Medical Genetics, University of Siena, Siena, Italy; 424 Genetica Medica, Azienda Ospedaliero-Universitaria Senese, Siena, Italy; 425 Med Biotech Hub and Competence Center, Department of Medical Biotechnologies, University of Siena, Siena, Italy; 426 Infectious Diseases Clinic, Department of Medicine 2, Azienda Ospedaliera di Perugia and University of Perugia, Santa Maria Hospital, Perugia, Italy; 427 Department of Anesthesia and Intensive Care, University of Modena and Reggio Emilia, Modena, Italy; 428 Division of Infectious Diseases and Immunology, Fondazione IRCCS Policlinico San Matteo, Pavia, Italy; 429 Department of Internal Medicine and Therapeutics, University of Pavia, Pavia, Italy; 430 U.O.C. Medicina, ASST Nord Milano, Ospedale Bassini, Milan, Italy; 431 Department of Mathematics, University of Pavia, Pavia, Italy; 432 University of Siena, DIISM-SAILAB, Siena, Italy; 433 Independent researcher, Milan, Italy; 434 Fondazione IRCCS Ca’ Granda Ospedale Maggiore Policlinico, Milan, Italy; 435 Department of Infectious and Tropical Diseases, University of Brescia and ASST Spedali Civili Hospital, Brescia, Italy; 436 Department of Molecular and Translational Medicine, University of Brescia, Brescia, Italy; 437 Clinical Chemistry Laboratory, Cytogenetics and Molecular Genetics Section, Diagnostic Department, ASST Spedali Civili di Brescia, Brescia, Italy; 438 Chirurgia Vascolare, Ospedale Maggiore di Crema, Crema, Italy; 439 III Infectious Diseases Unit, ASST-FBF-Sacco, Milan, Italy; 440 Department of Biomedical and Clinical Sciences Luigi Sacco, University of Milan, Milan, Italy; 441 Department of Specialized and Internal Medicine, Tropical and Infectious Diseases Unit, Azienda Ospedaliera Universitaria Senese, Siena, Italy; 442 HIV/AIDS Department, National Institute for Infectious Diseases Lazzaro Spallanzani, IRCCS, Rome, Italy; 443 Unit of Respiratory Diseases and Lung Transplantation, Department of Internal and Specialist Medicine, University of Siena, Siena, Italy; 444 Unit of Intensive Care Medicine. Departments of Emergency and Urgency, Medicine, Surgery and Neurosciences, Siena University Hospital, Siena, Italy; 445 Unit of Diagnostic Imaging, Departments of Medical, Surgical and Neurosciences and Radiological Sciences, University of Siena, Siena, Italy; 446 Rheumatology Unit, Department of Medicine, Surgery and Neurosciences, University of Siena, Policlinico Le Scotte, Siena, Italy; 447 Infectious Diseases Unit, Department of Specialized and Internal Medicine, San Donato Hospital Arezzo, Arezzo, Italy; 448 Anesthesia Unit, Department of Emergency, San Donato Hospital, Arezzo, Italy; 449 Pneumology Unit and UTIP, Department of Specialized and Internal Medicine, San Donato Hospital, Arezzo, Italy; 450 Anesthesia Unit, Department of Emergency, Misericordia Hospital, Grosseto, Italy; 451 Infectious Diseases Unit, Department of Specialized and Internal Medicine, Misericordia Hospital, Grosseto, Italy; 452 Department of Preventive Medicine, Azienda USL Toscana Sud Est, Tuscany, Italy; 453 Clinical Chemical Analysis Laboratory, Misericordia Hospital, Grosseto, Italy; 454 Territorial Scientific Technician Department, Azienda USL Toscana Sud Est, Arezzo, Italy; 455 Clinical Chemical Analysis Laboratory, San Donato Hospital, Arezzo, Italy; 456 Department of Health Sciences, Clinic of Infectious Diseases, ASST Santi Paolo e Carlo, University of Milan, Milan, Italy; 457 Department of Medical and Surgical Sciences for Children and Adults, University of Modena and Reggio Emilia, Modena, Italy; 458 Infectious Diseases Clinic, Santa Maria Hospital, University of Perugia, Perugia, Italy; 459 Department of Infectious Diseases, Treviso Hospital, Treviso, Italy; 460 Clinical Infectious Diseases, Mestre Hospital, Venezia, Italy; 461 Infectious Diseases Clinic, Belluno, Italy; 462 Department of Molecular Medicine, University of Padova, Padua, Italy; 463 Medical Genetics and Laboratory of Medical Genetics Unit, A.O.R.N. Antonio Cardarelli Hospital, Naples, Italy; 464 Department of Molecular Medicine and Medical Biotechnology, University of Naples Federico II, Naples, Italy; 465 CEINGE Biotecnologie Avanzate, Naples, Italy; 466 IRCCS SDN, Naples, Italy; 467 Unit of Respiratory Physiopathology, AORN dei Colli, Monaldi Hospital, Naples, Italy; 468 Division of Medical Genetics, Fondazione IRCCS Casa Sollievo della Sofferenza Hospital, San Giovanni Rotondo, Italy; 469 Laboratory of Regulatory and Functional Genomics, Fondazione IRCCS Casa Sollievo della Sofferenza Hospital, San Giovanni Rotondo, Italy; 470 Department of Medical Sciences, Fondazione IRCCS Casa Sollievo della Sofferenza Hospital, San Giovanni Rotondo, Italy; 471 Clinical Trial Office, Fondazione IRCCS Casa Sollievo della Sofferenza Hospital, San Giovanni Rotondo, Italy; 472 Department of Health Sciences, University of Genova, Genova, Italy; 473 Infectious Diseases Clinic, Policlinico San Martino Hospital, IRCCS for Cancer Research, Genova, Italy; 474 Microbiology, Fondazione Policlinico Universitario Agostino Gemelli IRCCS, Catholic University of Medicine, Rome, Italy; 475 Department of Laboratory Sciences and Infectious Diseases, Fondazione Policlinico Universitario A. Gemelli IRCCS, Rome, Italy; 476 Department of Cardiovascular Diseases, University of Siena, Siena, Italy; 477 Otolaryngology Unit, University of Siena, Siena, Italy; 478 Department of Internal Medicine, ASST Valtellina e Alto Lario, Sondrio, Italy; 479 Oncologia Medica e Ufficio Flussi Sondrio, Sondrio, Italy; 480 First Aid Department, Luigi Curto Hospital, Polla, Salerno, Italy; 481 Local Health Unit, Pharmaceutical Department of Grosseto, Toscana Sud Est Local Health Unit, Grosseto, Italy; 482 U.O.C. Laboratorio di Genetica Umana, IRCCS Istituto Giannina Gaslini, Genoa, Italy; 483 Infectious Diseases Clinics, University of Modena and Reggio Emilia, Modena, Italy; 484 Department of Respiratory Diseases, Azienda Ospedaliera di Cremona, Cremona, Italy; 485 Department of Cardiovascular, Neural and Metabolic Sciences, Istituto Auxologico Italiano, IRCCS, San Luca Hospital, Milan, Italy; 486 Department of Medicine and Surgery, University of Milano-Bicocca, Milan, Italy; 487 Laboratory of Cardiovascular Genetics, Istituto Auxologico Italiano, IRCCS, Milan, Italy; 488 Unit of Infectious Diseases, ASST Papa Giovanni XXIII Hospital, Bergamo, Italy; 489 Direzione Scientifica, Istituti Clinici Scientifici Maugeri IRCCS, Pavia, Italy; 490 Department of Cardiology, Istituti Clinici Scientifici Maugeri IRCCS, Institute of Montescano, Pavia, Italy; 491 Department of Cardiac Rehabilitation, Institute of Tradate (VA) and Istituti Clinici Scientifici Maugeri IRCCS, Pavia, Italy; 492 Department of Cardiology, Istituti Clinici Scientifici Maugeri IRCCS, Institute of Milan, Milan, Italy; 493 Department of Electronics, Information and Bioengineering (DEIB), Politecnico di Milano, Milan, Italy; 494 Scuola Normale Superiore, Pisa, Italy; 495 CNR-Consiglio Nazionale delle Ricerche, Istituto di Biologia e Biotecnologia Agraria (IBBA), Milano, Italy; 496 Core Research Laboratory, ISPRO, Florence, Italy; 497 Fondazione per la Ricerca Ospedale di Bergamo, Bergamo, Italy; 498 Health Management, Azienda USL Toscana Sud Est, Tuscany, Italy; 499 IRCCS Mondino Foundation, Pavia, Italy; 500 Medical Genetics Unit, Meyer Children’s University Hospital, Florence, Italy; 501 Allelica, New York, NY USA; 502 Pneumology Unit, Department of Medicine, Misericordia Hospital, Grosseto, Italy; 503 Intensive Care Unit and Department of Anesthesia, ASST Fatebenefratelli Sacco, Luigi Sacco Hospital, Polo Universitario, University of Milan, Milan, Italy; 504 Infectious Disease Unit, Hospital of Massa, Massa, Italy; 505 Department of Clinical Medicine, Public Health, Life and Environment Sciences, University of L’Aquila, L’Aquila, Italy; 506 UOSD Laboratorio di Genetica Medica—ASL Viterbo, San Lorenzo, Italy; 507 Department of Medical Sciences, Infectious and Tropical Diseases Unit, Azienda Ospedaliera Universitaria Senese, Siena, Italy; 508 Unit of Infectious Diseases, Santa Maria Annunziata Hospital, Florence, Italy; 509 Infectious Disease Unit, Hospital of Lucca, Lucca, Italy; 510 Infectious Diseases Unit, Department of Clinical and Experimental Medicine, University of Pisa, Pisa, Italy; 511 Infectious Disease Unit, Santo Stefano Hospital, AUSL Toscana Centro, Prato, Italy; 512 Clinic of Infectious Diseases, Catholic University of the Sacred Heart, Rome, Italy; 513 Department of Diagnostic and Laboratory Medicine, Institute of Biochemistry and Clinical Biochemistry, Fondazione Policlinico Universitario A. Gemelli IRCCS, Catholic University of the Sacred Heart, Rome, Italy; 514 Department of Population Health Sciences, Geisinger Health System, Danville, PA USA; 515 Department of Molecular and Functional Genomics, Geisinger Health System, Danville, PA USA; 516 Regeneron Genetics Center, Tarrytown, NY USA; 517 Phenomic Analytics and Clinical Data Core, Geisinger Health System, Danville, PA USA; 518 Queen Mary University of London, London, UK; 519 Bradford Institute for Health Research, Bradford Teaching Hospitals National Health Service (NHS) Foundation Trust, Bradford, UK; 520 Medical and Population Genomics, Wellcome Sanger Institute, Hinxton, UK; 521 School of Basic and Medical Biosciences, Faculty of Life Sciences and Medicine, King’s College London, London, UK; 522 Department of Human Genetics, Wellcome Sanger Institute, Hinxton, UK; 523 National Institute for Health Research Blood and Transplant Research Unit in Donor Health and Genomics, University of Cambridge, Cambridge, UK; 524 Department of Haematology, University of Cambridge, Cambridge, UK; 525 British Heart Foundation Cardiovascular Epidemiology Unit, Department of Public Health and Primary Care, University of Cambridge, Cambridge, UK; 526 British Heart Foundation Centre of Research Excellence, University of Cambridge, Cambridge, UK; 527 Health Data Research UK Cambridge, Wellcome Genome Campus, University of Cambridge, Cambridge, UK; 528 Department of Genetics, University Medical Centre Groningen, University of Groningen, Groningen, The Netherlands; 529 Department of Genetics, University Medical Centre Utrecht, Utrecht, The Netherlands; 530 Department of Epidemiology, University Medical Center Groningen, University of Groningen, Groningen, The Netherlands; 531 Department of Genetics, University Medical Centre Groningen, University of Groningen, Groningen, The Netherlands; 532 Department of Psychiatry, University Medical Center Groningen, Groningen, The Netherlands; 533 Department of Biostatistics, Boston University School of Public Health, Boston, MA USA; 534 Center for Population Genomics, MAVERIC, VA Boston Healthcare System, Boston, MA USA; 535 MAVERIC, VA Boston Healthcare System, Boston, MA USA; 536 Department of Epidemiology, Emory University Rollins School of Public Health, Atlanta, GA USA; 537 Stanford University, Stanford, CA USA; 538 Vrije Universiteit Amsterdam, Amsterdam, The Netherlands; 539 Broad Institute of MIT and Harvard, Boston, MA USA; 540 Department of Genetics, University of Pennsylvania Perelman School of Medicine, Philadelphia, PA USA; 541 Department of Neuroscience, Karolinska Institutet, Stockholm, Sweden; 542 Max Planck Institute for Evolutionary Anthropology, Leipzig, Germany; 543 Anaesthesiology and Intensive Care Medicine, Department of Surgical Sciences, Uppsala University, Uppsala, Sweden; 544 Integrative Physiology, Department of Medical Cell Biology, Uppsala University, Uppsala, Sweden; 545 Stanley Center for Psychiatric Research and Program in Medical and Population Genetics, Muscatine, IA USA; 546 Division Anesthesiology and Intensive Care, CLINTEC, Karolinska Institutet, Stockholm, Sweden; 547 Department of Clinical Research and Leadership, George Washington University, Washington, DC USA; 548 Big Data Institute, Nuffield Department of Population Health, University of Oxford, Oxford, UK; 549 Wellcome Centre for Human Genetics, University of Oxford, Oxford, UK; 550 Nuffield Department of Medicine, Experimental Medicine Division, University of Oxford, John Radcliffe Hospital, Oxford, UK; 551 Public Health England, Field Service, Addenbrooke’s Hospital, Cambridge, UK; 552 Public Health England, Data and Analytical Services, National Infection Service, London, UK; 553 Genomics PLC, Oxford, UK; 554 Genomics England, London, UK; 555 Ancestry, Lehi, UT USA

**Keywords:** Respiratory distress syndrome, Genetics research, Genome-wide association studies, Infectious diseases, SARS-CoV-2

## Abstract

Critical COVID-19 is caused by immune-mediated inflammatory lung injury. Host genetic variation influences the development of illness requiring critical care^1^ or hospitalization^2–4^ after infection with SARS-CoV-2. The GenOMICC (Genetics of Mortality in Critical Care) study enables the comparison of genomes from individuals who are critically ill with those of population controls to find underlying disease mechanisms. Here we use whole-genome sequencing in 7,491 critically ill individuals compared with 48,400 controls to discover and replicate 23 independent variants that significantly predispose to critical COVID-19. We identify 16 new independent associations, including variants within genes that are involved in interferon signalling (*IL10RB* and *PLSCR1*), leucocyte differentiation (*BCL11A*) and blood-type antigen secretor status (*FUT2*). Using transcriptome-wide association and colocalization to infer the effect of gene expression on disease severity, we find evidence that implicates multiple genes—including reduced expression of a membrane flippase (*ATP11A*), and increased expression of a mucin (*MUC1*)—in critical disease. Mendelian randomization provides evidence in support of causal roles for myeloid cell adhesion molecules (*SELE*, *ICAM5* and *CD209*) and the coagulation factor *F8*, all of which are potentially druggable targets. Our results are broadly consistent with a multi-component model of COVID-19 pathophysiology, in which at least two distinct mechanisms can predispose to life-threatening disease: failure to control viral replication; or an enhanced tendency towards pulmonary inflammation and intravascular coagulation. We show that comparison between cases of critical illness and population controls is highly efficient for the detection of therapeutically relevant mechanisms of disease.

## Abstract (web summary)

Whole-genome sequencing, transcriptome-wide association and fine-mapping analyses in over 7,000 individuals with critical COVID-19 are used to identify 16 independent variants that are associated with severe illness in COVID-19.

## Main

Critical illness in COVID-19 is both an extreme disease phenotype and a relatively homogeneous clinical definition; it includes patients with hypoxaemic respiratory failure^5^ with acute lung injury^6^, and excludes many patients with non-pulmonary clinical presentations^7^, who are known to have divergent responses to therapy^8^. In the UK, individuals in the critically ill group are younger, less likely to have significant comorbidity and more severely affected than a general hospitalized cohort^5^, characteristics which may amplify observed genetic effects. In addition, as development of critical illness is in itself a key clinical end-point for therapeutic trials^8^, using critical illness as a phenotype in genetic studies enables the detection of directly therapeutically relevant genetic effects^1^.

Using microarray genotyping in 2,244 cases, we previously discovered that critical COVID-19 is associated with genetic variation in the host immune response to viral infection (*OAS1*, *IFNAR2* and *TYK2*) and the inflammasome regulator *DPP9*^1^. In collaboration with international groups, we extended these findings to include a variant near *TAC4* (rs77534576)^3^. Several variants have been associated with milder phenotypes, including the ABO blood-type locus^2^, a pleiotropic inversion in chr17q21.31^9^ and associations in five additional loci, including the T lymphocyte-associated transcription factor, *FOXP4*^3^. An enrichment of rare loss-of-function variants in candidate interferon signalling genes has been reported^4^, but this has yet to be replicated at genome-wide significance thresholds^10,11^.

**Extended Data Fig. 1:**
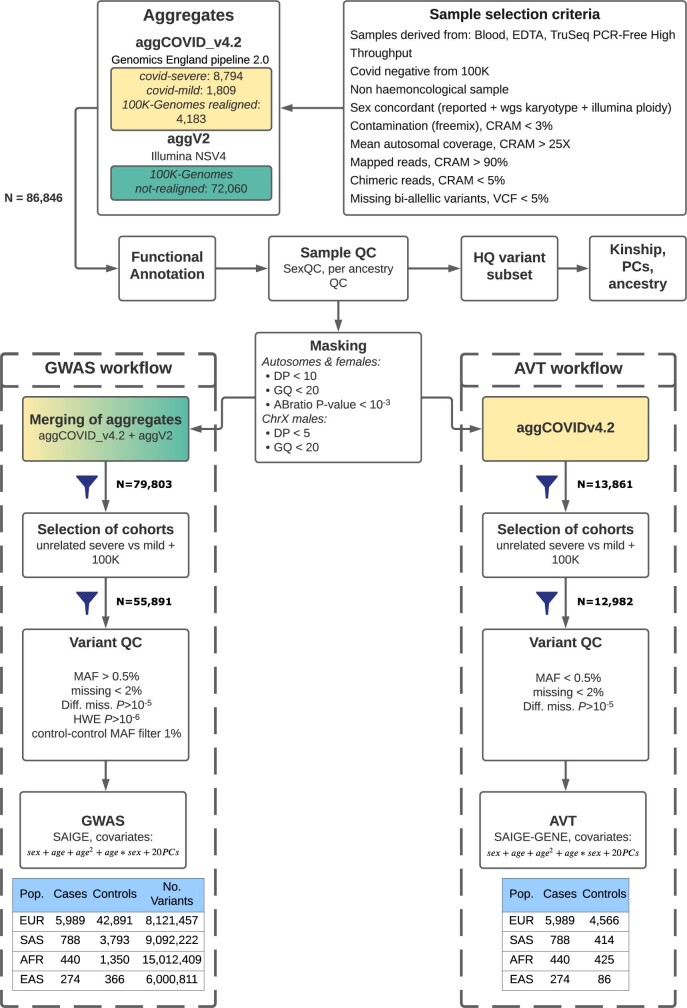
Analysis workflow for GWAS and AVT analyses of this study. The cohorts displayed in yellow and green in the top box were processed with Genomics England Pipeline 2.0 and Illumina NSV4, respectively (see Methods on WGS Alignment and variant calling for details on differences between pipelines). We used individuals that were processed with either pipeline for the GWAS analyses and individuals processed only with Genomics England Pipeline 2.0 for the AVT analyses. The definition of the cases and controls was the same for GWAS and AVT, cases were the COVID-19 severe individuals for both, and controls included individuals from the 100,000 Genomes Project (100,000 Genomes Project) and also COVID-19 positive individuals that were recruited for this study and experienced only mild symptoms (COVID-mild).

In partnership with Genomics England, we performed whole-genome sequencing (WGS) to improve the resolution and deepen the fine-mapping of significant signals and thereby provide further biological insight into critical COVID-19. Here we present results from a cohort of 7,491 critically ill patients from 224 intensive care units, compared with 48,400 control individuals, describing the discovery and validation of 23 gene loci for susceptibility to critical COVID-19 (Extended Data Fig. 1).

## Genome-wide association study analysis

After quality control procedures, we used a logistic mixed model regression, implemented in SAIGE^12^, to perform association analyses with unrelated individuals (critically ill cases, *n* = 7,491; controls, *n* = 48,400 (100,000 Genomes Project (100k) cohort, *n* = 46,770; mild COVID-19, *n* = 1,630) (Methods, Supplementary Table 2). A total of 1,339 of these cases were included in the primary analysis for our previous report^1^. Genome-wide association studies (GWASs) were performed separately for genetic ancestry groups (*n*_cases_/*n*_controls_: European (EUR) 5,989/42,891; South Asian (SAS) 788/3,793; African (AFR) 440/1,350; East Asian (EAS) 274/366), and combined by inverse-variance-weighted fixed effects meta-analysis using METAL (Methods). We established the independence of signals using GCTA-cojo, and we validated this with conditional analysis using individual-level data with SAIGE (Methods, Supplementary Table 6). To reduce the risk of spurious associations arising from genotyping or pipeline errors, we required supporting evidence from variants in linkage disequilibrium (LD) for all genome-wide-significant variants: observed *z*-scores for each variant were compared with imputed *z*-scores for the same variant, with discrepant values being excluded (see Methods, Supplementary Fig. 2).

**Fig. 1:**
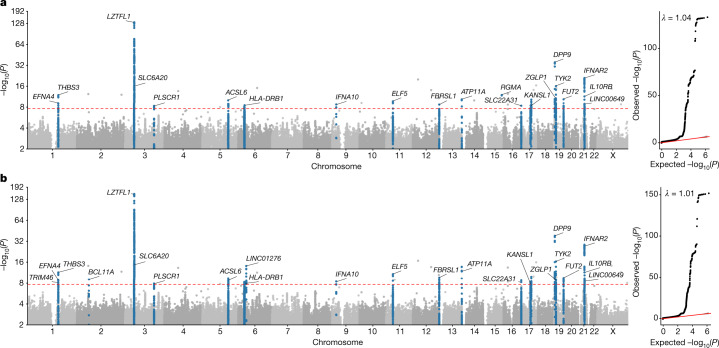
GWAS results for the EUR ancestry group, and multi-ancestry meta-analysis. Manhattan plots are shown on the left and quantile–quantile (QQ) plots of observed versus expected $P$ values on the right, with genomic inflation (*λ*) displayed for each analysis. Highlighted results in blue in the Manhattan plots indicate variants that are LD-clumped (*r*^2^ = 0.1, *P*_2_ = 0.01, EUR LD) with the lead variants at each locus. Gene name annotation indicates genes that are affected by the predicted worst consequence type of each lead variant (annotation by Variant Effect Predictor (VEP)). For the HLA locus, the gene that was identified by HLA allele analysis is annotated. The GWAS was performed using logistic regression and meta-analysed by the inverse variant method. The red dashed line shows the Bonferroni-corrected *P* value: *P* = 2.2 × 10^−8^.

In population-specific analyses, we discovered 22 independent genome-wide-significant associations in the EUR ancestry group (Fig. 1, Supplementary Fig. 11, Table 1) at a $P$ value threshold adjusted for multiple testing (2.2 × 10^−08^; Supplementary Table 5). In multi-ancestry meta-analysis, we identified an additional three independent genome-wide-significant association signals (Fig. 1, Table 1).

**Table 1:** Lead variants from independent association signals in the per-population GWAS and multi-ancestry meta-analysis

chr:pos (hg38)	rsID	REF	ALT	RAF	OR	OR_CI_	*P*	*P*_hgib2.23m_	*P*_reg_	Consequence	Gene	Cit.
1:155066988	rs114301457	C	T*	0.0058	2.4	1.82–3.16	6.8$\times {10}^{-10}$	0.00011*	−	Synonymous	*EFNA4*	−
1:155175305^‡^	rs7528026	G	A*	0.032	1.4	1.24–1.55	7.16$\times {10}^{-9}$	0.00012*	−	Intron	*TRIM46*	−
1:155197995	rs41264915	A*	G	0.89	1.3	1.19–1.37	1.02$\times {10}^{-12}$	1.51$\times {10}^{-9}$*	−	Intron	*THBS3*	^3^
2:60480453^‡^	rs1123573	A*	G	0.61	1.1	1.09–1.18	9.85$\times {10}^{-10}$	0.000018*	−	Intron	*BCL11A*	−
3:45796521	rs2271616	G	T*	0.14	1.3	1.21–1.37	9.9$\times {10}^{-17}$	4.95$\times {10}^{-9}$*	−	5′ UTR	*SLC6A20*	^3^
3:45859597	rs73064425	C	T*	0.077	2.7	2.51–2.94	1.97$\times {10}^{-133}$	1.02$\times {10}^{-77}$*	−	Intron	*LZTFL1*	^2^
3:146517122	rs343320	G	A*	0.081	1.2	1.16–1.35	4.94$\times {10}^{-9}$	0.00028*	−	Missense	*PLSCR1*	−
5:131995059	rs56162149	C	T*	0.17	1.2	1.13–1.26	7.65$\times {10}^{-11}$	0.00074*	−	Intron	*ACSL6*	−
6:32623820	rs9271609	T*	C	0.65	1.1	1.09–1.19	3.26$\times {10}^{-9}$	0.89	−	−	*HLA-DRB1*	−
6:41515007^‡^	rs2496644	A*	C	0.015	1.4	1.32–1.60	7.59$\times {10}^{-15}$	3.17$\times {10}^{-7}$*	−	Intron	*LINC01276*	^3^
9:21206606	rs28368148	C	G*	0.013	1.7	1.45–2.09	1.93$\times {10}^{-9}$	0.0024	0.00089	Missense	*IFNA10*	−
11:34482745	rs61882275	G*	A	0.62	1.1	1.10–1.20	1.61$\times {10}^{-10}$	1.9$\times {10}^{-10}$*	−	Intron	*ELF5*	−
12:132489230	rs56106917	GC	G*	0.49	1.1	1.09–1.18	2.08$\times {10}^{-9}$	0.00047*	−	Upstream	*FBRSL1*	−
13:112889041	rs9577175	C	T*	0.23	1.2	1.12–1.24	3.71$\times {10}^{-11}$	1.29$\times {10}^{-6}$*	−	Downstream	*ATP11A*	−
15:93046840^‡^	rs4424872	T*	A	0.0079	2.4	1.87–3.01	8.61$\times {10}^{-13}$	−	0.29	Intron	*RGMA*	−
16:89196249	rs117169628	G	A*	0.15	1.2	1.12–1.26	4.4$\times {10}^{-9}$	6.57$\times {10}^{-9}$*	−	Missense	*SLC22A31*	
17:46152620	rs2532300	T*	C	0.77	1.2	1.10–1.22	4.19$\times {10}^{-9}$	2.49$\times {10}^{-9}$*	−	Intron	*KANSL1*	^9^
17:49863260	rs3848456	C	A*	0.029	1.5	1.33–1.70	4.19$\times {10}^{-11}$	1.34$\times {10}^{-7}$*	−	Regulatory	.	^3^
19:4717660	rs12610495	A	G*	0.31	1.3	1.27–1.38	3.91$\times {10}^{-36}$	5.74$\times {10}^{-19}$*	−	Intron	*DPP9*	^1^
19:10305768	rs73510898	G	A*	0.093	1.3	1.19–1.37	1.57$\times {10}^{-11}$	0.00016*	−	Intron	*ZGLP1*	−
19:10352442	rs34536443	G	C*	0.05	1.5	1.36–1.65	6.98$\times {10}^{-17}$	4.06$\times {10}^{-11}$*	−	Missense	*TYK2*	^1^
19:48697960	rs368565	C	T*	0.44	1.1	1.1–1.2	3.55$\times {10}^{-11}$	0.00087*	−	Intron	*FUT2*	−
21:33230000	rs17860115	C	A*	0.32	1.2	1.19–1.3	9.69$\times {10}^{-22}$	1.77$\times {10}^{-18}$*	−	5′ UTR	*IFNAR2*	^1^
21:33287378	rs8178521	C	T*	0.27	1.2	1.12–1.23	3.53$\times {10}^{-12}$	8.02$\times {10}^{-6}$*	−	Intron	*IL10RB*	−
21:33959662	rs35370143	T	TAC*	0.083	1.3	1.17–1.36	1.24$\times {10}^{-9}$	2.33$\times {10}^{-7}$*	−	Intron	*LINC00649*	−

Variants and the reference and alternative allele are reported according to GRCh38. The three variants discovered in multi-ancestry meta-analysis but not in the European ancestry GWAS are labelled with ‡, and † indicates genome-wide significant heterogeneity. REF and ALT columns indicate the reference and alternative alleles; an asterisk (*) indicates the risk allele. For each variant, we report the risk allele frequency in Europeans (RAF), the odds ratio and 95% confidence interval (OR and OR_CI_), and the association *P* value. ‘Consequence’ indicates the predicted worst consequence type across GENCODE basic transcripts predicted by VEP (v.104), and ‘Gene’ indicates the VEP-predicted gene, but not necessarily the causal mediator. For the HLA locus, the gene that was identified by HLA allele analysis is displayed. An asterisk (*) next to the replication *P* value (*P*_hgib2.23m_ - HGI B2 and 23andMe; or *P*_reg_- Regeneron) indicates that the lead signal (from multi-ancestry meta-analysis) is replicated with a Bonferroni-corrected *P* < 0.002 (0.05/25) with a concordant direction of effect. The ‘Cit.’ column lists citation numbers for the first publication of confirmed genome-wide associations with critical illness or (in brackets) any COVID-19 phenotype.

To assess the sensitivity of our results to mismatches of demographic characteristics between cases and controls (Supplementary Figs. 9, 10), we performed an age-, sex- and body mass index (BMI)-matched case–control analysis (Supplementary Figs. 18–21). As there is a theoretical risk of mismatch between cases and 100,000 Genomes Project participants in risk factors for exposure (for example, shielding behaviour) or susceptibility to critical COVID-19 (for example, immunosuppression), we performed a sensitivity analysis using only the cohort with mild COVID-19 (see above; Supplementary Table 10). In both of these analyses, allele frequencies and directions of effect were concordant for all lead signals.

**Extended Data Fig. 2:**
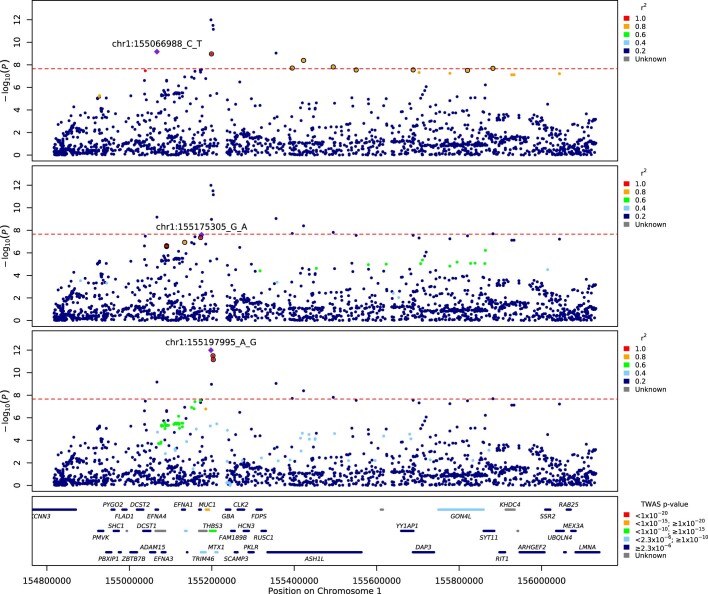
Regional detail showing fine-mapping to identify three adjacent independent signals on chromosome 1. Top two panels: variants in LD with the lead variants shown. The variants that are included in two independent credible sets are displayed with black outline circles. ${r}^{2}$ values in the legend denote upper limits, 0.2=[0,0.2], 0.4=[0.2,0.4], 0.6=[0.4,0.6], 0.8=[0.6,0.8],1=[0.8,1]. Bottom panel: locations of protein-coding genes, coloured by TWAS $P$-value. The red dashed line shows the Bonferroni-corrected $P$-value=$2.2\times {10}^{-8}$ for Europeans.

**Extended Data Fig. 4:**
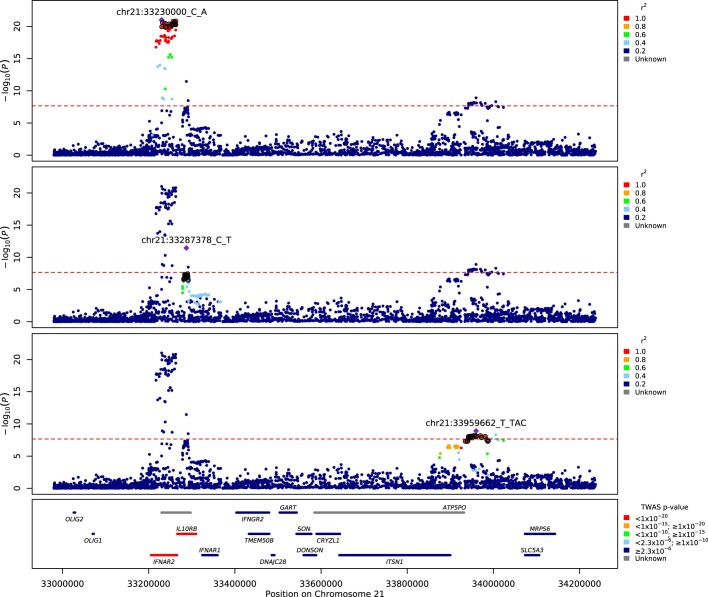
Regional detail showing fine-mapping to identify three adjacent independent signals on chromosome 21. Top three panels: variants in LD with the lead variants shown. The variants that are included in three independent credible sets are displayed with black outline circles. ${r}^{2}$ values in the legend denote upper limits, 0.2=[0,0.2], 0.4=[0.2,0.4], 0.6=[0.4,0.6], 0.8=[0.6,0.8],1=[0.8,1]. Bottom panel: locations of protein-coding genes, coloured by TWAS $P$-value. The red dashed line shows the Bonferroni-corrected $P$-value=$2.2\times {10}^{-8}$ for Europeans.

**Extended Data Fig. 5:**
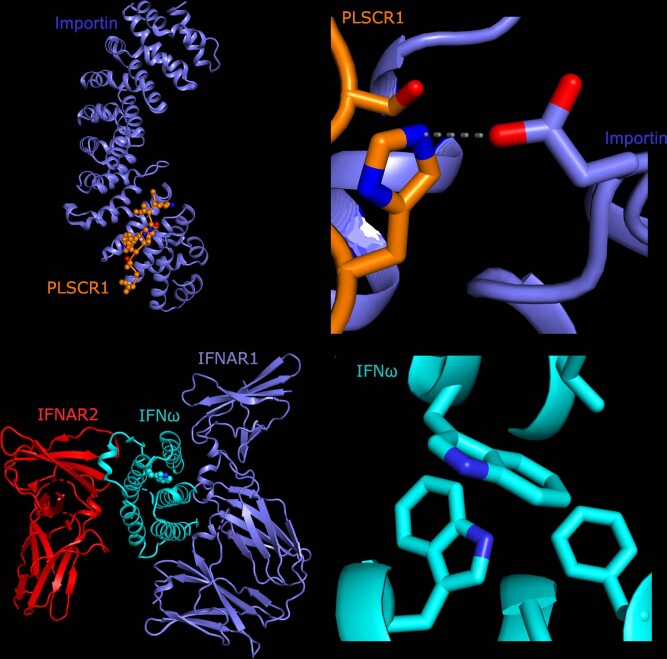
Predicted structural consequences of lead variants at PLSCR1 and IFNA10. (**a**) Crystal structure of PLSCR1 nuclear localization signal (orange, Gly257–Ile266, numbering correspond to UniProt entry O15162) in complex with Importin $\alpha$ (blue), Protein Data Bank (PDB) ID 1Y2A (ref. ^65^). Side chains of PLSCR1 are shown as connected spheres with carbon atoms coloured in orange, nitrogens in blue and oxygens in red. Hydrogen atoms were not determined at this resolution (2.20) and are not shown. (**b**) Close-up view showing side chains of PLSCR1 Ser260, His262 and Importin Glu107 as sticks. Distance (in) between selected atoms (PLSCR1 His262 $N\epsilon 2$ and Importin Glu107 carboxyl O) is indicated. A hydrogen bond between PLSCR1 His262 and Importin Glu107 is indicated with a dashed line. The risk variant is predicted to eliminate this bond, disrupting nuclear import, an essential step for effect on antiviral signalling^27^ and neutrophil maturation^66^. (**c**) Because there is very strong sequence conservation between IFNA10 and the gene encoding IFN$\omega$, we used existing crystal structure data (Protein Data Bank ID 3SE4 (ref. ^67^)) for IFN$\omega$ (cyan) to display a ternary complex with interferon α/β receptor IFNAR1 (blue), IFNAR2 (red). The side chain of Trp164 is shown as spheres and indicated with a black line. (**d**) The hydrophobic core of IFN$\omega$ with Trp164 shielded from the solvent in the center. Trp164-surrounding residues of IFN$\omega$ are numbered and correspond to UniProt entry P05000. Trp164 and surrounding residues are conserved in IFNA10 (UniProt ID P01566) and share the same numbering as in IFN$\omega$ (P05000). Side chains of four residues are shown as sticks. Carbon and nitrogen atoms coloured in cyan and blue, respectively. The critical COVID-19-associated mutation, Trp164Cys, would replace an evolutionarily conserved, bulky side chain in the hydrophobic core of IFNA10 with a smaller one, which may destabilize IFNA10.

We inferred credible sets of variants using Bayesian fine-mapping with susieR^13^, by analysing the GWAS summaries of 17 regions of genomic length 3 Mb that were flanking groups of lead signals. We obtained 22 independent credible sets of variants for EUR and an additional 2 from the trans-ancestry meta-analysis with a posterior inclusion probability greater than 0.95 (Extended Data Table 1, Supplementary Information). Fine-mapping of the association signals revealed putative causal variants for both previously reported and novel association signals (see Supplementary Information, Extended Data Table 1). In 12 out of the 24 fine-mapped signals, the credible sets included 5 or fewer variants, and for 8 signals we detected variants with predicted missense or worse consequence across each credible set (Extended Data Table 1). We were able to fine-map multiple independent signals at previously identified loci (Fig. 3, Extended Data Figs. 2, 4). For example, the signal in the 3p21.31 region^2^, was fine-mapped into two independent associations, with the credible set for the first refined to a single variant in the 5′ untranslated region (UTR) of *SLC6A20* (chr3:45796521:G:T, rs2271616, odds ratio (OR): 1.29, 95% confidence interval (CI):1.21–1.37), and the second credible set including multiple variants in downstream and intronic regions of *LZTFL1* (Fig. 3). Among the novel signals, at 3q24 and 9p21.3 we detected missense variants that affect *PLSCR1* and *IFNA10*, respectively (chr3:146517122:G:A, rs343320, p.His262Tyr, OR: 1.24, 95% CI: 1.15–1.33, CADD: 22.6; chr9:21206606:C:G, rs28368148, p.Trp164Cys, OR:1.74, 95% CI: 1.45–2.09, CADD: 23.9). Both are predicted to be deleterious by the Combined Annotation Dependent Depletion (CADD) tool^14^. Structural predictions for these variants suggest functional effects (Extended Data Fig. 5). We assessed whether the main signals of this study were underlain by rarer variants with a lower minor allele frequency (MAF) (less than 0.02%) than our GWAS default threshold (less than 0.5%), by including rarer variant summaries when fine-mapping, but no additional variants were added to the main credible sets (Supplementary Table 9).

Consistent with our expectation that genetic susceptibility has a stronger role in younger individuals, age-stratified analysis (individuals of younger than 60 years old versus individuals of 60 years old or above) in the EUR group revealed a signal in the 3p21.31 region with a significantly stronger effect in the younger age group (chr3:45801750:G:A, rs13071258, OR: 3.34, 95% CI: 2.98–3.75 versus OR: 2.1, 95% CI 1.88–2.34), which is in strong LD (*r*^2^ = 0.947) with the main GWAS signal indexed by rs73064425. Sex-specific analysis did not reveal significant effects (Supplementary Fig. 17).

## Replication

For replication, we performed a meta-analysis of summary statistics generously shared by 23andMe and the COVID-19 Host Genetics Initiative (HGI) data freeze 6 (B2). As a previous analysis of GenOMICC^1^ contributes a substantial part of the signal at each locus in HGI v.6, and leave-one-out analyses were not available, we removed the signal from GenOMICC cases in HGI v.6 using mathematical subtraction to ensure independence (Methods). Using LD clumping to find variants genotyped in both the discovery and replication studies, we required *P* < 0.002 (0.05/25) and concordant direction of effect (Table 1, Supplementary Table 8) for replication. We interrogated two variants that failed replication in this set in a second GWAS meta-analysis of hospitalized patients with COVID-19 from UK Biobank, AncestryDNA, Penn Medicine Biobank and Geisinger Health Systems, which included a total of 9,937 individuals who were hospitalized with COVID-19 and 1,059,390 control individuals. This led to a further successful replicated finding, in *IFNA10* (Table 1).

We replicated 23 of the 25 significant associations that were identified in the population-specific and/or multi-ancestry GWASs. One of the non-replicated signals (rs4424872) corresponds to a rare variant that may not be well represented in the replication datasets—which are dominated by single-nucleotide polymorphism (SNP) genotyping data—but which also had significant heterogeneity among ancestries. The second non-replicated signal is within the human leukocyte antigen (HLA) locus, which has complex LD (see below).

## HLA region

**Extended Data Fig. 6:**
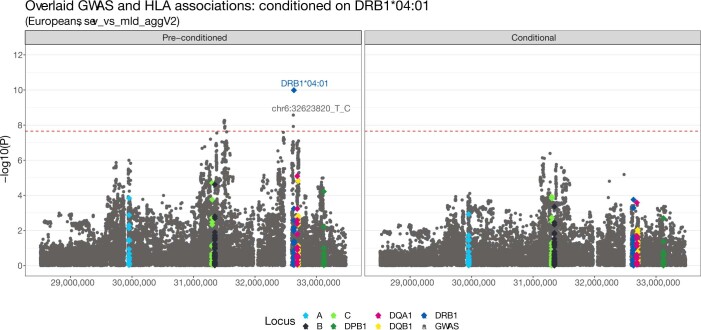
Manhattan plot of HLA and GWAS signal across the extended MHC region for the EUR cohort. Grey circles mark the GWAS (small variant) associations and diamonds represent the HLA each allele association, coloured by locus. The lead variant from the GWAS and lead allele from HLA are labelled. The left-panel shows the raw association −log_10_(*P* values) per variant - prior to conditional analysis. The right-panel shows the −log_10_(*P* values) per variant following conditioning on DRB1*04:01. The dashed red line shows the Bonferroni-corrected genome-wide significance threshold for Europeans.

The lead variant in the HLA region, rs9271609, lies upstream of the *HLA-DQA1* and *HLA-DRB1* genes. To investigate the contribution of specific HLA alleles to the observed association in the HLA region, we imputed HLA alleles at a four-digit (two-field) level using HIBAG^15^. The only allele that reached genome-wide significance was HLA-DRB1*04:01 (OR: 0.80, 95% CI: 0.75–0.86, *P* = 1.6 × 10^−10^ in EUR), which has a stronger $P$ value than the lead SNP in the region (OR: 0.88, 95% CI: 0.84–0.92, *P* = 3.3 × 10^−9^ in EUR) and is a better fit to the data (Akaike information criterion (AIC): AIC_DRB1*04:01_ = 30,241.34; AIC_leadSNP_ = 30,252.93) (Extended Data Fig. 6). HLA-DRB1*04:01 has been previously reported to confer protection against severe disease in a small cohort of European ancestry^16^.

## Gene burden testing

To assess the contribution of rare variants to critical illness, we performed gene-based analysis using SKAT-O as implemented in SAIGE-GENE^17^ on a subset of 12,982 individuals from our cohort (7,491 individuals with critical COVID-19 and 5,391 control individuals), for which the genome-sequencing data were processed with the same alignment and variant calling pipeline. We tested the burden of rare (MAF < 0.5%) variants considering the predicted variant consequence type (tested variant counts provided in the Supplementary Information). We assessed burden using a strict definition for damaging variants (high-confidence putative loss-of-function (pLoF) variants as identified by LOFTEE^18^) and a lenient definition (pLoF plus missense variants with CADD ≥ 10)^14^, but found no significant associations at a gene-wide-significance level. Moreover, all individual rare variants included in the tests had *P* values greater than 10^−5^.

Consistent with other recent work^11^, we did not find any significant gene burden test associations among the 13 genes previously reported from an interferon-pathway-focused study^4^ (tests for all genes had *P* > 0.05; Supplementary Information), and we did not replicate the reported association^19–21^ in *TLR7* (EUR *P* = 0.30 for pLoF and *P* = 0.075 for missense variants).

## Transcriptome-wide association study analysis

**Fig. 2:**
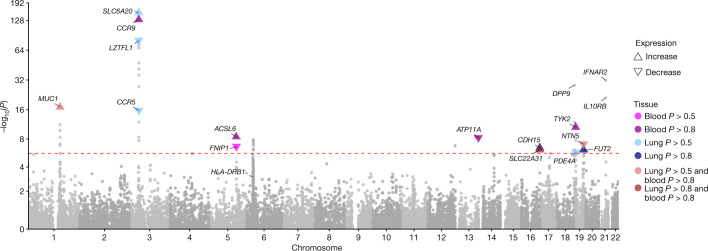
Gene-level Manhattan plot showing results from the TWAS meta-analysis and highlighting genes that colocalize with GWAS signals or have strong metaTWAS associations. The highlighting colour is different for the lung and blood tissue data that were used for colocalization, and we also distinguish loci that were significant in both. Results are grouped according to two classes for the posterior probability of colocalization (PP_H4_): *P* > 0.5 and *P* > 0.8. If a variant is placed in both classes, then the colour that corresponds to the higher probability class is shown. Arrowheads indicate the direction of change in gene expression associated with an increased disease risk. The red dashed line shows the Bonferroni-corrected significance threshold for the metaTWAS analysis at *P* = 2.3 × 10^−6^.

To infer the effect of genetically determined variation in gene expression on disease susceptibility, we performed a transcriptome-wide association study (TWAS) using gene expression data (GTEx v.8; ref. ^22^) for two disease-relevant tissues: lung and whole blood. We found significant associations between critical COVID-19 and predicted expression in lung (14 genes) and blood (6 genes) (Supplementary Fig. 23) and in an all-tissue meta-analysis (GTEx v.8; 51 genes) (Supplementary Fig. 24). Expression signals for 16 genes significantly colocalized with susceptibility (Fig. 2). As the LD structure of the HLA is complex, we only assessed colocalization for the significant association, *HLA-DRB1*. Although it was not significant in our TWAS analysis, expression quantitative trait loci (eQTLs) in the proximity of the association significantly colocalize with the GWAS signal for both blood and lung (both PP_H4_ > 0.8; Supplementary Information).

We repeated the TWAS analysis using models of intron excision rate from GTEx v.8 to obtain a splicing TWAS, which revealed significant signals in lung (16 genes) and whole blood (9 genes), and in an all-tissue meta-analysis (33 genes); 11 of these had strongly colocalizing splicing signals (Supplementary Information).

## Mendelian randomization

**Extended Data Fig. 7:**
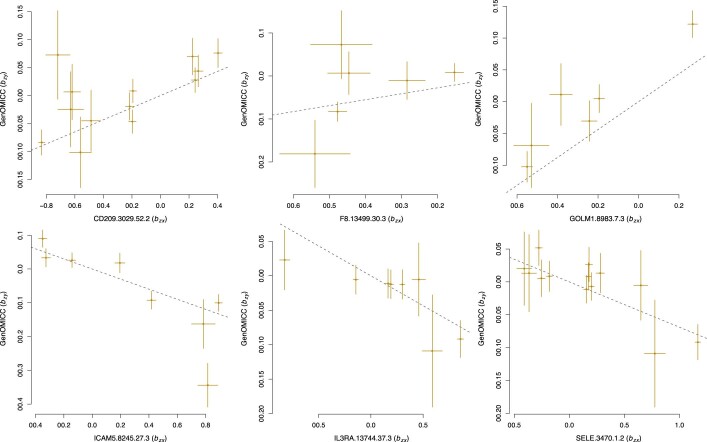
Effect–effect plots for Mendelian randomization analyses to assess causal evidence for circulating proteins in critical COVID-19. Each plot shows effect size (β) of variants associated with protein concentration (*x* axis) and critical COVID-19 (*y* axis). A full list of instruments is found in Supplementary Table 13.

We performed generalized summary-data-based Mendelian randomization (GSMR)^23^ in a replicated outcome study design using the protein quantitative trait loci (pQTLs) from the INTERVAL study^24^. GSMR incorporates information from multiple independent SNPs and provides stronger evidence of a causal relationship than single-SNP-based approaches. Of 16 proteome-wide-significant associations in this study, 8 were replicated in an external dataset at a Bonferroni-corrected *P* value threshold of *P* < 0.0031 (*P* < 0.05/16; Extended Data Table 2, Extended Data Fig. 7) .

**Fig. 3:**
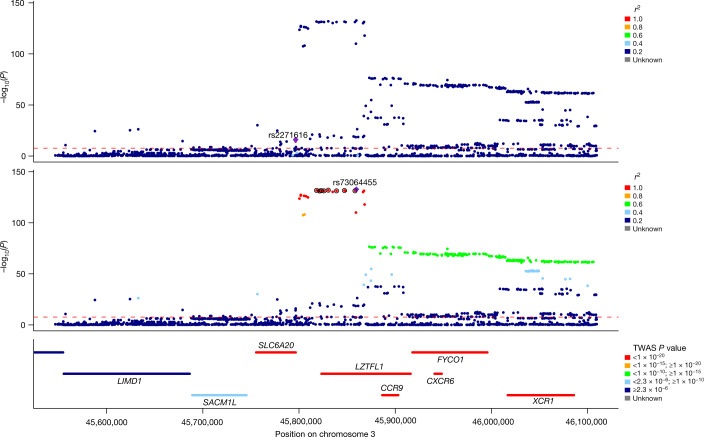
Regional detail showing fine-mapping to identify two adjacent independent signals on chromosome 3. Top two panels, variants in LD with the lead variants shown. The variants that are included in two independent credible sets are displayed with black outline circles. The *r*^2^ values in the key denote upper limits; that is, 0.2 = [0, 0.2], 0.4 = [0.2, 0.4], 0.6 = [0.4, 0.6], 0.8 = [0.6, 0.8],1 = [0.8, 1]. Bottom, locations of protein-coding genes, coloured by TWAS *P* value. The red dashed line shows the Bonferroni-corrected *P* value: *P* = 2.2 × 10^−8^ for individuals of European ancestry.

## Discussion

We report 23 replicated genetic associations with critical COVID-19, which were discovered in only 7,491 cases. This demonstrates the efficiency of the design of the GenOMICC study, an open-source^25^ international research programme (https://genomicc.org) that focuses on extreme phenotypes: patients with life-threatening infectious disease, sepsis, pancreatitis and other critical illness phenotypes. GenOMICC detects greater heritability and stronger effect sizes than other study designs across all variants (Supplementary Figs. 22, 14). In COVID-19, critical illness is not only an extreme susceptibility phenotype, but also a more homogeneous one: we have shown previously that critically ill patients with COVID-19 are more likely to have the primary disease process—hypoxaemic respiratory failure^5^—and that patients in this group have a divergent response to immunosuppressive therapy compared to other hospitalized patients^8^. We detect distinct signals at several of the associated loci, in some cases implicating different biological mechanisms.

Five of the variants associated with critical COVID-19 have direct roles in interferon signalling and broadly concordant predicted biological effects. These include a probable destabilizing amino acid substitution in a ligand, *IFNA10* (Trp164Cys, Extended Data Fig. 5), and—as we reported previously^1^—reduced expression of a subunit of its receptor *IFNAR2* (Fig. 2). *IFNAR2* signals through a kinase that is encoded by *TYK2*^1^. Although the lead variant in *TYK2* in WGS is a protein-coding variant with reduced STAT1 phosphorylation activity^26^, it is also associated with significantly increased expression of *TYK2* (Fig. 2, Methods). Fine-mapping reveals a significant association with an independent missense variant in *IL10RB*, a receptor for type III (lambda) interferons (rs8178521; Table 1). Finally, we detected a lead risk variant in phospholipid scramblase 1 (chr3:146517122:G:A, rs343320; *PLSCR1*) which disrupts a nuclear localization signal that is important for the antiviral effect of interferons^27^ (Extended Data Fig. 5). PLSCR1 controls the replication of other RNA viruses, including vesicular stomatitis virus, encephalomyocarditis virus and influenza A virus^27,28^.

Although our genome-wide gene-based association tests did not replicate any findings from a previous pathway-specific study of rare deleterious variants^4^, our results provide robust evidence implicating reduced interferon signalling in susceptibility to critical COVID-19. Notably, systemic administration of interferon in two large clinical trials, albeit late in disease, did not reduce mortality^29,30^.

We found significant associations in genes that are implicated in lymphopoesis and in the differentiation of myeloid cells. *BCL11A* is essential for B and T lymphopoiesis^31^ and promotes the differentiation of plasmacytoid dendritic cells^32^. *TAC4*, reported previously^3^, encodes a regulator of B cell lymphopoesis^33^ and antibody production^34^, and promotes the survival of dendritic cells^35^. Finally, although the strongest fine-mapping signal at 5q31.1 (chr5:131995059:C:T, rs56162149) is in an intron of *ACSL6* with significant effects on expression (Supplementary Information), the credible set includes a missense variant in *CSF2* (encoding granulocyte–macrophage colony stimulating factor; GM-CSF) of uncertain significance (chr5:132075767:T:C; Extended Data Table 1). We have previously shown that GM-CSF is strongly up-regulated in critical COVID-19^36^, and it is already under investigation as a target for therapy^37^. Mendelian randomization results are consistent with a direct link between the plasma levels of a closely related cytokine receptor subunit, IL3RA, and critical COVID-19 (Extended Data Table 2).

Fine-mapping, colocalization and TWAS analyses provide evidence for increased expression of *MUC1* as the mediator of the association with rs41264915 (Supplementary Table 12). This suggests that mucins could have a therapeutically important role in the development of critical illness in COVID-19.

Mendelian randomization provides genetic evidence in support of a causal role for coagulation factors (*F8*) and platelet activation (*PDGFRL*) in critical COVID-19 (Extended Data Table 2, Extended Data Fig. 7), consistent with autopsy^6^, proteomic^38^ and therapeutic^39^ evidence. Perhaps more importantly, we identify specific and closely related intercellular adhesion molecules that have known roles in the recruitment of inflammatory cells to sites of inflammation, including E-selectin (*SELE*), intercellular adhesion molecule 5 (*ICAM5*) and DC-SIGN (dendritic-cell-specific ICAM3-grabbing non-integrin; *CD209*), which may provide additional therapeutic targets. DC-SIGN (*CD209*) mediates pathogen endocytosis and antigen presentation, and is known to be involved in multiple viral infections, including SARS-CoV and influenza A virus. It has affinity for SARS-CoV-2^40,41^.

Our previous report of an association between the OAS gene cluster and severe disease was robustly replicated in an external cohort^1^, but does not meet genome-wide significance in the present analysis (Supplementary Table 7). This may indicate a change in the observed effect size because any effect that is detected in GWASs is more likely to have been sampled from the larger end of the range of possible effect sizes —the ‘winner’s curse’. Alternatively, it may indicate either a change in the population of patients (cases or controls) or a change in the pathogen. For example it is possible that—as with the other coronaviruses that are known to infect humans^42^—more recent variants of SARS-CoV-2 have evolved to overcome this host antiviral defence mechanism.

## Limitations

In contrast to microarray genotyping, WGS is a rapidly evolving and relatively new technology for GWASs, with relatively few sources of population controls. We selected a control cohort from the 100,000 Genomes Project, which was sequenced and analysed using a different platform and bioinformatics pipeline compared with the case cohort (Extended Data Fig. 1). However, to minimize the risk of false-positive associations due to technical artifacts, extensive quality measures were used (Methods). In brief, we masked low-quality genotypes, filtered for genotype signal using a low threshold for missingness and performed a control–control relative allele frequency filter using a subset of samples processed with both bioinformatics pipelines. Finally, we required all significant associations to be supported by local variants in LD, which may be excessively stringent (Methods). Although this approach may remove some true associations, our priority is to maximize confidence in the reported signals. Of 25 variants that meet this requirement, 23 are externally replicated, and the remaining 2 may be true associations that are yet to be replicated owing to a lack of coverage or power in the replication datasets.

The design of our study incorporates genetic signals for every stage in the disease progression into a single phenotype. This includes establishment of infection, viral replication, inflammatory lung injury and hypoxaemic respiratory failure. Although we can have considerable confidence that the replicated associations with critical COVID-19 we report are robust, we cannot determine at which stage in the disease process, or in which tissue, the relevant biological mechanisms are active.

## Conclusions

These genetic associations identify biological mechanisms that may underlie the development of life-threatening COVID-19, several of which may be amenable to therapeutic targeting. Furthermore, we demonstrate the value of WGS for fine-mapping loci in a complex trait. In the context of the ongoing global pandemic, translation to clinical practice is an urgent priority. As with our previous work, biological and molecular studies—and, where appropriate, large-scale randomized trials—will be essential before our findings can be translated into clinical practice.

## Methods

### Ethics

GenOMICC study: GenOMICC was approved by the following research ethics committees: Scotland ‘A’ Research Ethics Committee (15/SS/0110) and Coventry and Warwickshire Research Ethics Committee (England, Wales and Northern Ireland) (19/WM/0247). Current and previous versions of the study protocol are available at https://genomicc.org/protocol/. 100,000 Genomes project: the 100,000 Genomes project was approved by the East of England—Cambridge Central Research Ethics Committee (REF 20/EE/0035). Only individuals from the 100,000 Genomes project for whom WGS data were available and who consented for their data to be used for research purposes were included in the analyses. UK Biobank study: ethical approval for the UK Biobank was previously obtained from the North West Centre for Research Ethics Committee (11/NW/0382). The work described herein was approved by UK Biobank under application number 26041. Geisinger Health Systems (GHS) study: approval for DiscovEHR analyses was provided by the GHS Institutional Review Board under project number 2006-0258. AncestryDNA study: all data for this research project were from individuals who provided prior informed consent to participate in AncestryDNA’s Human Diversity Project, as reviewed and approved by our external institutional review board, Advarra (formerly Quorum). All data were de-identified before use. Penn Medicine Biobank study: appropriate consent was obtained from each participant regarding the storage of biological specimens, genetic sequencing and genotyping, and access to all available EHR data. This study was approved by the institutional review board of the University of Pennsylvania and complied with the principles set out in the Declaration of Helsinki. Informed consent was obtained for all study participants. 23andMe study: participants in this study were recruited from the customer base of 23andMe, a personal genetics company. All individuals included in the analyses provided informed consent and answered surveys online according to the 23andMe protocol for research in humans, which was reviewed and approved by Ethical and Independent Review Services, a private institutional review board (http://www.eandireview.com).

### Recruitment of cases (patients with COVID-19)

Patients were recruited to the GenOMICC study in 224 UK intensive care units (https://genomicc.org). All individuals had confirmed COVID-19 according to local clinical testing and were deemed, in the view of the treating clinician, to require continuous cardiorespiratory monitoring. In UK practice this kind of monitoring is undertaken in high-dependency or intensive care units.

### Recruitment of control individuals

#### Mild or asymptomatic control individuals

Participants were recruited to the mild COVID-19 cohort on the basis of having experienced mild (non-hospitalized) or asymptomatic COVID-19. Participants volunteered to take part in the study via a microsite and were required to self-report the details of a positive COVID-19 test. Volunteers were prioritized for genome sequencing on the basis of demographic matching with the critical COVID-19 cohort considering self-reported ancestry, sex, age and location within the UK. We refer to this cohort as the COVID-19 mild cohort.

#### Control individuals from the 100,000 Genomes project

Participants were enrolled in the 100,000 Genomes Project from families with a broad range of rare diseases, cancers and infection by 13 regional NHS Genomic Medicine Centres across England and in Northern Ireland, Scotland and Wales. For this analysis, participants for whom a positive SARS-CoV-2 test had been recorded as of March 2021 were not included owing to uncertainty in the severity of COVID-19 symptoms. Only participants for whom genome sequencing was performed from blood-derived DNA were included and participants with haematological malignancies were excluded to avoid potential tumour contamination.

### DNA extraction

For severe cases of COVID-19 and mild cohort controls, DNA was extracted from whole blood either manually using a Nucleon Kit (Cytiva) and resuspended in 1 ml TE buffer pH 7.5 (10 mM Tris-Cl pH 7.5, 1 mM EDTA pH 8.0), or automated on the Chemagic 360 platform using the Chemagic DNA blood kit (PerkinElmer) and re-suspended in 400 μl elution buffer. The yield of the DNA was measured using Qubit and normalized to 50 ng μl^−1^ before sequencing. For the 100,000 Genomes Project samples, DNA was extracted from whole blood at designated extraction centres following sample handling guidance provided by Genomics England and NHS England.

### WGS

Sequencing libraries were generated using the Illumina TruSeq DNA PCR-Free High Throughput Sample Preparation kit and sequenced with 150-bp paired-end reads in a single lane of an Illumina Hiseq X instrument (for 100,000 Genomes Project samples) or a NovaSeq instrument (for the COVID-19 critical and mild cohorts).

#### Sequencing data quality control

All genome sequencing data were required to meet minimum quality metrics and quality control measures were applied for all genomes as part of the bioinformatics pipeline. The minimum data requirements for all genomes were: more than 85 × 10^−9^ bases with *Q* ≥ 30 and at least 95% of the autosomal genome covered at 15× or higher calculated from reads with mapping quality greater than 10 after removing duplicate reads and overlapping bases, after adaptor and quality trimming. Assessment of germline cross-sample contamination was performed using VerifyBamID and samples with more than 3% contamination were excluded. Sex checks were performed to confirm that the sex reported for a participant was concordant with the sex inferred from the genomic data.

### WGS alignment and variant calling

#### COVID-19 cohorts

For the critical and mild COVID-19 cohorts, sequencing data alignment and variant calling were performed with Genomics England pipeline 2.0, which uses the DRAGEN software (v.3.2.22). Alignment was performed to genome reference GRCh38 including decoy contigs and alternative haplotypes (ALT contigs), with ALT-aware mapping and variant calling to improve specificity.

#### 100,000 Genomes Project cohort

All genomes from the 100,000 Genomes Project cohort were analysed with the Illumina North Star Version 4 Whole Genome Sequencing Workflow (NSV4, v.2.6.53.23); which comprises the iSAAC Aligner (v.03.16.02.19) and Starling Small Variant Caller (v.2.4.7). Samples were aligned to the Homo Sapiens NCBI GRCh38 assembly with decoys.

A subset of the genomes from the cancer program of the 100,000 Genomes Project were reprocessed (alignment and variant calling) using the same pipeline used for the COVID-19 cohorts (DRAGEN v.3.2.22) for equity of alignment and variant calling.

### Aggregation

Aggregation was conducted separately for the samples analysed with Genomics England pipeline 2.0 (severe cohort, mild cohort, cancer-realigned 100,000 Genomes Project) and those analysed with the Illumina North Star Version 4 pipeline (100,000 Genomes Project).

For the first three, the WGS data were aggregated from single-sample gVCF files to multi-sample VCF files using GVCFGenotyper (GG) v.3.8.1, which accepts gVCF files generated by the DRAGEN pipeline as input. GG outputs multi-allelic variants (several ALT variants per position on the same row), and for downstream analyses the output was decomposed to bi-allelic variants per row using the software vt v.0.57721. We refer to the aggregate as aggCOVID_vX, in which X is the specific freeze.The analysis in this manuscript uses data from freeze v.4.2 and the respective aggregate is referred to as aggCOVID_v4.2.

Aggregation for the 100,000 Genomes Project cohort was performed using Illumina’s gvcfgenotyper v.2019.02.26, merged with bcftools v.1.10.2 and normalized with vt v.0.57721.

### Sample quality control

Samples that failed any of the following four BAM-level quality control filters: freemix contamination > 3%, mean autosomal coverage < 25×, per cent mapped reads < 90% or per cent chimeric reads > 5% were excluded from the analysis.

In addition, a set of VCF-level quality control filters were applied after aggregation on all autosomal bi-allelic single-nucleotide variants (SNVs) (akin to gnomAD v.3.1)^18^. Samples were filtered out on the basis of the residuals of eleven quality control metrics (calculated using bcftools) after regressing out the effects of sequencing platform and the first three ancestry assignment principal components (PCs) (including all linear, quadratic and interaction terms) taken from the sample projections onto the SNP loadings from the individuals of 1000 Genomes Project phase 3 (1KGP3). Samples were removed that were four median absolute deviations (MADs) above or below the median for the following metrics: ratio of heterozygous to homozygous, ratio of insertions to deletions, ratio of transitions to transversions, total deletions, total insertions, total heterozygous SNPs, total homozygous SNPs, total transitions and total transversions. For the number of total singletons (SNPs), samples were removed that were more than 8 MADs above the median. For the ratio of heterozygous to homozygous alternative SNPs, samples were removed that were more than 4 MADs above the median.

After quality control, 79,803 individuals were included in the analysis with the breakdown according to cohort shown in Supplementary Table 2.

### Selection of high-quality independent SNPs

We selected high-quality independent variants for inferring kinship coefficients, performing PCA, assigning ancestry and for the conditioning on the genetic relatedness matrix by the logistic mixed model of SAIGE and SAIGE-GENE. To avoid capturing platform and/or analysis pipeline effects for these analyses, we performed very stringent variant quality control as described below.

#### High-quality common SNPs

We started with autosomal, bi-allelic SNPs which had a frequency of higher than 5% in aggV2 (100,000 Genomes Project participant aggregate) and in the 1KGP3. We then restricted to variants that had missingness < 1%, median genotype quality control > 30, median depth (DP) ≥ 30 and at least 90% of heterozygote genotypes passing an ABratio binomial test with *P* value > 10^−2^ for aggV2 participants. We also excluded variants in complex regions from the list available in https://genome.sph.umich.edu/wiki/Regions_of_high_linkage_disequilibrium_(LD) (lifted over for GRCh38), and variants where the REF/ALT combination was CG or AT (C/G, G/C, A/T, T/A). We also removed all SNPs that were out of Hardy–Weinberg equilibrium (HWE) in any of the AFR, EAS, EUR or SAS super-populations of aggV2, with a *P* value cut-off of *P*_HWE_ < 10^−5^. We then LD-pruned using PLINK v.1.9 with *r*^2^ = 0.1 and in 500-kb windows. This resulted in a total of 63,523 high-quality sites from aggV2.

We then extracted these high-quality sites from the aggCOVID_v4.2 aggregate and further applied variant quality filters (missingness < 1%, median quality control > 30, median depth ≥ 30 and at least 90% of heterozygote genotypes passing an ABratio binomial test with *P* value > 10^−2^), per batch of sequencing platform (that is, HiseqX, NovaSeq6000).

After applying variant filters in aggV2 and aggCOVID_v4.2, we merged the genomic data from the two aggregates for the intersection of the variants, which resulted in a final total of 58,925 sites.

#### High-quality rare SNPs

We selected high-quality rare (MAF < 0.005) bi-allelic SNPs to be used with SAIGE for aggregate variant testing (AVT) analysis. To create this set, we applied the same variant quality control procedure as with the common variants: We selected variants that had missingness < 1%, median quality control > 30, median depth ≥ 30 and at least 90% of heterozygote genotypes passing an ABratio binomial test with *P* value > 10^−2^ per batch of sequencing and genotyping platform (that is, HiSeq + NSV4, HiSeq + Pipeline 2.0, NovaSeq + Pipeline 2.0). We then subsetted those to the following groups of minor allele count (MAC) and MAF categories: MAC 1, 2, 3, 4, 5, 6–10, 11–20, MAC 20–MAF 0.001, MAF 0.001–0.005.

### Relatedness, ancestry and principal components

#### Kinship

We calculated kinship coefficients among all pairs of samples using the software PLINK v.2.0 and its implementation of the KING robust algorithm. We used a kinship cut-off of <0.0442 to select unrelated individuals with argument “–king-cutoff”.

#### Genetic ancestry prediction

To infer the ancestry of each individual, we performed principal component analysis (PCA) on unrelated 1KGP3 individuals with GCTA v.1.93.1_beta software using high-quality common SNPs^43^, and inferred the first 20 PCs. We calculated loadings for each SNP, which we used to project aggV2 and aggCOVID_v4.2 individuals onto the 1KGP3 PCs. We then trained a random forest algorithm from the R package randomForest with the first 10 1KGP3 PCs as features and the super-population ancestry of each individual as labels. These were ‘AFR’ for individuals of African ancestry, ‘AMR’ for individuals of American ancestry, ‘EAS’ for individuals of East Asian ancestry, ‘EUR’ for individuals of European ancestry and ‘SAS’ for individuals of South Asian ancestry. We used 500 trees for the training. We then used the trained model to assign a probability of belonging to a certain super-population class for each individual in our cohorts. We assigned individuals to a super-population when class probability ≥ 0.8. Individuals for whom no class had probability ≥ 0.8 were labelled as ‘unassigned’ and were not included in the analyses.

#### PCA

After labelling each individual with predicted genetic ancestry, we calculated ancestry-specific PCs using GCTA v.1.93.1_beta^43^. We computed 20 PCs for each of the ancestries that were used in the association analyses (AFR, EAS, EUR and SAS).

### Variant quality control

Variant quality control was performed to ensure high quality of variants and to minimize batch effects due to using samples from different sequencing platforms (NovaSeq6000 and HiseqX) and different variant callers (Strelka2 and DRAGEN). We first masked low-quality genotypes setting them to missing, merged aggregate files and then performed additional variant quality control separately for the two major types of association analyses, GWAS and AVT, which concerned common and rare variants, respectively.

#### Masking

Before any analysis, we masked low-quality genotypes using the bcftools setGT module. Genotypes with DP < 10, genotype quality (GQ) < 20 and heterozygote genotypes failing an ABratio binomial test with *P* value < 10^−3^ were set to missing.

We then converted the masked VCF files to PLINK and bgen format using PLINK v.2.0.

#### Merging of aggregate samples

Merging of aggV2 and aggCOVID_v4.2 samples was done using PLINK files with masked genotypes and the merge function of PLINK v.1.9^44^. for variants that were found in both aggregates.

### GWAS analyses

#### Variant quality control

We restricted all GWAS analyses to common variants applying the following filters using PLINK v.1.9: MAF > 0 in both cases and controls, MAF > 0.5% and MAC > 20, missingness < 2%, differential missingness between cases and controls, mid-*P* value < 10^−5^, HWE deviations on unrelated controls, mid-*P* value < 10^−6^. Multi-allelic variants were in addition required to have MAF > 0.1% in both aggV2 and aggCOVID_v4.2.

#### Control–control quality control filter

100,000 Genomes Project aggV2 samples that were aligned and genotype called with the Illumina North Star version 4 pipeline represented the majority of control samples in our GWAS analyses, whereas all of the cases were aligned and called with Genomics England pipeline 2.0 (Supplementary Table 1). Therefore, the alignment and genotyping pipelines partially match the case–control status, which necessitates additional filtering for adjusting for between-pipeline differences in alignment and variant calling. To control for potential batch effects, we used the overlap of 3,954 samples from the Genomics England 100,000 Genomes Project participants that were aligned and called with both pipelines. For each variant, we computed and compared between platforms the inferred allele frequency for the population samples. We then filtered out all variants that had >1% relative difference in allele frequency between platforms. The relative difference was computed on a per-population basis for EUR (*n* = 3,157), SAS (*n* = 373), AFR (*n* = 354) and EAS (*n* = 81).

#### Model

We used a two-step logistic mixed model regression approach as implemented in SAIGE v.0.44.5 for single-variant association analyses. In step 1, SAIGE fits the null mixed model and covariates. In step 2, single-variant association tests are performed with the saddlepoint approximation (SPA) correction to calibrate unbalanced case–control ratios. We used the high-quality common variant sites for fitting the null model and sex, age, age^2^, age-by-sex and 20 PCs as covariates in step 1. The PCs were computed separately by predicted genetic ancestry (that is, EUR-specific, AFR-specific and so on), to capture subtle structure effects.

#### Analyses

All analyses were done on unrelated individuals with a pairwise kinship coefficient < 0.0442. We conducted GWAS analyses per predicted genetic ancestry, for all populations for which we had more than 100 cases and more than 100 controls (AFR, EAS, EUR and SAS).

#### Multiple testing correction

As our study is testing variants that were directly sequenced by WGS and not imputed, we calculated the *P* value significance threshold by estimating the effective number of tests. After selecting the final filtered set of tested variants for each population, we LD-pruned in a window of 250 kb and *r*^2^ = 0.8 with PLINK 1.9. We then computed the Bonferroni-corrected *P* value threshold as 0.05 divided by the number of LD-pruned variants tested in the GWAS. The *P* value thresholds that were used for declaring statistical significance are provided in Supplementary Table 5.

#### LD-clumping

We used PLINK v.1.9 to do clumping of variants that were genome-wide significant for each analysis with *P*1 set to per-population *P* value from Supplementary Table 5, *P*2 = 0.01, clump distance 1,500 kb and *r*^2^ = 0.1.

#### Conditional analysis and signal independence

To find the set of independent variants in the per-population analyses, we performed a step-wise conditional analysis with the GWAS summary statistics for each population using GCTA 1.9.3 –cojo-slct function^43^. The parameters for the function were pval = 2.2 × 10^−8^, a distance of 10,000 kb and a colinear threshold of 0.9 (ref. ^45^). For establishing independence of multi-ancestry meta-analysis signals from per-population discovered signals, we performed LD-clumping using the meta-analysis summaries and identified signals with no overlap with the LD-clumped results from the per-population analyses. In addition to the GCTA-cojo analysis, we also performed confirmatory individual-level conditional analysis as implemented in SAIGE. For every lead variant signal (including the multi-ancestry meta-analysis signals), we conditioned on the lead variants of all other signals identified as independent by GCTA-cojo and located on the same chromosome with option –condition of SAIGE (Supplementary Table 6).

#### Fine-mapping

We performed fine-mapping for genome-wide-significant signals using theR package SusieR v.0.11.42^13^. For each genome-wide-significant variant locus, we selected the variants 1.5 Mbp on each side and computed the correlation matrix among them with PLINK v.1.9. We then ran the susieR summary-statistics-based function susie_rss and provided the summary *z* scores from SAIGE (that is, effect size divided by its standard error) and the correlation matrix computed with the same samples that were used for the corresponding GWAS. We required coverage ≥``{=html}0.95 for each identified credible set and minimum and median absolute correlation coefficients (purity) of *r* = 0.1 and 0.5, respectively.

#### Functional annotation of credible sets

We annotated all variants included in each credible set identified by SusieR using the online Variant Effect Predictor (VEP) v.104 and selected the worst consequence across GENCODE basic transcripts (Supplementary Information). We also ranked each variant within each credible set according to the predicted consequence and the ranking was based on the table provided by Ensembl: https://www.ensembl.org/info/genome/variation/prediction/predicted_data.html.

#### Multi-ancestry meta-analysis

We performed a meta-analysis across all ancestries using an inverse-variance weighting method and control for population stratification for each separate analysis in the METAL software^46^. The meta-analysed variants were filtered for variants with heterogeneity *P* value *P* < 2.22 × 10^−8^ and variants that are not present in at least half of the individuals. We used the meta R package to plot forest plots of the clumped multi-ancestry meta-analysis variants^47^.

#### LD-based validation of lead GWAS signals

To quantify the support for genome-wide-significant signals from nearby variants in LD, we assessed the internal consistency of GWAS results of the lead variants and their surroundings. To this end, we compared observed *z*-scores at lead variants with the expected *z*-scores based on those observed at neighbouring variants. Specifically, we computed the observed *z*-score for a variant *i* as ${s}_{i}=\hat{\beta }/{\hat{\sigma }}_{\hat{\beta }}$ and, following a previous approach^48^, the imputed *z*-score at a target variant *t* as

$${\hat{s}}_{t}={{\boldsymbol{\Sigma }}}_{t,P}{\left({{\boldsymbol{\Sigma }}}_{P,P}+\lambda {\bf{I}}\right)}^{-1}{{\bf{s}}}_{P}$$

where ${{\bf{s}}}_{P}$ are the observed z-scores at a set *P* of predictor variants, ${{\boldsymbol{\Sigma }}}_{x,y}$ is the empirical correlation matrix of dosage coded genotypes computed on the GWAS sample between the variants in *x* and *y*, and *λ* is a regularization parameter set to 10^−5^. The set *P* of predictor variants consisted of all variants within 100 kb of the target variant with a genotype correlation with the target variant greater than 0.25. This approach is similar to one proposed recently^49^.

#### Stratified analysis

We performed sex-specific analysis (male and female individuals separately) as well as analysis stratified by age (that is, participants of younger than 60 years old and 60 years old or above) for the EUR ancestry group. To compare the effect of variants within groups for the age- and sex-stratified analysis we first adjusted the effect and error of each variant for the standard deviation of the trait in each stratified group and then used the following *t*-statistic, as in previous studies^50,51^

$$t=\frac{{b}_{1}-{b}_{2}}{\sqrt{{\rm{s}}{{\rm{e}}}_{1}^{2}+{\rm{s}}{{\rm{e}}}_{2}^{2}-2\times r{\rm{s}}{{\rm{e}}}_{1}\times r{\rm{s}}{{\rm{e}}}_{2}}}$$

where *b*_1_ is the adjusted effect for group 1, *b*_2_ is the adjusted effect for group 2, se_1_ and se_2_ are the adjusted standard errors for groups 1 and 2, respectively, and *r* is the Spearman rank correlation between groups across all genetic variants.

#### Replication

To generate a replication set, we conducted a meta-analysis of data from 23andMe, together with a meta-analysis of the COVID-19 HGI data freeze 6 (hospitalized COVID versus population) GWAS (B2 analysis), including all genetic ancestries. Although the HGI programme included an analysis designed to mirror the GenOMICC study (analysis ‘A2’), most of these cases come from GenOMICC and are already included in the discovery cohort. We therefore used the broader hospitalized phenotype (‘B2’) for replication.

To account for signal due to sample overlap we performed a mathematical subtraction from HGI v.6 B2, of the GenOMICC GWAS of European genetic ancestry. Publicly available HGI data were downloaded from https://www.covid19hg.org/results/r6/. The subtraction was performed using the MetaSubtract package (v.1.60) for R (v.4.0.2) after removing variants with the same genomic position and using the lambda.cohorts with genomic inflation calculated on the GenOMICC summary statistics.

We calculated a multi-ancestry meta-analysis for the three ancestries with summary statistics in 23andMe—African, Latino and European—using variants that passed the 23andMe ancestry quality control, with imputation score > 0.6 and with MAF > 0.005, before performing a final meta-analysis of 23andMe and HGI B2 without GenOMICC to create the final replication set. Meta-analysis was performed using METAL^46^, with the inverse-variance weighting method (STDERR mode) and genomic control ON. We considered that a hit was replicated if the direction of effect in the GenOMICC-subtracted HGI summary statistics was the same as in our GWAS, and the *P* value was significant after Bonferroni correction for the number of attempted replications (pval < 0.05/25). If the main hit was not present in the HGI–23andMe meta-analysis or if the hit was not replicating, we looked for replication in variants in high LD with the top variant (*r*^2^ > 0.9), which helped replicate two regions.

To attempt additional replication of two associations, we performed a multi-ancestry meta-analysis across five continental ancestry groups in the UK Biobank, AncestryDNA, Penn Medicine Biobank and GHS, totalling 9,937 hospitalized cases of COVID-19 and 1,059,390 controls (COVID-19 negative or unknown). Hospitalization status (positive, negative or unknown) was determined on the basis of COVID-19-related ICD10 codes U071, U072, U073 in variable ‘diag_icd10’ (table ‘hesin_diag’) in the UK Biobank study; self-reported hospitalization due to COVID-19 in the AncestryDNA study; and medical records in the GHS and Penn Medicine Biobank studies. Association analyses in each study were performed using the genome-wide Firth logistic regression test implemented in REGENIE. In this implementation, Firth’s approach is applied when the *P* value from a standard logistic regression score test is less than 0.05. We included in step 1 of REGENIE (that is, prediction of individual trait values based on the genetic data) directly genotyped variants with MAF > 1%, missingness < 10%, HWE test *P* > 1 × 10^−15^ and LD-pruning (1,000 variant windows, 100 variant sliding windows and *r*^2^ < 0.9). The association model used in step 2 of REGENIE included as covariates age, age^2^, sex, age-by-sex, and the first 10 ancestry-informative PCs derived from the analysis of a stricter set of LD-pruned (50 variant windows, 5 variant sliding windows and *r*^2^ < 0.5) common variants from the array (imputed for the GHS study) data. Within each study, association analyses were performed separately for five different continental ancestries defined on the basis of the array data: African (AFR), Hispanic or Latin American (HLA), East Asian (EAS), European (EUR) and South Asian (SAS). Results were subsequently meta-analysed across studies and ancestries using an inverse-variance-weighted fixed-effects meta-analysis.

### HLA imputation and association analysis

HLA types were imputed at two-field (four-digit) resolution for all samples within aggV2 and aggCOVID_v4.2 for the following seven loci: HLA-A, HLA-C, HLA-B, HLA-DRB1, HLA-DQA1, HLA-DQB1 and HLA-DPB1, using the HIBAG package in R^15^. At the time of writing, HLA types were also imputed for $\mathop{8}\limits^{ \sim }$ 2% of samples using HLA*LA^52^. Inferred HLA alleles between HIBAG and HLA*LA were more than 96% identical at four-digit resolution. HLA association analysis was run under an additive model using SAIGE, in an identical manner to the SNV GWAS. The multi-sample VCF of aggregated HLA type calls from HIBAG was used as input in cases in which any allele call with posterior probability (*T*) < 0.5 were set to missing.

### AVT

AVT on aggCOVID_v4.2 was performed using SKAT-O as implemented in SAIGE-GENE v.0.44.5^17^ on all protein-coding genes. Variant and sample quality control for the preparation and masking of the aggregate files have been described elsewhere. We further excluded SNPs with differential missingness between cases and controls (mid-*P* value < 10^−5^) or a site-wide missingness above 5%. Only bi-allelic SNPs with MAF < 0.5% were included.

We filtered the variants to include in the AVT by applying two functional annotation filters: a putative loss of function (pLoF) filter, in which only variants that are annotated by LOFTEE^18^ as high-confidence loss of function were included; and a more lenient (missense) filter, in which variants that have a consequence of missense or worse as annotated by VEP, with a CADD_PHRED score of ≥10, were also included. All variants were annotated using VEP v99. SAIGE-GENE was run with the same covariates used in the single variant analysis: sex, age, age^2^, age-by-sex and 20 (population-specific) PCs generated from common variants (MAF ≥ 5%).

We ran the tests separately by genetically predicted ancestry, as well as across all four ancestries as a mega-analysis. We considered a gene-wide-significant threshold on the basis of the genes tested per ancestry, correcting for the two masks (pLoF and missense; Supplementary Table 14).

### Post-GWAS analysis

#### TWASs

We performed TWASs in the MetaXcan framework and the GTEx v.8 eQTL and splicing quantitative trait loci (sQTL) MASHR-M models available for download in http://predictdb.org/. We first calculated, using the European summary statistics, individual TWASs for whole blood and lung with the S-PrediXcan function^53,54^. Then we performed a metaTWAS including data from all tissues to increase statistical power using s-MultiXcan^55^. We applied the Bonferroni correction to the results to choose significant genes and introns for each analysis.

#### Colocalization analysis

Significant genes from the TWAS, splicing TWAS, metaTWAS and splicing metaTWAS, as well as genes for which one of the top variants was a significant eQTL or sQTL, were selected for a colocalization analysis using the coloc R package^56^. We chose the lead SNPs from the European ancestry GWAS summary statistics and a region of ±200 kb around each SNP to do the colocalization with the identified genes in the region. GTEx v.8 whole-blood and lung tissue summary statistics and eqtlGen (which has blood eQTL summary statistics for more than 30,000 individuals) were used for the analysis^22,57^. We first performed a sensitivity analysis of the posterior probability of colocalization (PP_H4_) on the prior probability of colocalization (*P*_12_), going from *P*_12_ = 10^−8^ to *P*_12_ = 10^−4^, with the default threshold being *P*_12_ = 10^−5^. eQTL signal and GWAS signals were deemed to colocalize if these two criteria were met: (1) at *P*_12_ = 5 × 10^−5^ the probability of colocalization PP_H4_ > 0.5; and (2) at *P*_12_ = 10^−5^ the probability of independent signal (PP_H3_) was not the main hypothesis (PP_H3_ < 0.5). These criteria were chosen to allow eQTLs with weaker *P* values, owing to lack of power in GTEx v.8, to be colocalized with the signal when the main hypothesis using small priors was that there was not any signal in the eQTL data.

As the chromosome 3-associated interval is larger than 200 kb, we performed additional colocalization including a region up to 500 kb, but no further colocalizations were found.

#### Mendelian randomization

We performed GSMR^23^ in a replicated outcome study design. As exposures, we used the pQTLs from the INTERVAL study^24^. We used the 1000 Genomes Project imputed data of the Health and Retirement Study (HRS) (*n* = 8,557) as the LD reference data required for GSMR analysis. The HRS data are available from dbGap (accession number: phs000428).

GSMR was undertaken using all exposures for which we were able to identify two or more independent SNPs associated with the exposure (*P* value(exposure) < 5 × 10^−8^; LD clumping ±1 Mb, *r*^2^ < 0.05; HEIDI-outlier filtering test, for the removal of SNPs with evidence of horizontal pleiotropy, was performed at the default threshold value of 0.01). Using GSMR, we identified those proteins implicated in determining COVID-19 severity in the new GenOMICC results (following genomic-control correction for inflation) at a false discovery rate (FDR) of less than 0.05, and attempted replication in the GWAS of ‘Hospitalized COVID versus population’ (phenotype B2) of the COVID-19 HGI (ref. ^58^) having excluded the previous GenOMICC results. We achieved this by mathematically removing the contribution of GenOMICC^1^ from the meta-analysis. We considered as replicated those results that passed a Bonferroni-corrected *P* value threshold, correcting for the total number of replication tests attempted (that is, the number of observations from the discovery set with FDR < 0.05).

#### Heritability

For the SNP-based narrow-sense heritabilities of severe COVID-19 and HGI COVID phenotypes, both high-definition likelihood (HDL) and LD score regression (LDSC)^59^ methods were applied. The HGI summary statistics were based on the GWAS analysis of all available samples, in which the majority were European populations (see https://www.covid19hg.org/results/r6/). The munge_sumstats.py procedure in the LDSC software was used to harmonize the summary statistics, and in LDSC, the reference panel was built using the 1000 Genome European samples with SNPs that have MAF > 0.05. As both HDL and LDSC are based on GWAS summary *z*-score statistics, the estimated heritabilities are thus on the observed scale.

#### Enrichment analysis

Enrichment analysis was performed to identify ontologies in which discovery genes were overrepresented. Using the XGR algorithm (http://galahad.well.ox.ac.uk/XGR)^60^, 19 genes identified through lead variant proximity, credible variant sets, mutation consequence and TWAS analyses were tested for enrichment in disease ontology^61^, gene ontologies (biological process, molecular function and cellular component)^62^ and KEGG^63^ and Reactome^64^ pathways using default settings. This generated a *P* value and FDR for overrepresentation of genes within each of the ontologies (Supplementary Table 15).

### Reporting summary

Further information on research design is available in the Nature Research Reporting Summary linked to this paper.

## Online content

Any methods, additional references, Nature Research reporting summaries, source data, extended data, supplementary information, acknowledgements, peer review information; details of author contributions and competing interests; and statements of data and code availability are available at 10.1038/s41586-022-04576-6.

### Supplementary information

Supplementary Information This file contains Supplementary Figures, Supplementary Tables and Supplementary References

Reporting Summary

Peer Review File

## Figures and Tables

**Extended Data Fig. 3:**
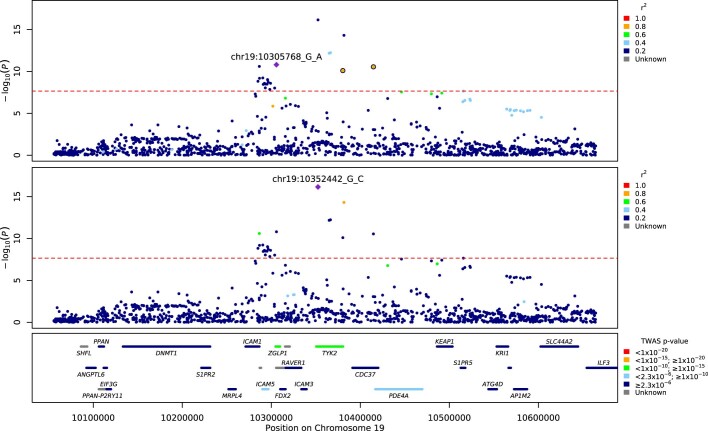
Regional detail showing fine-mapping to identify two adjacent independent signals on chromosome 19. Top two panels: variants in LD with the lead variants shown. The variants that are included in two independent credible sets are displayed with black outline circles. ${r}^{2}$ values in the legend denote upper limits, 0.2=[0,0.2], 0.4=[0.2,0.4], 0.6=[0.4,0.6], 0.8=[0.6,0.8],1=[0.8,1]. Bottom panel: locations of protein-coding genes, coloured by TWAS $P$-value. The red dashed line shows the Bonferroni-corrected $P$-value=$2.2\times {10}^{-8}$ for Europeans.

**Extended Data Table 1:**
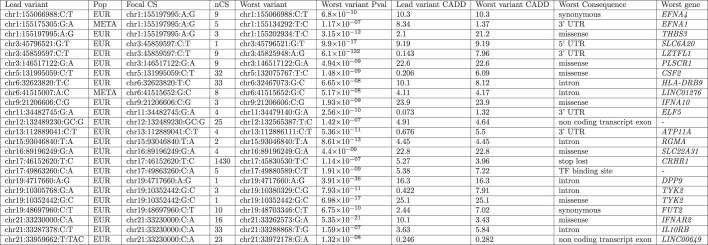
Fine-mapping results for lead variants and worst consequence variant in each credible set

Fine-mapping was performed in EUR for all variants except chr6:41515007:A:C, which was fine-mapped in the SAS population for which the signal was strongest among the per-population analyses. The lead variant chr2:60480453:A:G (rs1123573) that was discovered in multi-ancestry meta-analysis is not included in the table as fine-mapping did not generate any credible sets with the required posterior inclusion probability of >0.95 for any of the populations. Focal CS is the index SNP that was used for fine-mapping with SusieR, 1.5 Mb on each side. nCS indicates the number of variants included in each credible set. Consequence annotation for all variants across credible sets was generated using VEP v.104 and the worst consequence across GENCODE basic transcripts was chosen. All variants were ranked according to their consequence type and chr:pos_hg38_:ref_hg38_:alt, *P* value and CADD score are provided for the variant with the worst consequence across all variants in each credible set.

**Extended Data Table 2:**
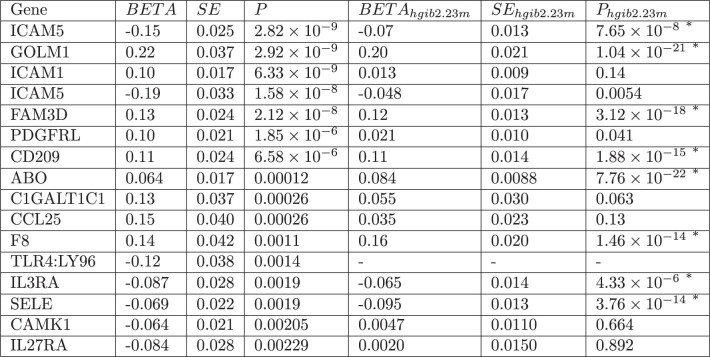
Identification of 16 proteins by the GSMR analysis for COVID-19 severity at FDR < 0.05

We report the effect size BETA, the standard error SE and the P value *P* for the GenOMICC analysis and the replication with HGI B2 and 23andme meta-analysis. An asterisk (*) next to the replication *P* value (*P*_hgib2.23m_) indicates that the protein result is replicated with concordant direction of effect. We considered as replicated those results that passed a Bonferroni correction of the *P* values of the replicated outcome Mendelian randomization.

## Data Availability

All data are available through https://genomicc.org/data. This includes downloadable summary data tables and instructions for applying to access individual-level data. Individual-level genome sequence data for the COVID-19 severe and mild cohorts can be analysed by qualified researchers in the UK Outbreak Data Analysis Platform at the University of Edinburgh by application at https://genomicc.org/data. Genomic data for the 100,000 Genomes Project participants and a subset of COVID-19 cases are also available through the Genomics England research environment, which can be accessed by application at https://www.genomicsengland.co.uk/join-a-gecip-domain. The full GWAS summary statistics for the 23andMe discovery dataset are available through 23andMe to qualified researchers under an agreement with 23andMe that protects the privacy of the 23andMe participants. More information and access to the data are provided at https://research.23andMe.com/dataset-access/.

## Code availability

Code to calculate the imputation of *P* values based on LD SNPs is available at https://github.com/baillielab/GenOMICC_GWAS.
