# Supplementary material for: Whole-genome sequencing reveals host factors underlying critical COVID-19
Source: Nature. 2022 Mar 7;607(7917):97–103. doi: 10.1038/s41586-022-04576-6 (PMC9259496; doi:10.1038/s41586-022-04576-6)
Supplement: Supplementary file 1 — This file contains Supplementary Figures, Supplementary Tables and Supplementary References [file 41586_2022_4576_MOESM1_ESM.pdf]

nature portfolio

https://doi.org/10.1038/s41586-022-04576-6

Supplementary information

Whole-genome sequencing reveals host factors underlying critical COVID-19

In the format provided by the authors and unedited

Whole genome sequencing identifies multiple loci for critical illness caused by Covid-19
Supplementary material

Contents

Cohort description **5**
Pre-QC description . . . 5
Post-QC description . . . 5
SampleQC . . . 6

PCA and ancestry **8**

Cohort characteristics **13**
Disease characteristics . . . 13
Demographics . . . 14

GWAS **16**
Per-population GWAS results . . . 16
LD-based validation of lead GWAS signals . . . 18
Individual-level conditional analysis . . . 19
Comparison to 2020 GenOMICC microarray study . . . 20
Association signal forest plots by genetic ancestry . . . 21
Replication . . . 23
Genetic fine-mapping . . . 28
Fine-mapping analysis for lead variants . . . 28
Fine-mapping check for rare variants . . . 30
Sex- and age- stratified analysis . . . 36
Matched case-control analysis . . . 37
GWAS analysis using the mild Covid-19 cohort only as controls . . . 41
Heritability . . . 41

TWAS **43**
eQTL TWAS . . . 43
Colocalisation sensitivity analysis . . . 45
Colocalisation Summary . . . 52
Mendelian Randomisation . . . 53

Aggregate variant testing (AVT) **55**
Masks and Model . . . 55
AVT results . . . 55

HLA Inference and Association Tests **58**
HLA imputation using HIBAG . . . 58
HLA inference using HLA*LA and concordance between HIBAG and HLA*LA callsets . . 58
HLA Association Tests . . . 59
HLA conditional Analysis . . . 59

Enrichment analysis **62**

Meta-analysis by information content (MAIC) **63**

References **64**

List of Figures

1 Histograms of demographic features of each group 6
2 Distribution of quality metrics 7
3 PCA 1KGP3 projection plots 8
4 EUR PCs 1-10 for severe (cases) and mild + 100K individuals (controls). 9
5 SAS PCs 1-10 for severe (cases) and mild + 100K individuals (controls). 10
6 AFR PCs 1-10 for severe (cases) and mild + 100K individuals (controls). 11
7 EAS PCs 1-10 for severe (cases) and mild + 100K individuals (controls). 12
8 Comorbidity among 100k participants 13
9 Demographic characteristics of Covid-19 severe, Covid-19 mild and 100K cohorts. Panel A displays the numeric breakdown into males and females across the different cohorts. Panel B displays the age distribution across cohorts. 14
10 BMI distribution 15
11 Manhattan plots showing GWAS results for each population cohort. 17
12 *P*-values with leave-one procedure for lead variants of the EUR analysis 18
13 Forest plots by genetic ancestry for all lead signals. 22
14 Forest plots comparing different studies 27
15 Locuszoom figures or per-population analyses 33
16 Locuszoom figures for the multi-ancestry meta-analysis 35
17 Manhattan plot for *t*-test *P*-values obtained from comparison of stratified GWAS analyses by age and sex 36
18 Sex, age and BMI distributions for unmatched and matched controls 38
19 Genome-wide Results for effect size (BETA) and *P*-values for a EUR SAIGE GWAS analysis 39
20 Comparison of analyses with matched and unmatched controls for EUR 39
21 Comparison of effect sizes between analyses with matched and unmatched controls for signals discovered in multi-ancestry meta-analysis 40
22 Heritability estimates 42
23 TWAS results from analysis of eQTL models from whole blood and lung tissues in GTEXv8. 43
24 TWAS results in GTEXv8 44
25 Sensitivity analysis for colocalisation of TWAS-significant loci in blood (eQTLgen) . 46
26 Sensitivity analysis for colocalisation of TWAS-significant loci in blood (GTEXv8) . 48
27 Sensitivity analysis for colocalisation of TWAS-significant loci in lung (GTEXv8) . . 51
28 Gene-level manhattan and Q-Q plots per predicted ancestry for the *pLof* mask. . . . 56
29 Gene-level manhattan and Q-Q plots per predicted ancestry for the *missense* mask. 57
30 Robustness of HLA association results to different posterior probability call thresholds for HIBAG.Manhattan plot of HLA allele associations across the extended MHC region with Covid-19 critical illness for the EUR cohort. Each panel corresponds to association results obtained using genotypes that were called using a different call threshold for HIBAG (0, 0.5 and 0.7, respectively). Diamonds represent the HLA each allele association, coloured by locus. The lead variant from the lead HLA allele is labelled. The dashed red line is the Bonferroni-corrected genome-wide significance threshold for Europeans. 60
31 Meta-analysis by information content (MAIC) dataset interaction plot 63

List of Tables

1 Description of the cohorts included in this study 5
2 Description of the cohorts included in this study by predicted ancestry, after sample QC and removal of related individuals. 5
3 Characteristics and comorbidity of the Covid-19 severe cohort (n=7,491). 14
4 Age and BMI for each cohort 15
5 Bonferoni-corrected P-values for the per-population GWAS analyses. 16
6 Individual-level conditional analysis using SAIGE (EUR) 19
7 Effect size and P-value comparison with our initial report from microarray and imputation data from the GenOMICC study, Pairo-Castineira et al (2020).[1] 20
8 Replication in combined data from external studies 24
9 Fine mapping results (EUR) 30
10 Allele frequency comparison and GWAS results for lead variants of the study 41
11 Heritability estimates 42
12 Lead signals with genes where is evidence of gene expression affecting disease severity, found by TWAS and colocalisation analysis. 52
13 Exposures and instruments for the 16 significant protein in GSMR analysis 54
14 Number of tested genes per mask per predicted ancestry 55
15 Pathway enrichment results 62

Cohort description

Pre-QC description

Cohort	Instrument	Alignment & variant calling workflow	N (pre-QC)	Included in aggregate
covid - severe	NovaSeq	Genomics England pipeline 2.0	8,794	aggCOVID_v4.2
covid - mild	NovaSeq	Genomics England pipeline 2.0	1,809	aggCOVID_v4.2
100K-Genomes(not realigned)	Hiseq X	Illumina North Star Version 4 (NSV4, version 2.6.53.23)	72,060	aggV2
100K-Genomes(realigned)	Hiseq X	Genomics England pipeline 2.0	4,183	aggCOVID_v4.2

Supplementary Table 1: Description of the cohorts included in this study

Post-QC description

Predicted Ancestry	*covid-severe*	*covid-mild*	*100K-Genomes (not realigned)*	*100K-Genomes (realigned)*
EUR	5,989	1,507	38,325	3,059
SAS	788	95	3379	319
AFR	440	14	1025	311
EAS	274	14	280	72

Supplementary Table 2: Description of the cohorts included in this study by predicted ancestry, after sample QC and removal of related individuals.

SampleQC

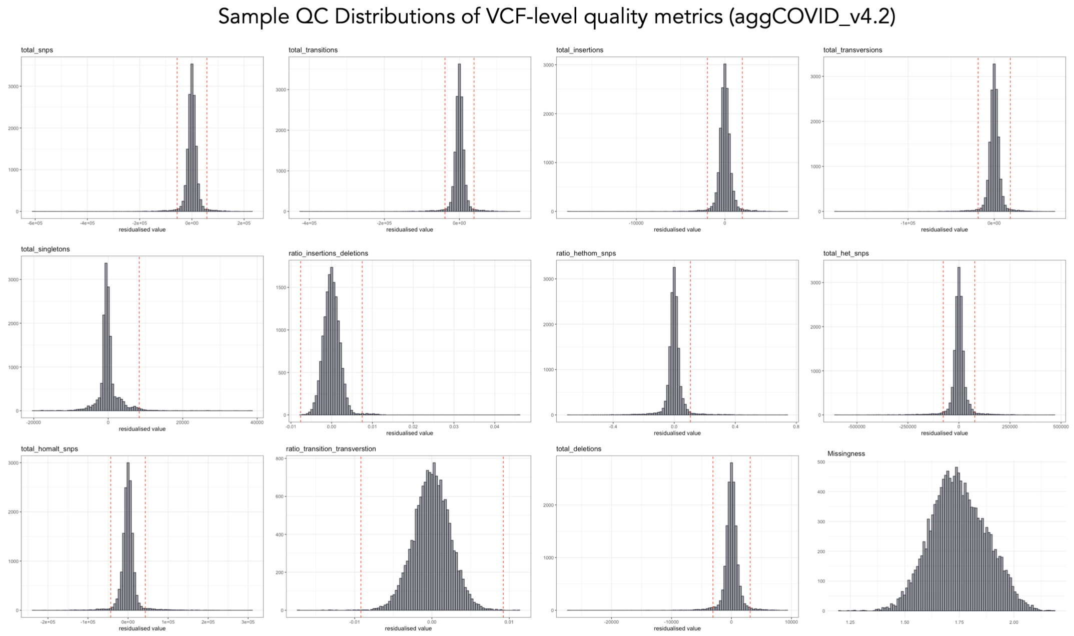

Supplementary Figure 1: Each histogram shows the distribution of samples in the aggCOVID v4.2 data-set for a particular VCF-level quality metric, following adjustment for sequencing platform and the first three ancestry assignment principal components (as described in Methods). All metrics are calculated from autosomal bi-allelic SNVs. The dashed read lines indicate the threshold for sample exclusion. Samples were removed that were four median absolute deviations (MADs) above or below the median for the following metrics: ratio heterozygous-homozygous, ratio insertions-deletions, ratio transitions-transversions, total deletions, total insertions, total heterozygous snps, total homozygous snps, total transitions, total transversions. For the number of total singletons (snps), samples were removed that were more than 8 MADs above the median. For the ratio of heterozygous to homozygous alternate snps, samples were removed that were more than 4 MADs above the median. For sample-missingness (bottom-right panel), a hard cut-off of 0.05 was applied (no adjustment for sequencing platform or ancestry).

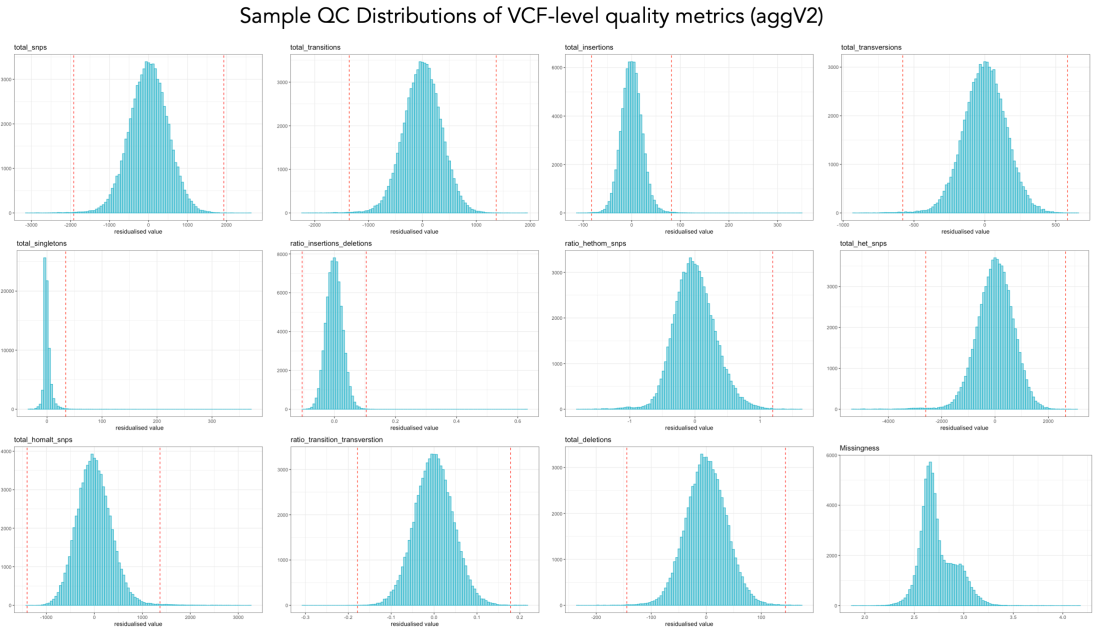

Supplementary Figure 2: Each histogram shows the distribution of samples in the aggV2 data-set for a particular VCF-level quality metric, following adjustment for sequencing platform and the first three ancestry assignment principal components (as described in Methods). All metrics are calculated from autosomal bi-allelic SNVs. The dashed read lines indicate the threshold for sample exclusion. Samples were removed that were four median absolute deviations (MADs) above or below the median for the following metrics: ratio heterozygous-homozygous, ratio insertions-deletions, ratio transitions-transversions, total deletions, total insertions, total heterozygous snps, total homozygous snps, total transitions, total transversions. For the number of total singletons (snps), samples were removed that were more than 8 MADs above the median. For the ratio of heterozygous to homozygous alternate snps, samples were removed that were more than 4 MADs above the median. For sample-missingness (bottom-right panel), a hard cut-off of 0.05 was applied (no adjustment for sequencing platform or ancestry).

PCA and ancestry

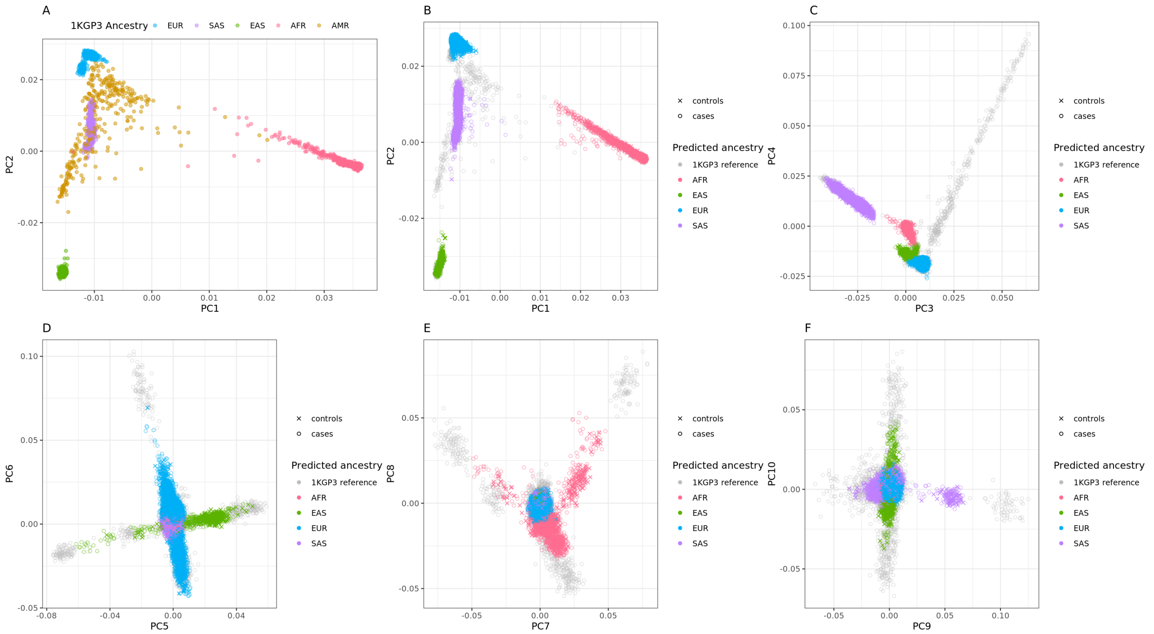

Supplementary Figure 3: Projection of severe, mild and 100K individuals onto the PCs of the 1000 genomes project phase 3 individuals (1KGP3). (A) PCs 1 & 2 for 1KGP3 unrelated individuals using the high quality independent SNP set. (B-F). Projected PCs 1-10 for severe (cases) and mild + 100K individuals (controls). 1KGP3 reference individuals are shown in grey (as background). Note that for panels B-F, the displayed colored populations had inferred genetic ancestry as EUR, SAS, AFR and EAS and were analysed in this study.

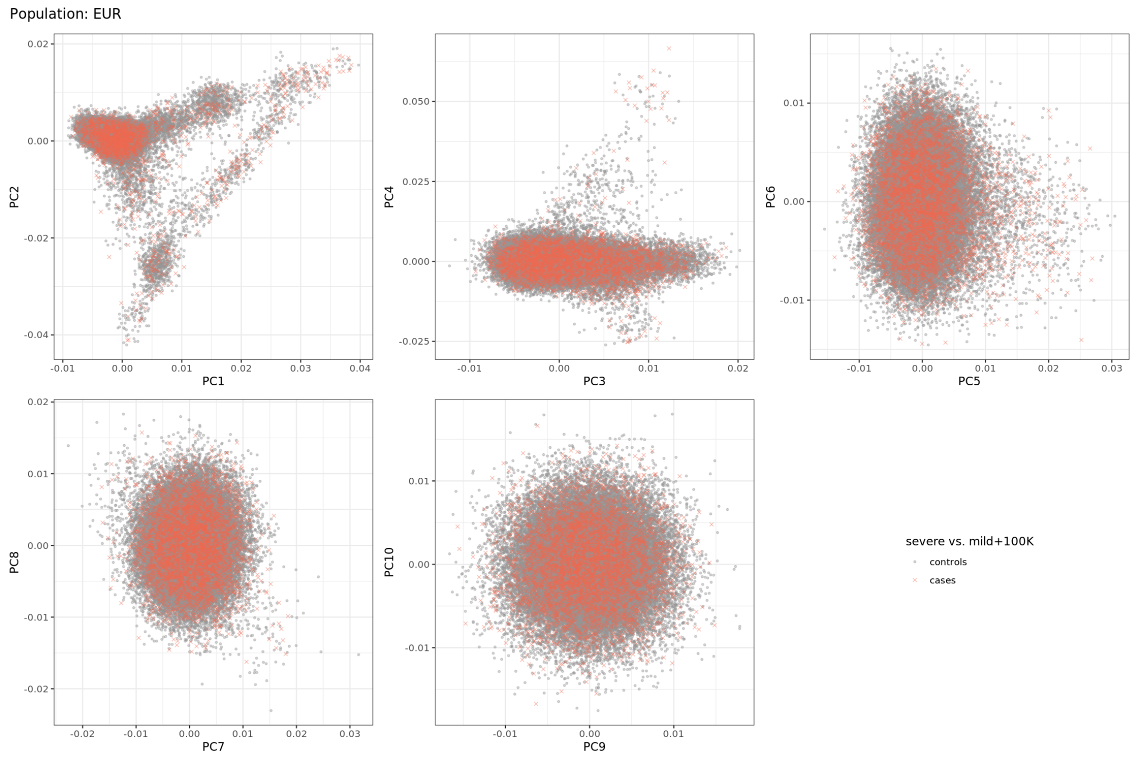

Supplementary Figure 4: EUR PCs 1-10 for severe (cases) and mild + 100K individuals (controls).

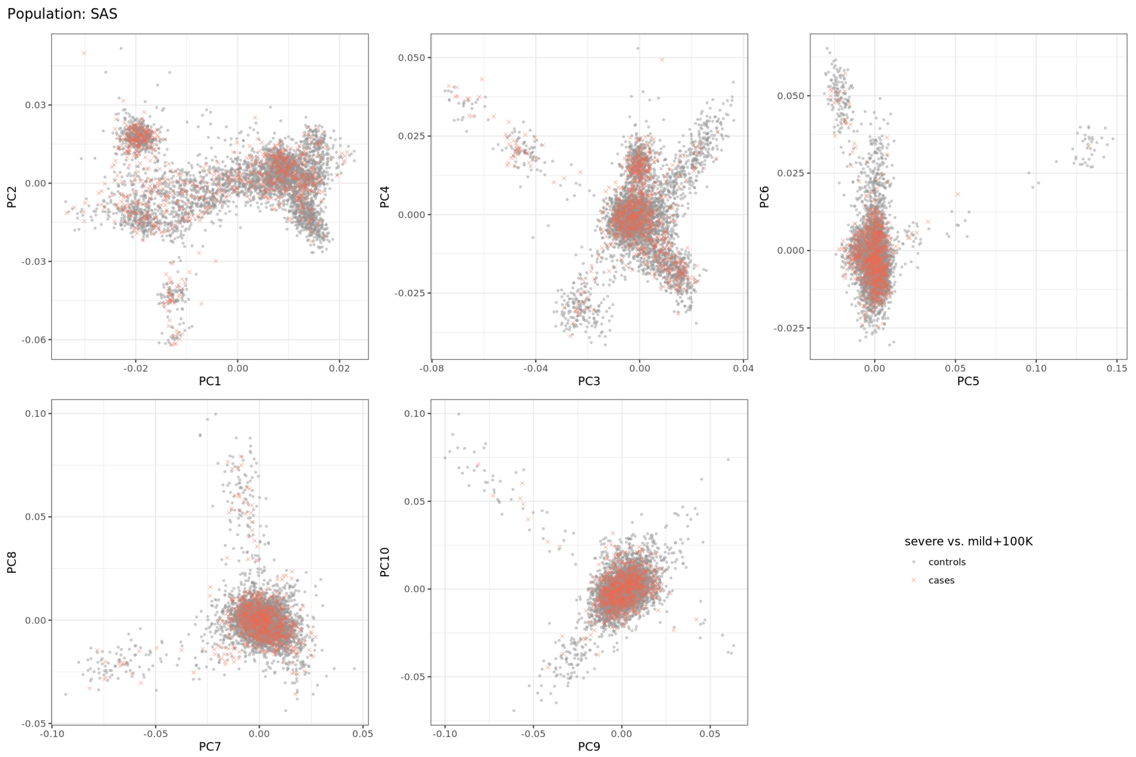

Supplementary Figure 5: SAS PCs 1-10 for severe (cases) and mild + 100K individuals (controls).

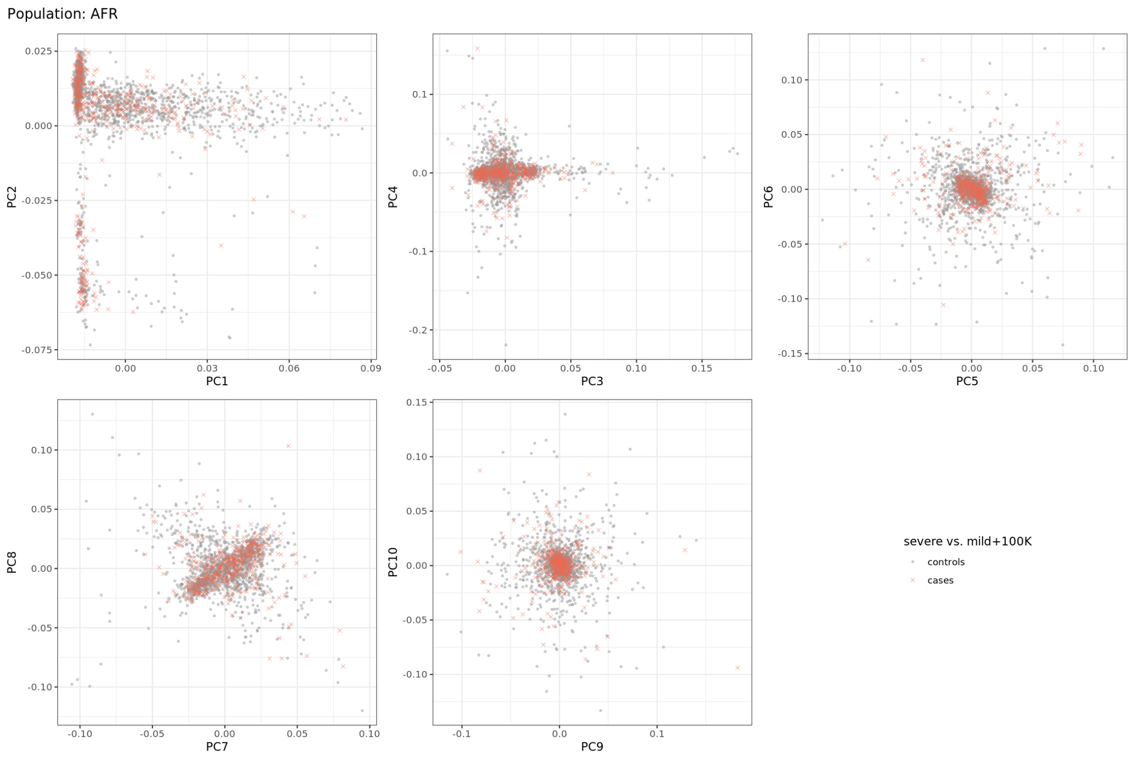

Supplementary Figure 6: AFR PCs 1-10 for severe (cases) and mild + 100K individuals (controls).

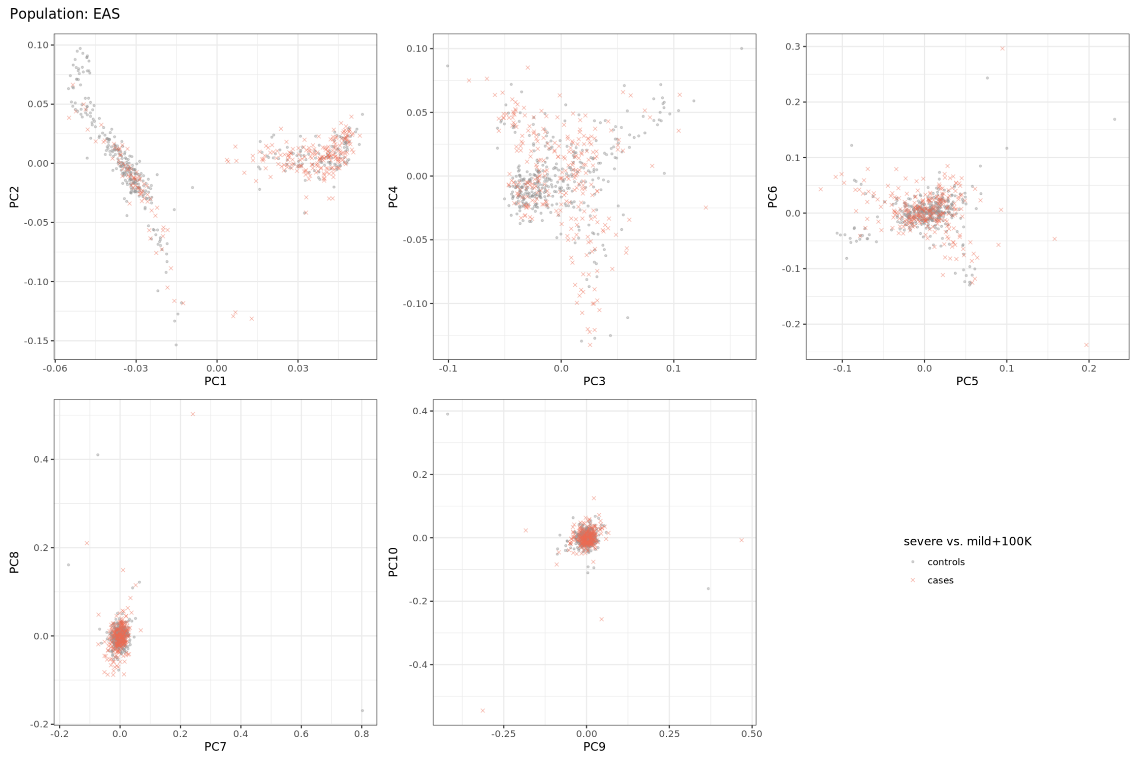

Supplementary Figure 7: EAS PCs 1-10 for severe (cases) and mild + 100K individuals (controls).

Cohort characteristics

Disease characteristics

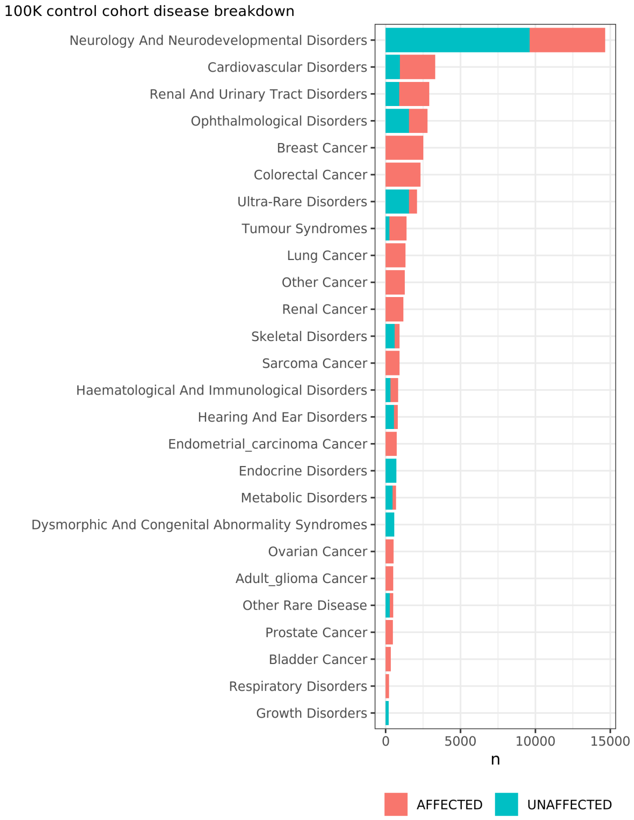

Supplementary Figure 8: Disease characteristics for the 100K controls that were used in this study after QC filters. The 100,000 Genomes Project includes participants with rare disorders and their family members, and participants with a range of different cancer types. For the control population used in this study, unrelated participants were selected from the 100,000 Genomes Project cohort (n=46,770), including a total of 34,621 rare disease participants of which 18,915 were unaffected family members of rare disease participants, 14,701 were affected rare disease participants (not related to the unaffected family members selected), 1,005 were rare disease participants not assessed for affection status and 12,149 were cancer participants.

Covid-19 severe characteristics	Yes	No	Missing
Significant comorbidity	1605	5873	13
Invasive ventilation	4028	3461	2
Died (60 days)	2154	5203	134

Supplementary Table 3: Characteristics and comorbidity of the Covid-19 severe cohort (n=7,491).

Demographics

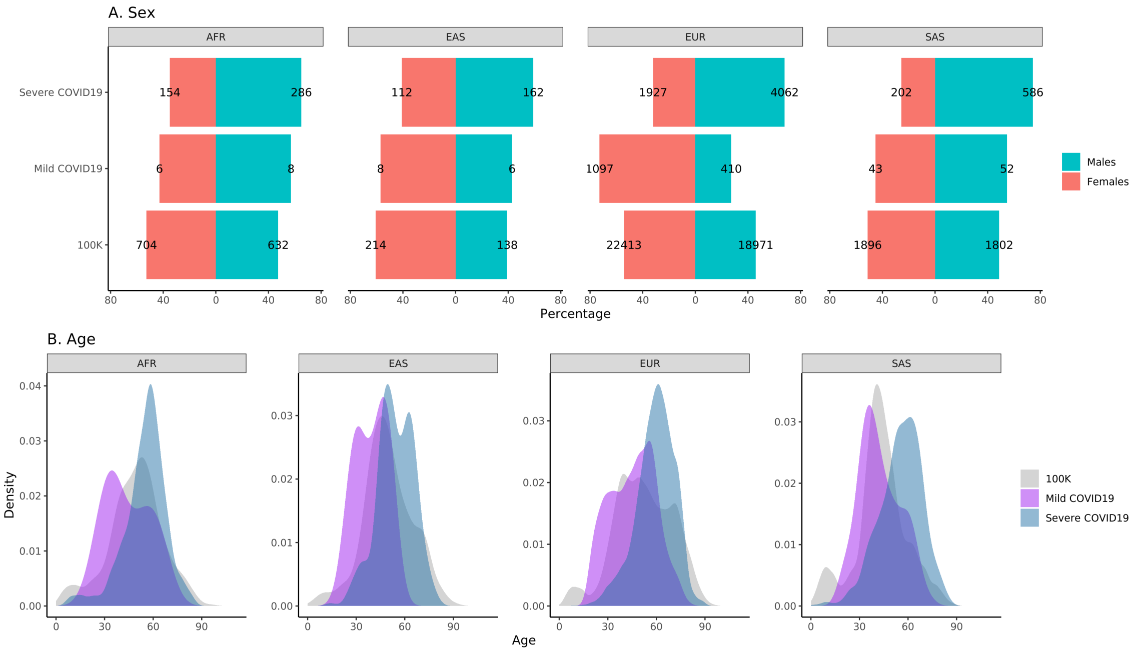

Supplementary Figure 9: Demographic characteristics of Covid-19 severe, Covid-19 mild and 100K cohorts. Panel A displays the numeric breakdown into males and females across the different cohorts. Panel B displays the age distribution across cohorts.

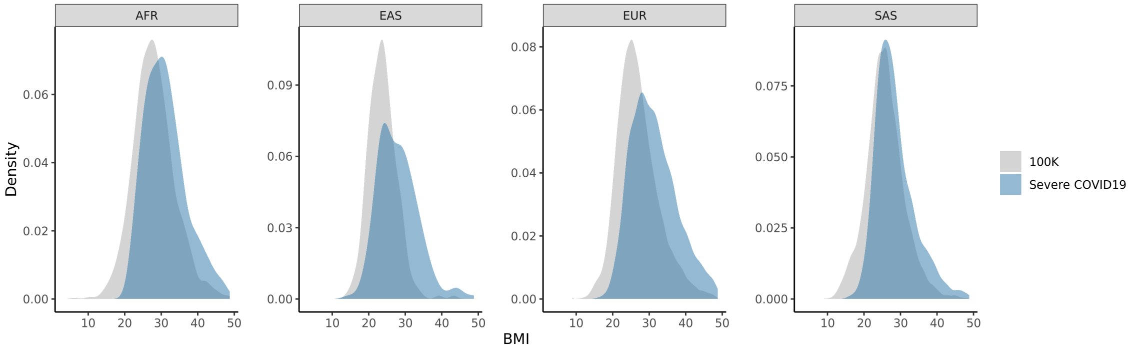

Supplementary Figure 10: Body mass index (BMI) for a subset of severe cases and controls of this study. Data is shown for a subset of 35,732 100K controls and 4,852 severe Covid-19 cases with available BMI data. Numeric counts by genetic ancestry AFR, EAS, EUR and SAS was 1043, 274, 31371, 3044 for 100K and 265, 168, 3864, 555 for severe Covid-19.

Metric	100K	Mild COVID-19	Severe COVID-19
n_{age}	46,770	1,630	7,491
age	51[26]	46[22]	60[15]
n_{BMI}	35,732	-	4,852
BMI	26.1[6.88]	-	29.9[8.48]

Supplementary Table 4: Age and BMI for each cohort analysed in this study. The sample sizes for calculating each metric are given (n_{age} and n_{BMI}) along with the median values for age and BMI and their interquantile range in brackets.

GWAS

Per-population GWAS results

Population	No. of ld-pruned variants	Bonferroni-corrected P-value threshold
EUR	2,264,479	2.2e-08
SAS	2,729,540	1.8e-08
AFR	5,370,001	9.3e-09
EAS	1,264,431	4e-08

Supplementary Table 5: Bonferroni-corrected P-values for the per-population GWAS analyses. The P-value significance threshold (2.2×10^{-08}) was calculated by estimating the effective number of tests. After selecting the final filtered set of tested variants for each population, we LD-pruned in a window of 250Kb and $r^2 = 0.8$ with plink 1.9, which identified 2,264,479 independent linkage disequilibrium-pruned genetic variants. Results are consistent with previous modelling.[2]

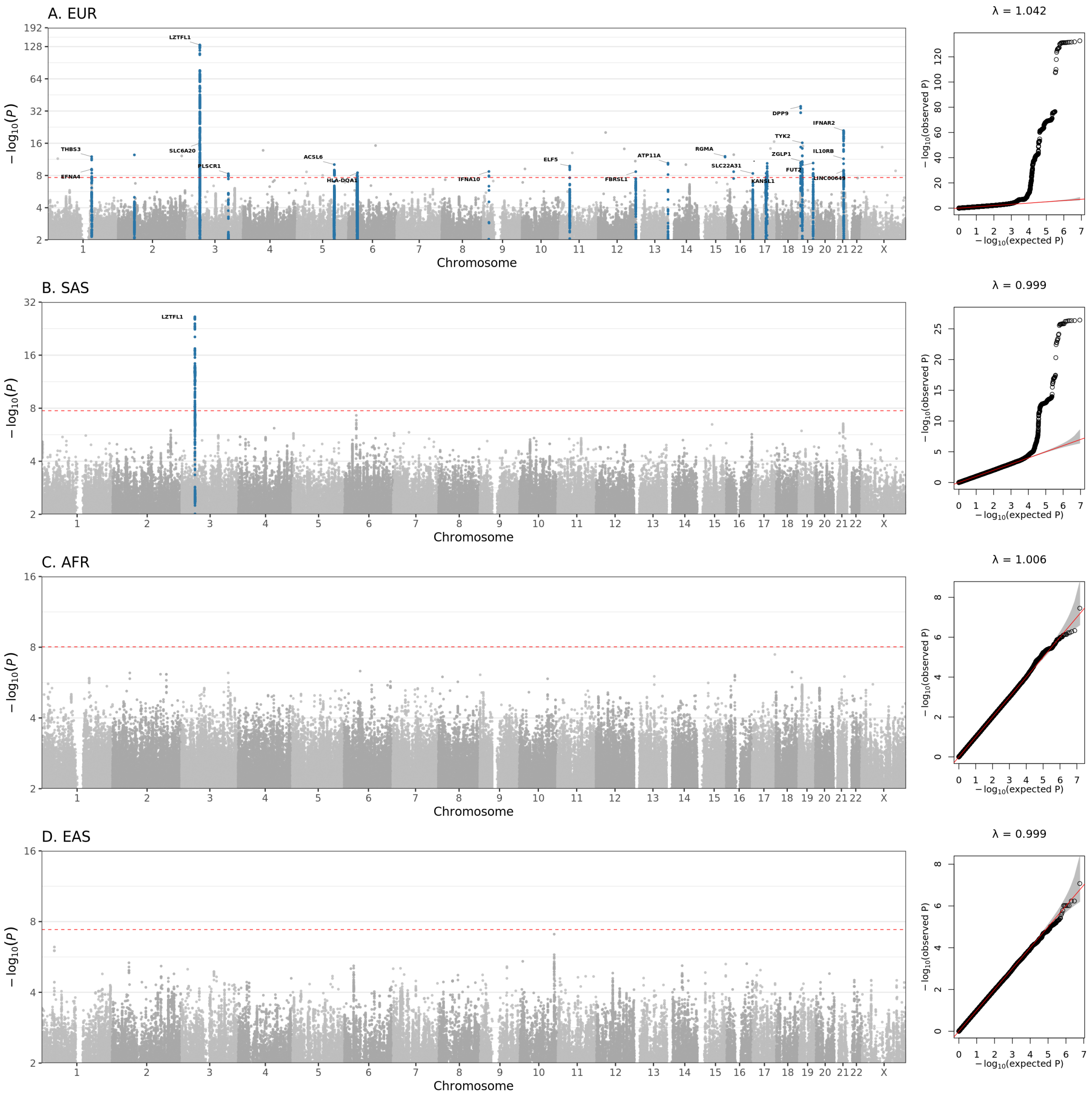

Supplementary Figure 11: Manhattan plots showing GWAS results for each population cohort (A. EUR B. SAS C. AFR D. EAS). The highlighted results with blue are the variants that are LD clumped (r^2=0.1, P_2=0.01 in each population) with each lead variant. Red dashed line is the Bonferroni-corrected P-value according to the number of estimated independent tests in each population (indicated in Supplementary Table 5).

LD-based validation of lead GWAS signals

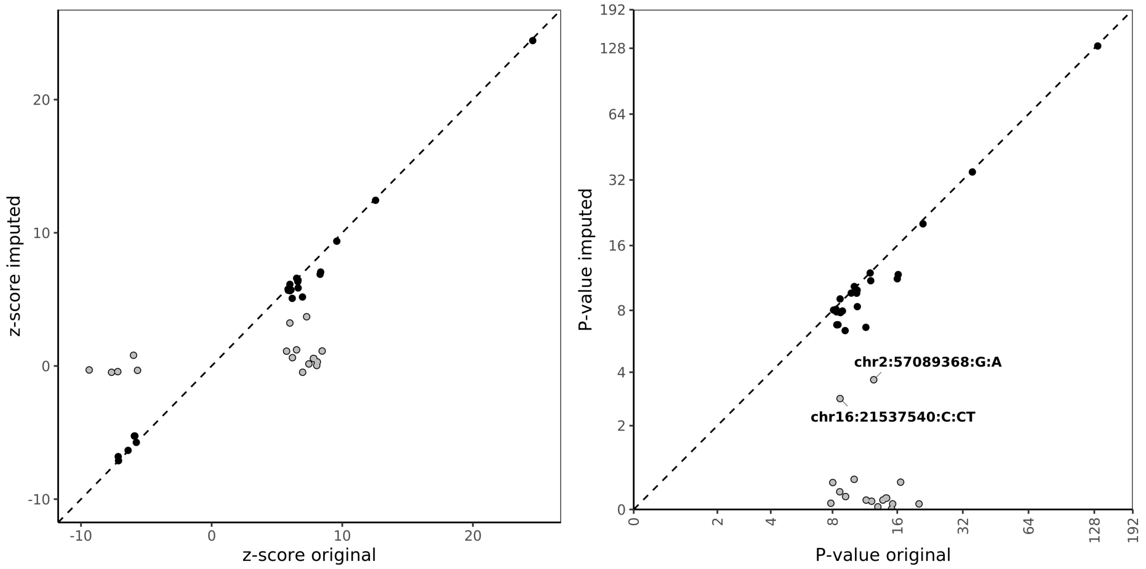

Supplementary Figure 12: Original and imputed z-scores and respective P-values with leave-one procedure for lead variants of the EUR analysis. Variants with low support from neighbouring variants are highlighted with grey.

Individual-level conditional analysis

CHR:POS$_{hg38}$:REF$_{hg38}$:ALT	rsid	condition rsid	BETA	SE	Pval	$BETA_{cond}$	SE_{cond}	$Pval_{cond}$
chr1:155066988:C:T	rs114301457	rs7528026,rs41264915	0.874	0.142	6.87E-10	0.867	0.142	9.09E-10
chr1:155175305:G:A	rs7528026	rs114301457,rs41264915	0.33	0.0593	2.6E-08	0.311	0.0593	1.62E-07
chr1:155197995:A:G	rs41264915	rs114301457,rs7528026	-0.245	0.0343	8.87E-13	-0.229	0.0343	2.41E-11
chr3:45796521:G:T	rs2271616	rs73064425,rs343320	0.253	0.0305	1.07E-16	0.324	0.0309	8.93E-26
chr3:45859597:C:T	rs73064425	rs2271616,rs343320	0.997	0.0406	4.75E-133	1.02	0.0408	4.17E-138
chr3:146517122:G:A	rs343320	rs2271616,rs73064425	0.226	0.0385	4.44E-09	0.222	0.0385	8.27E-09
chr6:32623820:T:C	rs9271609	rs2496644	-0.13	0.022	3.27E-09	-0.136	0.0221	6.38E-10
chr6:41515007:A:C	rs2496644	rs9271609	-0.296	0.0854	0.000525	-0.292	0.0854	0.000634
chr17:46152620:T:C	rs2532300	rs3848456	-0.149	0.0253	4.09E-09	-0.149	0.0253	4.28E-09
chr17:49863260:C:A	rs3848456	rs2532300	0.398	0.0621	1.38E-10	0.405	0.0621	7.39E-11
chr19:4717660:A:G	rs12610495	rs73510898,rs34536443,rs368565	0.282	0.0224	2.69E-36	0.273	0.0224	5.53E-34
chr19:10305768:G:A	rs73510898	rs12610495,rs34536443,rs368565	0.244	0.0361	1.4E-11	0.258	0.0363	1.19E-12
chr19:10352442:G:C	rs34536443	rs12610495,rs73510898,rs368565	0.404	0.0485	7.87E-17	0.404	0.0486	1.03E-16
chr19:48697960:C:T	rs368565	rs12610495,rs73510898,rs34536443	0.141	0.0213	3.12E-11	0.14	0.0213	5.17E-11
chr21:33230000:C:A	rs17860115	rs8178521,rs35370143	0.217	0.0225	6.47E-22	0.195	0.0227	1.15E-17
chr21:33287378:C:T	rs8178521	rs17860115,rs35370143	0.163	0.0236	4.41E-12	0.139	0.0238	4.39E-09
chr21:33959662:T:TAC	rs35370143	rs17860115,rs8178521	0.23	0.038	1.31E-09	0.228	0.038	1.84E-09

Supplementary Table 6: Results from individual-level conditional analysis using SAIGE for EUR population for cases where multiple association signals reside in the same chromosome. Effect size estimates (BETA) and it's standard error (SE) and P-values along with the estimates when conditioning on other genome-wide lead signals on the same chromosome (condition rsid) are shown.

Comparison to 2020 GenOMICC microarray study

chr:pos (hg38)	rsid	REF	ALT	MAF	BETA	SE	P	MAF_{2020}	$BETA_{2020}$	SE_{2020}	P_{2020}
3:45859597	rs73064425	C	T	0.0771	0.998	0.041	1.97E-133	0.083	0.763	0.067	4.77E-30
6:29831017	rs9380142	A	G	0.315	-0.080	0.022	0.000377	0.302	-0.263	0.047	3.23E-08
6:31153649	rs143334143	G	A	0.068	0.101	0.042	0.0147	0.079	0.615	0.072	8.82E-18
6:32212369	rs3131294	A	G	0.136	-0.085	0.030	0.00495	0.140	-0.118	0.062	0.058
12:112942203	rs10735079	G	A	0.359	0.072	0.022	0.000981	0.361	0.258	0.046	1.65E-08
19:4719431	rs2109069	G	A	0.331	0.257	0.022	1.38E-31	0.328	0.306	0.044	3.98E-12
19:10317045	rs74956615	T	A	NA	NA	NA	NA	0.059	0.462	0.083	2.31E-08
21:33252612	rs2236757	A	G	0.288	-0.205	0.023	7.78E-19	0.291	-0.251	0.046	5.00E-08

Supplementary Table 7: Effect size and P-value comparison with our initial report from microarray and imputation data from the GenOMICC study, Pairo-Castineira et al (2020).[1]

Association signal forest plots by genetic ancestry

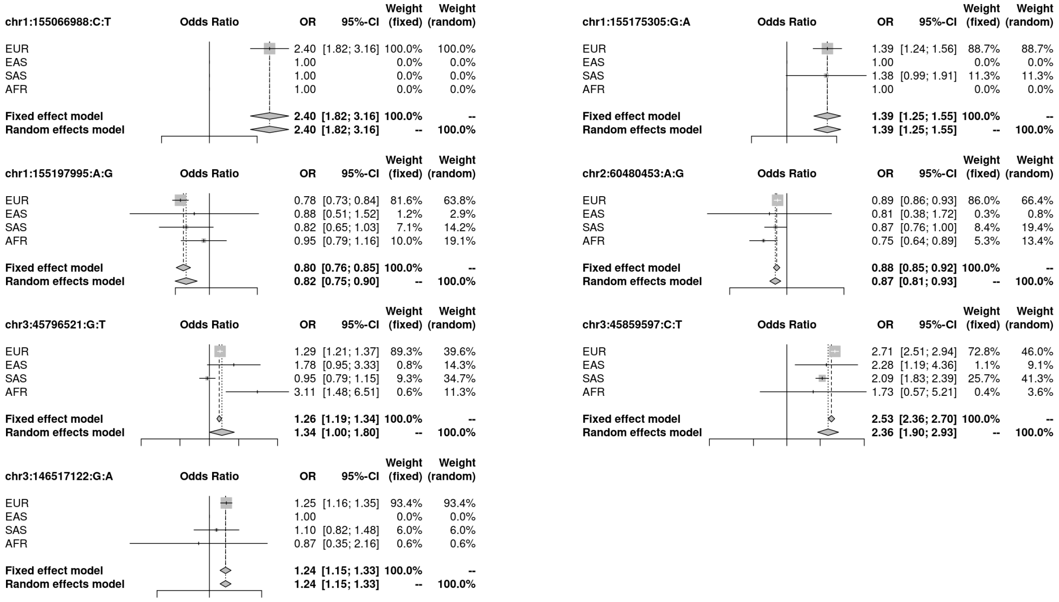

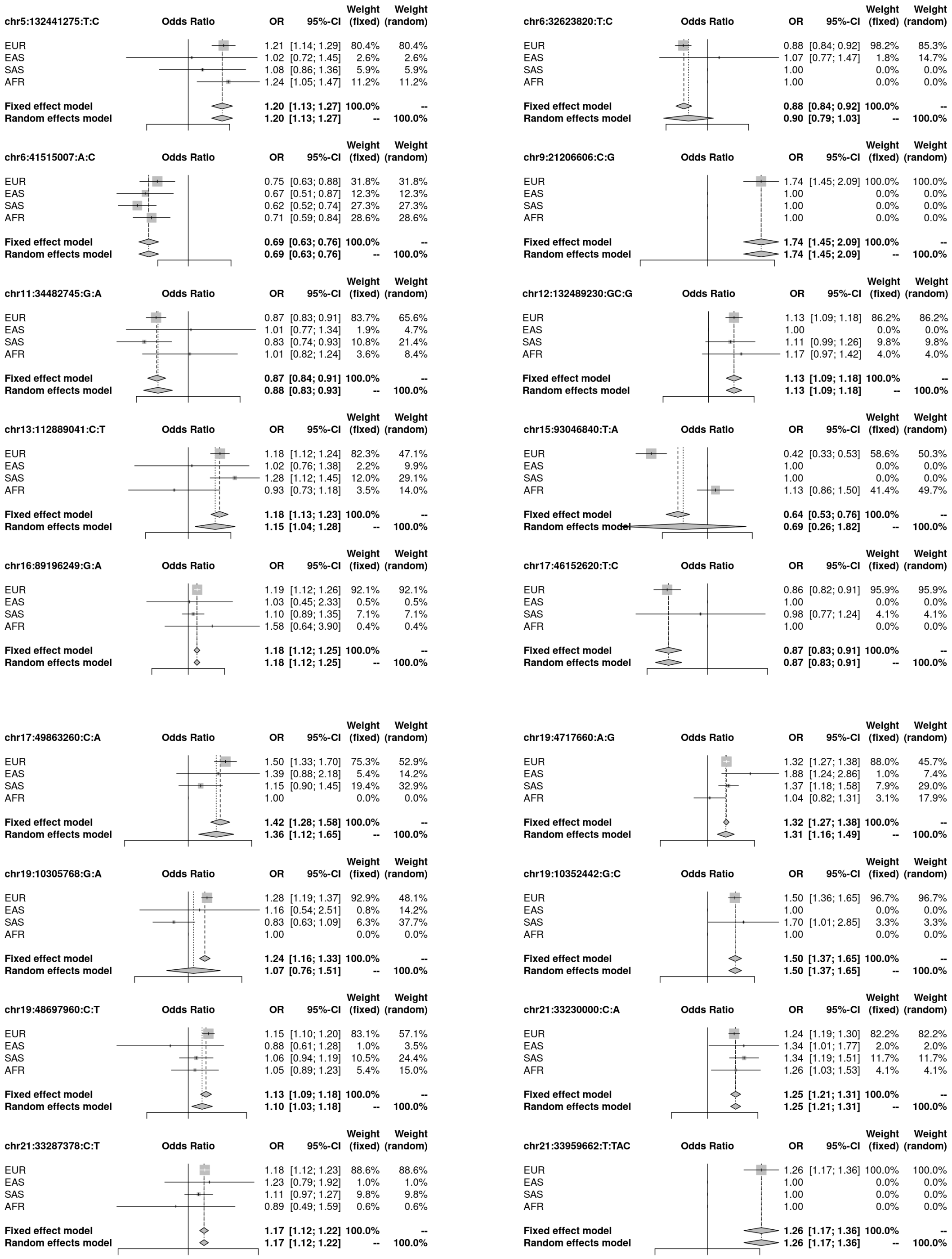

Supplementary Figure 13: Forest plots by genetic ancestry for all lead signals.

Replication

chr:pos (b38)	rsid	REF	ALT	HetPVal	AF_{cases}	$AF_{controls}$	OR	OR_{CI}	P	$OR_{hgib2.23m}$	$OR_{CIhgib2.23m}$	$P_{hgib2.23m}$	$OR_{hgia2.23msev}$	$OR_{CIhgia2.23msev}$	$P_{hgia2.23msev}$	OR_{reg}	$OR_{CI.reg}$	P_{reg}	Gene
1:155066988	rs114301457	C	T	1	0.010	0.0052	2.40	1.81-3.18	1.51×10^{-9}	1.46	1.21-1.77	0.00011 *	-	-	-	-	-	-	*EFNA4*
1:155175305	rs7528026	G	A	0.96	0.041	0.031	1.39	1.24-1.55	7.16×10^{-9}	1.14	1.07-1.22	0.00012 *	1.29	1.15-1.46	3.68×10^{-5} *	-	-	-	*TRIM46*
1:155197995	rs41264915	A	G	0.29	0.087	0.11	0.80	0.76-0.85	3.79×10^{-12}	0.90	0.87-0.93	1.51×10^{-9} *	0.82	0.77-0.88	1.2×10^{-9} *	-	-	-	*THBS3*
2:60480453	rs1123573	A	G	0.29	0.36	0.39	0.88	0.85-0.92	9.85×10^{-10}	0.95	0.93-0.97	1.76×10^{-5} *	0.91	0.87-0.94	1.47×10^{-6} *	-	-	-	*BCL11A*
3:45796521	rs2271616	G	T	0.0011	0.16	0.13	1.26	1.19-1.34	2.45×10^{-15}	1.11	1.07-1.15	4.95×10^{-9} *	1.11	1.04-1.19	0.0011 *	-	-	-	*SLC6A20*
3:45859597	rs73064425	C	T	0.01	0.14	0.068	2.52	2.35-2.7	2.18×10^{-152}	1.46	1.4-1.51	1.02×10^{-77} *	1.76	1.65-1.88	1.81×10^{-63} *	-	-	-	*LZTFL1*
3:146517122	rs343320	G	A	0.53	0.094	0.079	1.24	1.15-1.33	1.52×10^{-8}	1.08	1.04-1.13	0.00028 *	1.10	1.02-1.18	0.016	-	-	-	*PLSCR1*
5:132441275	rs10066378	T	C	0.61	0.14	0.12	1.20	1.13-1.27	4.48×10^{-10}	1.05	1.02-1.08	0.00074 *	1.06	1.01-1.11	0.027	-	-	-	*IRF1-AS1*
6:32623820	rs9271609	T	C	0.24	0.32	0.35	0.88	0.84-0.92	1.27×10^{-8}	1.00	0.98-1.03	0.89	1.01	0.96-1.06	0.844	-	-	-	*HLA-DQA1*
6:41515007	rs2496644	A	C	0.49	0.98	0.99	0.69	0.63-0.76	7.59×10^{-15}	0.87	0.83-0.92	3.17×10^{-7} *	-	-	-	-	-	-	*LINC01276*
9:21206606	rs28368148	C	G	1	0.019	0.012	1.74	1.45-2.1	4.09×10^{-9}	1.21	1.07-1.37	0.0024	1.26	0.69-2.3	0.46	1.29	1.11-1.49	0.00089 *	*IFNA10*
11:34482745	rs61882275	G	A	0.29	0.35	0.38	0.87	0.84-0.91	1.62×10^{-11}	0.93	0.91-0.95	1.9×10^{-10} *	0.91	0.87-0.95	3.24×10^{-6} *	-	-	-	*ELF5*
12:132479205	rs4883585	G	A	0.9	0.52	0.49	1.13	1.09-1.18	1.12×10^{-9}	1.04	1.02-1.06	0.00047 *	1.08	1.04-1.12	0.00026 *	-	-	-	*FBRSL1*
13:112889041	rs9577175	C	T	0.1	0.25	0.22	1.18	1.13-1.23	1.61×10^{-12}	1.07	1.04-1.09	1.29×10^{-6} *	1.11	1.06-1.16	3.18×10^{-6} *	-	-	-	*ATP11A*
15:93046840	rs4424872	T	A	1.82×10^{-7}	0.986	0.992	0.64	0.53-0.77	1.99×10^{-6}	-	-	-	-	-	-	0.91	0.78-1.07	0.29	*RGMA*
16:89196249	rs117169628	G	A	0.8	0.17	0.14	1.18	1.12-1.25	6.04×10^{-9}	1.10	1.07-1.14	6.57×10^{-9} *	1.18	1.11-1.24	2.65×10^{-8} *	-	-	-	*SLC22A31*
17:46152620	rs2532300	T	C	0.32	0.20	0.23	0.87	0.82-0.91	1.4×10^{-8}	0.92	0.89-0.95	2.49×10^{-9} *	0.89	0.85-0.93	3.11×10^{-6} *	-	-	-	*KANSL1*
17:49863260	rs3848456	C	A	0.14	0.040	0.028	1.42	1.27-1.58	1.47×10^{-10}	1.15	1.09-1.21	1.34×10^{-7} *	1.21	1.1-1.34	0.00015 *	-	-	-	.
19:4717660	rs12610495	A	G	0.069	0.36	0.30	1.32	1.27-1.38	6.44×10^{-39}	1.11	1.09-1.14	5.74×10^{-19} *	1.19	1.14-1.25	6.49×10^{-16} *	-	-	-	*DPP9*
19:10305768	rs73510898	G	A	0.011	0.110	0.0910	1.24	1.16-1.33	1.47×10^{-9}	1.08	1.04-1.12	0.00016 *	1.21	1.12-1.3	2.59×10^{-7} *	-	-	-	*ZGLP1*
19:10352442	rs34536443	G	C	0.64	0.066	0.0480	1.50	1.37-1.66	4.22×10^{-17}	1.22	1.15-1.29	4.06×10^{-11} *	1.49	1.33-1.66	7.95×10^{-13} *	-	-	-	*TYK2*
19:48697960	rs368565	C	T	0.22	0.470	0.4400	1.13	1.09-1.18	3.74×10^{-10}	1.04	1.02-1.06	0.00087 *	1.07	1.03-1.12	0.00067 *	-	-	-	*FUT2*
21:33230000	rs17860115	C	A	0.62	0.36	0.31	1.26	1.21-1.31	6.28×10^{-28}	1.11	1.08-1.13	1.77×10^{-18} *	1.15	1.11-1.2	1.71×10^{-11} *	-	-	-	*IFNAR2*
21:33287378	rs8178521	C	T	0.67	0.29	0.27	1.17	1.12-1.22	4.23×10^{-12}	1.06	1.03-1.09	8.02×10^{-6} *	1.10	1.05-1.15	4.19×10^{-5} *	-	-	-	*IL10RB*
21:33914436	rs12626438	A	G	1	0.098	0.0810	1.22	1.14-1.31	1.78×10^{-8}	1.10	1.06-1.14	2.33×10^{-7} *	1.12	1.06-1.2	0.0002 *	-	-	-	*LINC00649*

Supplementary Table 8: Replication in combined data from external studies. hgib2.23m: combined meta-analysis of HGI freeze 6 B2 and 23andMe hospitalised phenotype, hgia2.23msev: combined meta-analysis of HGI A2 and 23andMe severe phenotype, reg: additional GWAS meta-analysis (UKB, AncestryDNA, PMBB, GHS) . Odds ratios and P-values are shown for variants in LD with the lead variant that were genotyped/imputed in replication data. Chromosome, reference and alternate allele correspond to the build hg38; allele frequency of the alternate allele is reported separately for cases and controls. HetPVal corresponds to the heterogeneity P-value for the multi-ancestry meta-analysis. An asterisk (*) next to the hosp, sev, or reg P-value indicates that the lead signal is replicated with a Bonferroni-corrected $P < 0.002$ (0.05/25) and with a concordant direction of effect.

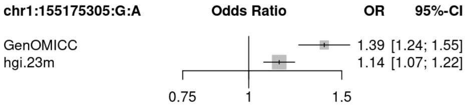
chr1:155175305:G:A
Odds Ratio
OR
95%-CI
GenOMICC
hgi.23m
1.39 [1.24; 1.55]
1.14 [1.07; 1.22]
0.75
1
1.5

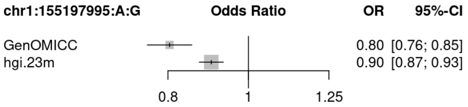
chr1:155197995:A:G
Odds Ratio
OR
95%-CI
GenOMICC
hgi.23m
0.80 [0.76; 0.85]
0.90 [0.87; 0.93]
0.8
1
1.25

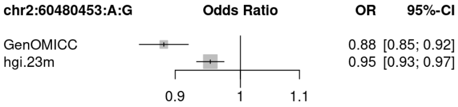
chr2:60480453:A:G
Odds Ratio
OR
95%-CI
GenOMICC
hgi.23m
0.88 [0.85; 0.92]
0.95 [0.93; 0.97]
0.9
1
1.1

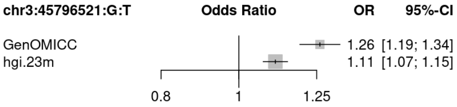
chr3:45796521:G:T
Odds Ratio
OR
95%-CI
GenOMICC
hgi.23m
1.26 [1.19; 1.34]
1.11 [1.07; 1.15]
0.8
1
1.25

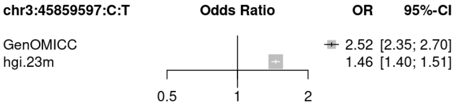
chr3:45859597:C:T
Odds Ratio
OR
95%-CI
GenOMICC
hgi.23m
2.52 [2.35; 2.70]
1.46 [1.40; 1.51]
0.5
1
2

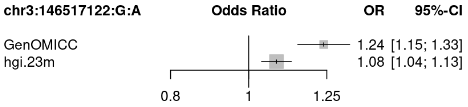
chr3:146517122:G:A
Odds Ratio
OR
95%-CI
GenOMICC
hgi.23m
1.24 [1.15; 1.33]
1.08 [1.04; 1.13]
0.8
1
1.25

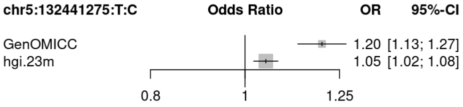
chr5:132441275:T:C
Odds Ratio
OR
95%-CI
GenOMICC
hgi.23m
1.20 [1.13; 1.27]
1.05 [1.02; 1.08]
0.8
1
1.25

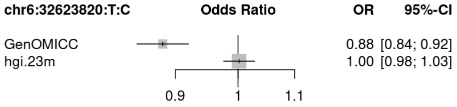
chr6:32623820:T:C
Odds Ratio
OR
95%-CI
GenOMICC
hgi.23m
0.88 [0.84; 0.92]
1.00 [0.98; 1.03]
0.9
1
1.1

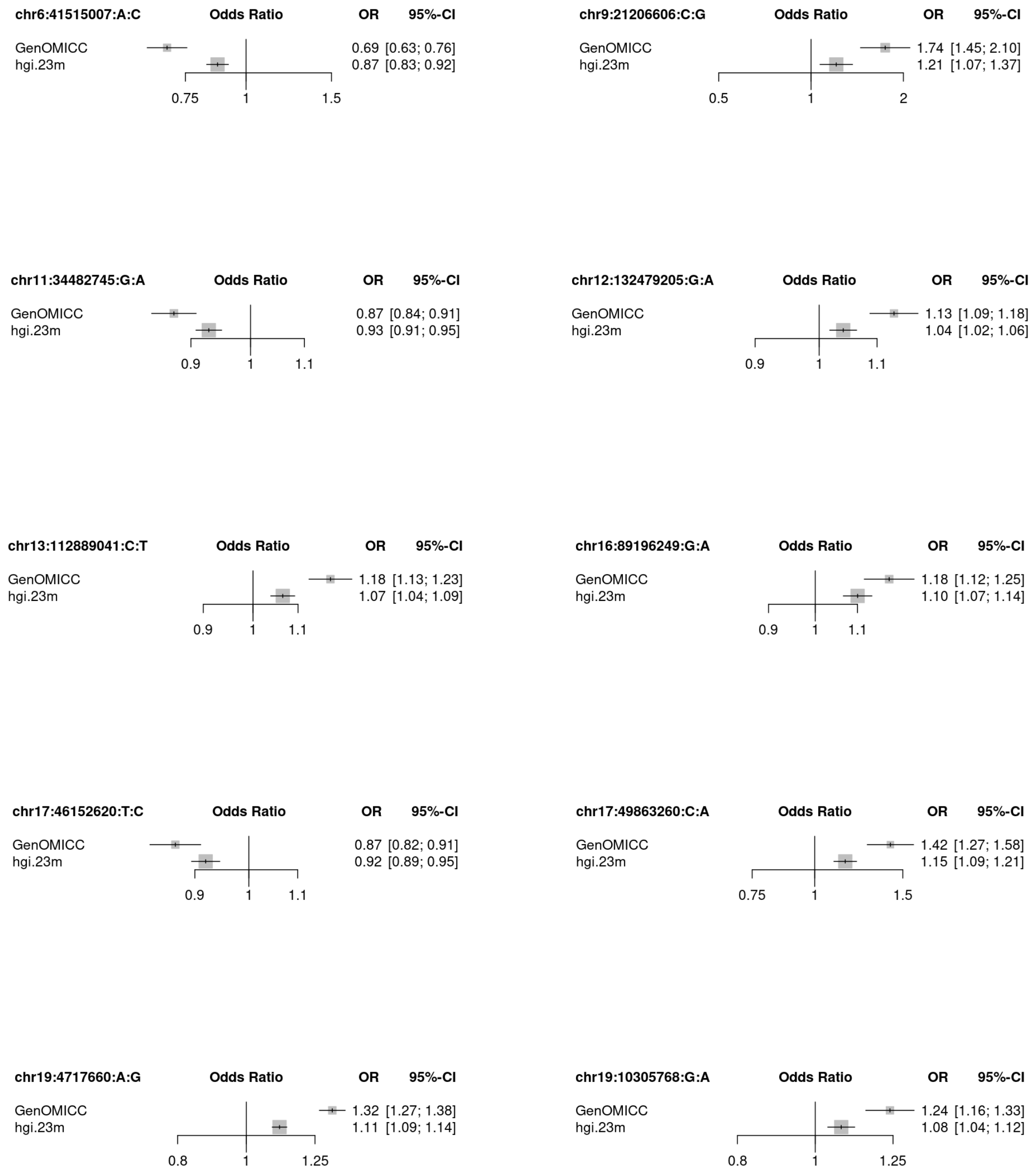
chr6:41515007:A:C
Odds Ratio
OR
95%-CI
GenOMICC
0.69 [0.63; 0.76]
hgi.23m
0.87 [0.83; 0.92]
0.75
1
1.5
chr9:21206606:C:G
Odds Ratio
OR
95%-CI
GenOMICC
1.74 [1.45; 2.10]
hgi.23m
1.21 [1.07; 1.37]
0.5
1
2
chr11:34482745:G:A
Odds Ratio
OR
95%-CI
GenOMICC
0.87 [0.84; 0.91]
hgi.23m
0.93 [0.91; 0.95]
0.9
1
1.1
chr12:132479205:G:A
Odds Ratio
OR
95%-CI
GenOMICC
1.13 [1.09; 1.18]
hgi.23m
1.04 [1.02; 1.06]
0.9
1
1.1
chr13:112889041:C:T
Odds Ratio
OR
95%-CI
GenOMICC
1.18 [1.13; 1.23]
hgi.23m
1.07 [1.04; 1.09]
0.9
1
1.1
chr16:89196249:G:A
Odds Ratio
OR
95%-CI
GenOMICC
1.18 [1.12; 1.25]
hgi.23m
1.10 [1.07; 1.14]
0.9
1
1.1
chr17:46152620:T:C
Odds Ratio
OR
95%-CI
GenOMICC
0.87 [0.82; 0.91]
hgi.23m
0.92 [0.89; 0.95]
0.9
1
1.1
chr17:49863260:C:A
Odds Ratio
OR
95%-CI
GenOMICC
1.42 [1.27; 1.58]
hgi.23m
1.15 [1.09; 1.21]
0.75
1
1.5
chr19:4717660:A:G
Odds Ratio
OR
95%-CI
GenOMICC
1.32 [1.27; 1.38]
hgi.23m
1.11 [1.09; 1.14]
0.8
1
1.25
chr19:10305768:G:A
Odds Ratio
OR
95%-CI
GenOMICC
1.24 [1.16; 1.33]
hgi.23m
1.08 [1.04; 1.12]
0.8
1
1.25

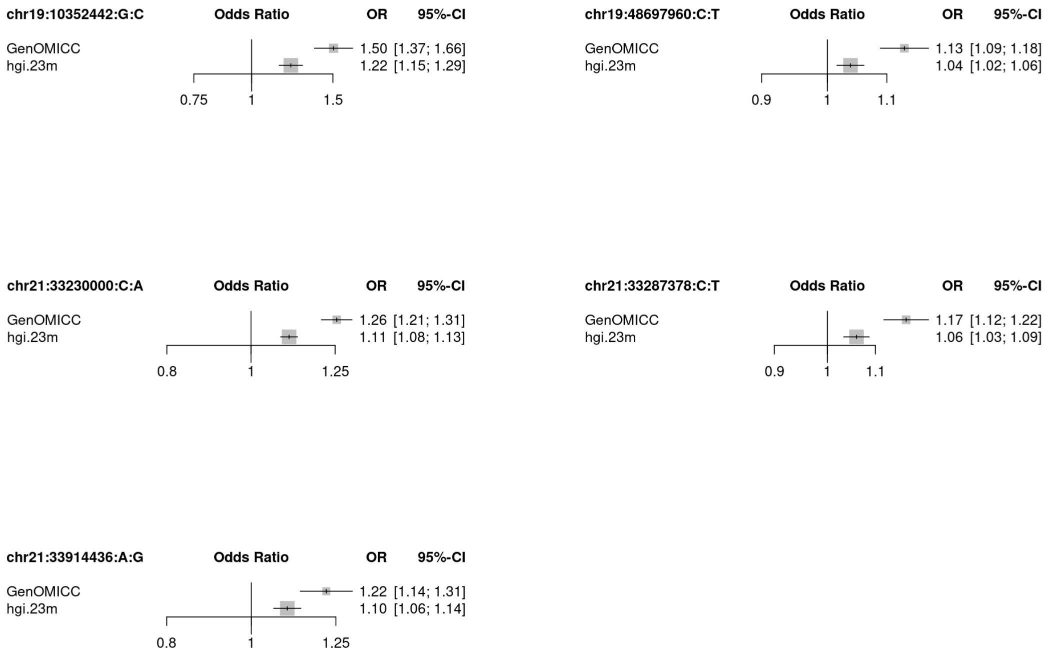

Supplementary Figure 14: Forest plots comparing the Odds ratios for all the lead signals with a combined meta-analysis of HGI freeze 6 B2 and 23andMe. The HGI summaries were produced with new meta-analysis that removed the GenOMICC cases to ensure statistical independence (n=22,598). The Genomics England 100K participants (i.e, controls) summary data contributed to HGI C2 analysis and not B2 used here.

Genetic fine-mapping

Fine-mapping analysis for lead variants

In the EUR ancestry group, we found two independent signals for the association at 1q22. The lead variant for the first fine-mapped locus is a synonymous variant in *EFNA4* (chr1:155066988:C:T), while the lead variant of the second independent signal (chr1:155197995:A:G) is an intronic variant in *THSB3*, in close proximity to the most significant single-tissue eQTLs for *MUC1* (chr1:155199564:G:T, chr1:155199139:G:A) in GTEx v8 , which are also in the same intronic region. Fine mapping the multi-ancestry meta-analysis revealed a third independent signal at this locus, with the lead variant (chr1:155175305:G:A, rs7528026, OR:1.39, 95% CIs:[1.24-1.55]) being in an intron of *TRIM46*. This variant is an sQTL and eQTL for *MUC1* in lung and and whole blood tissue, respectively, in GTEx v8[3] (Supplementary File TWAS.xlsx).

Meta-analysis across genetically inferred ancestries revealed a novel locus at 2p16.1, with the lead variant (chr2:60480453:A:G, OR:0.88, 95 %CIs:[0.85,0.92]) being in an intron of *BCL11A*.

We fine-mapped the signal in the 3p21.31 region, first reported by Ellighaus *et al*,[4] into two independent associations. The lead variant for the first association is in the 5' UTR region of *SLC6A20* (chr3:45796521:G:T, OR:1.29, 95%CIs:[1.21,1.37]). The second association in the chr3p21.31 region is seen at genome-wide significance in both the EUR and SAS cohorts, with two of the three highest ranked variants in the fine-mapped region in the two populations shared between the two cohorts (chr3:45818159:G:A, chr3:45859597:C:T) and residing in downstream and intronic regions of *LZTFL1*.

The credible set for the 3q24 association included 9 variant and the lead variant (chr3:146517122:G:A, rs343320,p.His262Tyr, OR:1.24, 95%CIs [1.15-1.33]) is a missense variant in PLSCR1, predicted to be damaging by CADD (CADD:22.6).

At 5q31.1, the lead variant (chr5:131995059:C:T, rs56162149, OR:1.17, 95%CIs:[1.11,1.23]) is in an intron of *ACSL6*. The credible set for this locus contains 33 variants that span 484 kb including variants in genes *CSF2* and *IRF1-AS1*, with chr5:132075767:T:C being a missense variant in *CSF2* and chr5:131991772:C:G being missense in *ACSL6* and only intronic variants for *IRF1-AS1*.

The previously reported signal at 6p21.1, linked to *FOXP4*[5], is stronger in the SAS cohort but has a consistent effect across ancestries (P_{het}=0.49).

We fine mapped the signal at 9p21.3 to three variants with lead variant (chr9:21206606:C:G, rs28368148,p.Trp164Cys, OR:1.74, 95% CIs [1.45-2.09]) being a missense variant in IFNA10 that is predicted to be damaging by CADD (CADD:23.9) with potential functional impact.

The signal in the 11p13 region was fine-mapped to four variants (lead variant chr11:34482745:G:A, rs61882275, OR:0.87, 95%CIs:[0.84-0.91]), all four of which are in an intron of *ELF5*.

The credible set for the signal in the 12q24.33 region includes 24 variants spanning 95 kb, of which the lead (chr12:132489230:GC:G, rs56106917, OR:1.13, 95% CIs:[1.09-1.18]) lies upsdtream *FBRSL1*.

The signal at 13q34 was fine-mapped to four variants, with lead variant (13:112889041:C:T, rs9577175, OR:1.18, 95%CIs [1.12-1.24]) lying downstream of *ATP11A* and upstream of *MCF2L* genes.

The association at 15q26.1 was fine mapped to two variants with lead variant (chr15:93046840:T:A, rs4424872, OR: 2.37, 95% CIs:[1.87-3.01] in an intron of RGMA. This a low frequency (allele

frequency <1%) variant that was not replicated due to lack of coverage in the available replication data and further validation in an independent dataset is recommended.

The credible set for the association with lead variant at chr17:46152620:T:C (rs2532300, OR:1.16, 95% CIs:[1.10,1.22]) includes 1430 variants and spans 658 kb, indicating an association with the known inversion haplotype at 17q21.31[6].

We fine mapped the signal at 17q21.33 to five variants, with lead variant (chr17:49863260:C:A, rs3848456, OR:1.5, 95% CIs:[1.33-1.70] residing in a regulatory element (ENSR00001010694) 15.5 kb upstream of the TAC4 gene.

In the 19p13.3 region, which we reported in 2020,[7], we fine-mapped the signal to a single variant in *DPP9* (chr19:4717660:A:G). This variant is a missense variant in transcript ENST00000599248 but intronic in other transcripts including the MANE transcript (ENST00000262960.14).

In the 19p13.2 region, where we previously reported a variant associated with *TYK2*,[7] we find two independent signals, one of which is a damaging missense variant in *TYK2*, chr19:10352442:G:C (rs34536443, OR:1.50, 95% CIs:[1.36,1.65], CADD=25.1), and the second is an intronic variant of *ZGLP1* (19:10305768:G:A, rs73510898, OR:1.28, 95% CIs:[1.19,1.37]).

The signal at 19q13.33 was fine-mapped to ten variants, with the lead variant (chr19:48697960:C:T, rs368565, OR:1.15, 95%CIs [1.1-1.2]) in an intron of *FUT2* and variant chr19:48705753:T:C (rs503279) being in the 3' UTR of the MANE transcript for this gene.

In the 21q22.11 region, we described previously,[7] fine-mapping revealed three independent signals, for which the lead variants reside in the 5' UTR of *IFNAR2* (chr21:33230000:C:G,rs17860115, OR:1.24, 95% CIs:[1.19-1.30], CADD=10.1), an intronic region of *IL10RB* (chr21:33287378:C:T, rs8178521, OR:1.18, 95% CIs:[1.12,1.23]) and in a downstream long non-coding RNA (chr21:33959662:T:TAC, rs35370143, OR:1.26, 95% CIs:[1.17,1.36]).

Fine-mapping check for rare variants

To investigate whether the discovered signals in the primary GWAS analyses were underlain by variants that were rarer than the applied MAF threshold of >0.5%, we expanded our fine-mapping analysis around the lead signals to variants with allele frequency as low as 0.02%. We performed this additional analysis for the European population only, as this was the only population with a sufficiently large sample size to detect variants of such low frequency. For this analysis check, we performed all standard site QC procedures to variants with MAF>0.02%, calculated GWAS summaries with SAIGE within a window of 1.5 Mbp of either side of each EUR-discovered lead signal of main Table 1, re-calculated the matrix of all pairwise correlation coefficients and rerun fine-mapping with *susieR*, following the primary GWAS and fine-mapping procedure as described in Materials and Methods. For all of the 23 EUR-discovered signals, the lead variant in each credible set (i.e, variant with lowest P-value) remained the same and the size of each credible set changed only slightly in a few cases (Supplementary Table 9).

**Lead variant (MAF>0.5%)**	**Lead variant (MAF>0.02 %)**	**nCS MAF>0.5%**	**nCS MAF>0.02%**
chr1:155066988:C:T	chr1:155066988:C:T	9	9
chr1:155197995:A:G	chr1:155197995:A:G	3	3
chr3:45796521:G:T	chr3:45796521:G:T	1	1
chr3:45859597:C:T	chr3:45859597:C:T	9	9
chr3:146517122:G:A	chr3:146517122:G:A	9	9
chr5:131995059:C:T	chr5:131995059:C:T	32	33
chr6:32623820:T:C	chr6:32623820:T:C	33	32
chr9:21206606:C:G	chr9:21206606:C:G	3	3
chr11:34482745:G:A	chr11:34482745:G:A	4	4
chr12:132489230:GC:G	chr12:132489230:GC:G	25	25
chr13:112889041:C:T	chr13:112889041:C:T	4	4
chr15:93046840:T:A	chr15:93046840:T:A	2	2
chr16:89196249:G:A	chr16:89196249:G:A	4	5
chr17:46152620:T:C	chr17:46152620:T:C	1430	1426
chr17:49863260:C:A	chr17:49863260:C:A	5	4
chr19:4717660:A:G	chr19:4717660:A:G	1	1
chr19:10305768:G:A	chr19:10305768:G:A	3	3
chr19:10352442:G:C	chr19:10352442:G:C	1	1
chr19:48697960:C:T	chr19:48697960:C:T	10	10
chr21:33230000:C:A	chr21:33230000:C:A	16	17
chr21:33287378:C:T	chr21:33287378:C:T	33	33
chr21:33959662:T:TAC	chr21:33959662:T:TAC	23	22

Supplementary Table 9: Fine-mapping results for the EUR-discovered signals for the primary results using variants with MAF $>$ 0.5% and the expanded analysis using variants with MAF $>$ 0.02%. The lead variant (i.e, having the lowest P-value) and the number of variants (nCS) included in each credible set are shown for each analysis. Provided variant ids correspond to chr:pos$_{hg38}$:ref$_{hg38}$:alt.

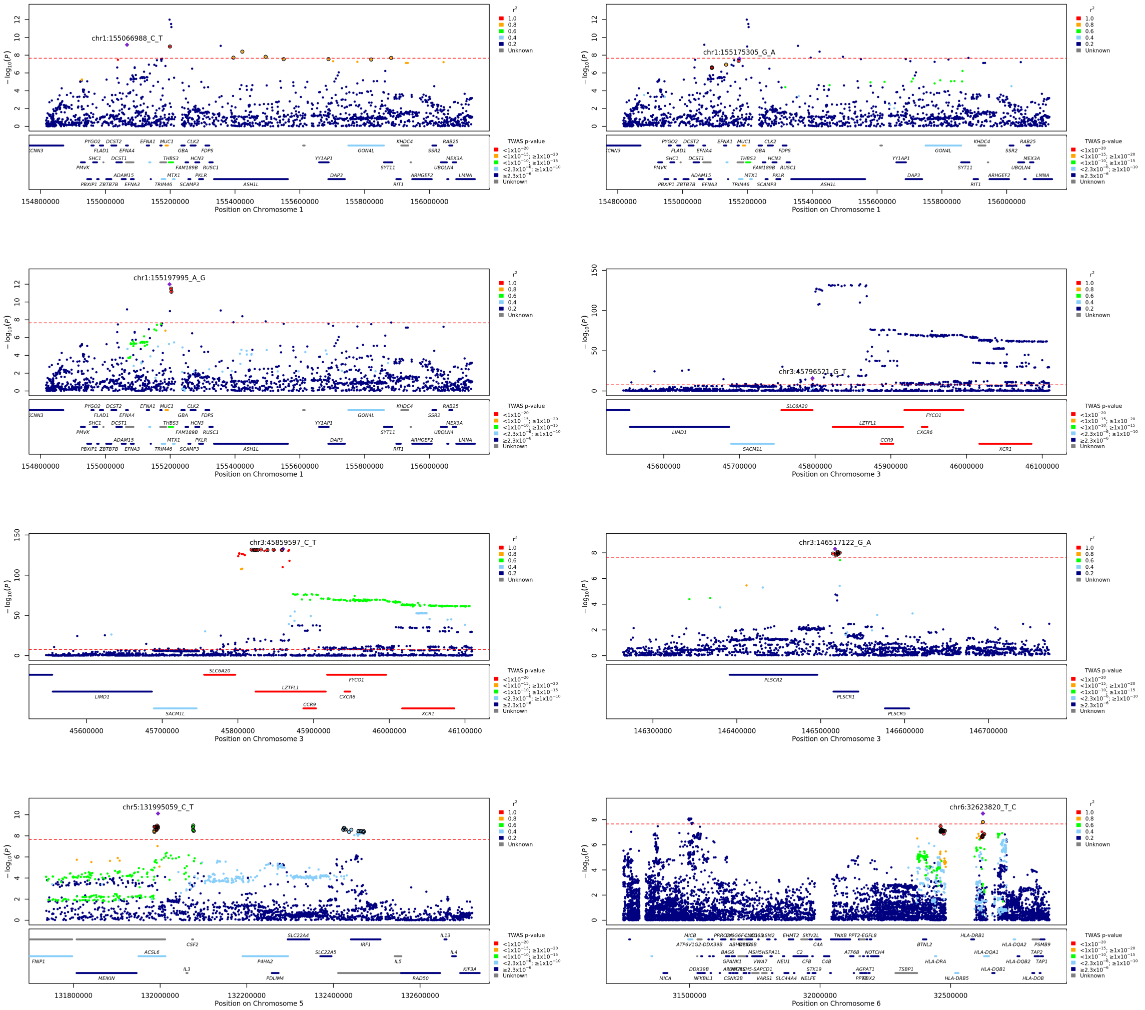
chr1:155066988_C_T
chr1:155175305_G_A
chr1:155197995_A_G
chr3:45796521_G_T
chr3:45859597_C_T
chr3:146517122_G_A
chr5:131995059_C_T
chr6:32623820_T_C
Position on Chromosome 1
Position on Chromosome 3
Position on Chromosome 5
Position on Chromosome 6
TWAS p-value

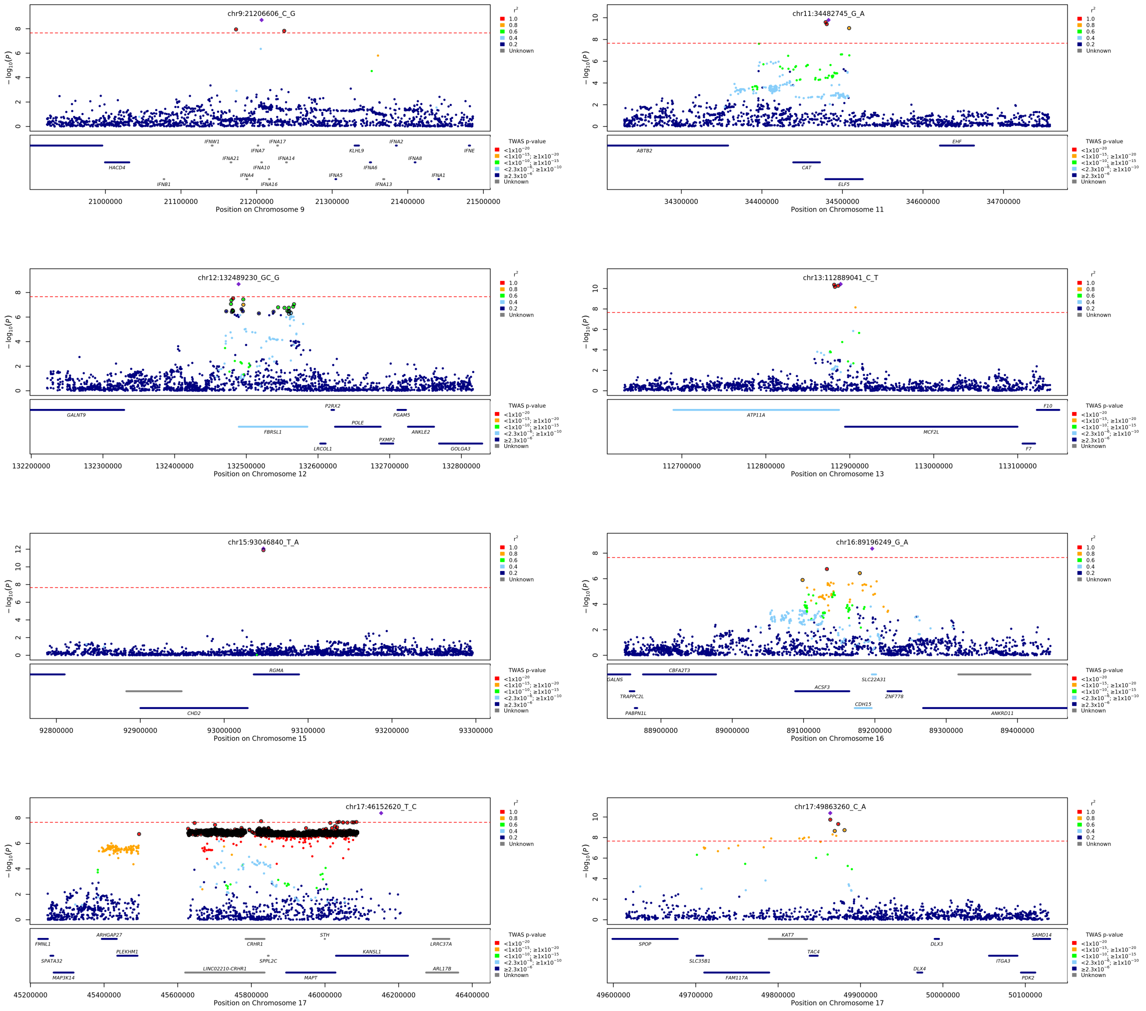

chr9:21206606_C_G
Position on Chromosome 9
chr11:34482745_G_A
Position on Chromosome 11
chr12:132489230_GC_G
Position on Chromosome 12
chr13:112889041_C_T
Position on Chromosome 13
chr15:93046840_T_A
Position on Chromosome 15
chr16:89196249_G_A
Position on Chromosome 16
chr17:46152620_T_C
Position on Chromosome 17
chr17:49863260_C_A
Position on Chromosome 17
TWAS p-value
Unknown

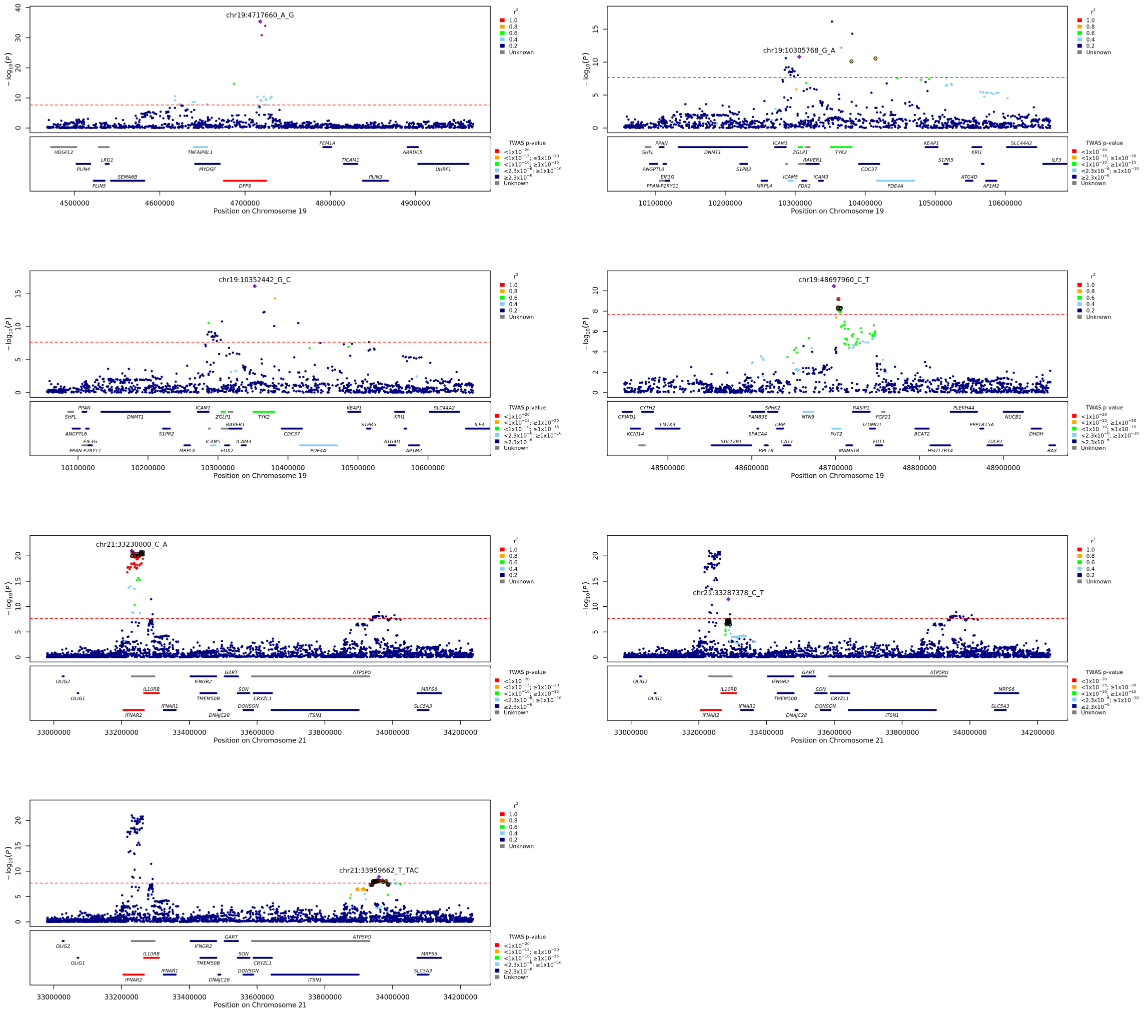

Supplementary Figure 15: Locuszoom figures for the signals found in the per-population analyses. Upper panels show lead signals and LD calculated in EUR (n=5,989) with all other loci in the window shown. r^2 values in the legend denote upper limits, *i.e.* 0.2=[0,0.2], 0.4=(0.2,0.4], 0.6=(0.4,0.6], 0.8=(0.6,0.8],1=(0.8,1]. Credible sets for each displayed signal that were inferred with susieR are displayed with outline black circles. The red dashed line shows the Bonferroni-corrected P-value=2.2×10^{-8} for Europeans. On the bottom panels an hg38 gene track is displayed with colors matching significance from the metaTWAS analysis in discrete bins shown.

META, LD AFR, chr2:60480453:A:G

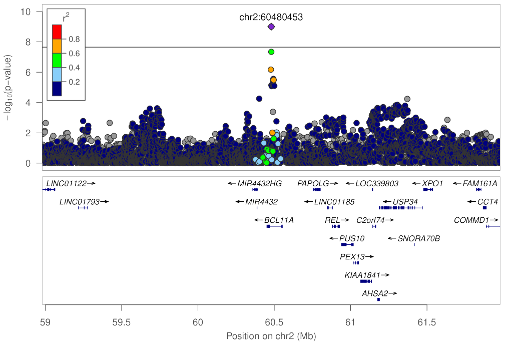

META, LD AFR, chr6:41515007:A:C

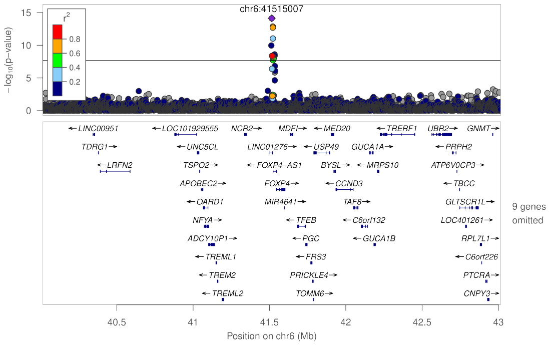

META, LD EAS, chr1:155175305:G:A

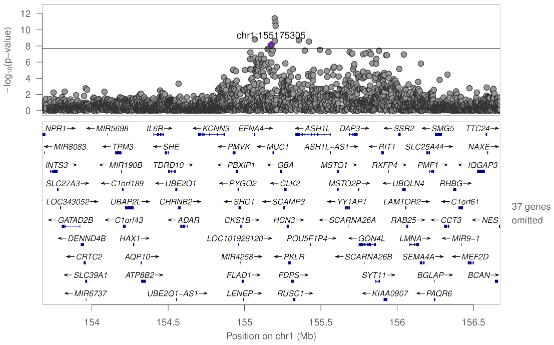

META, LD EAS, chr2:60480453:A:G

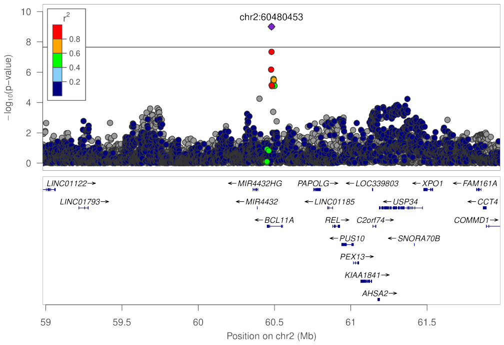

META, LD EAS, chr6:41515007:A:C

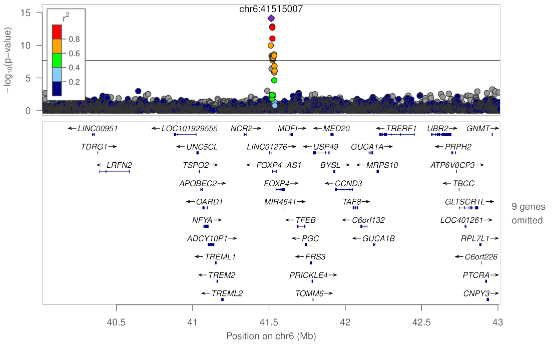

META, LD EUR, chr1:155175305:G:A

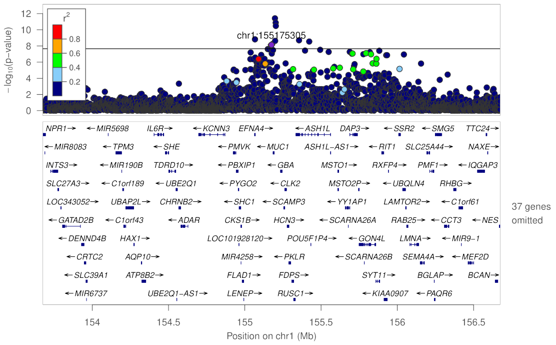

META, LD EUR, chr2:60480453:A:G

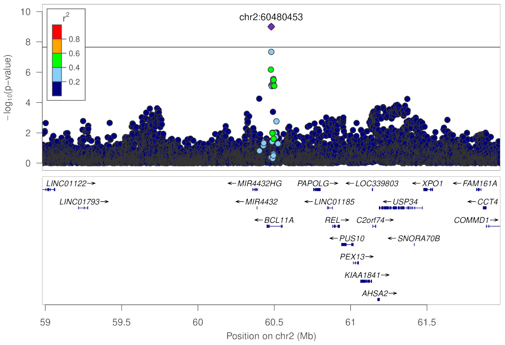

META, LD EUR, chr6:41515007:A:C

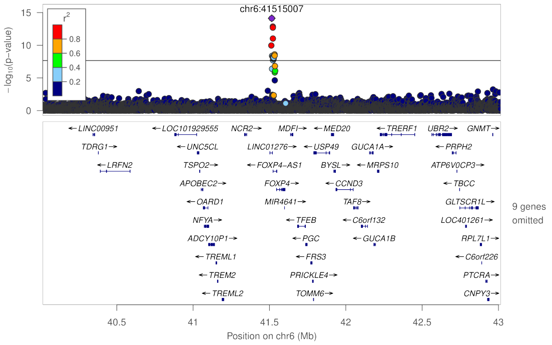

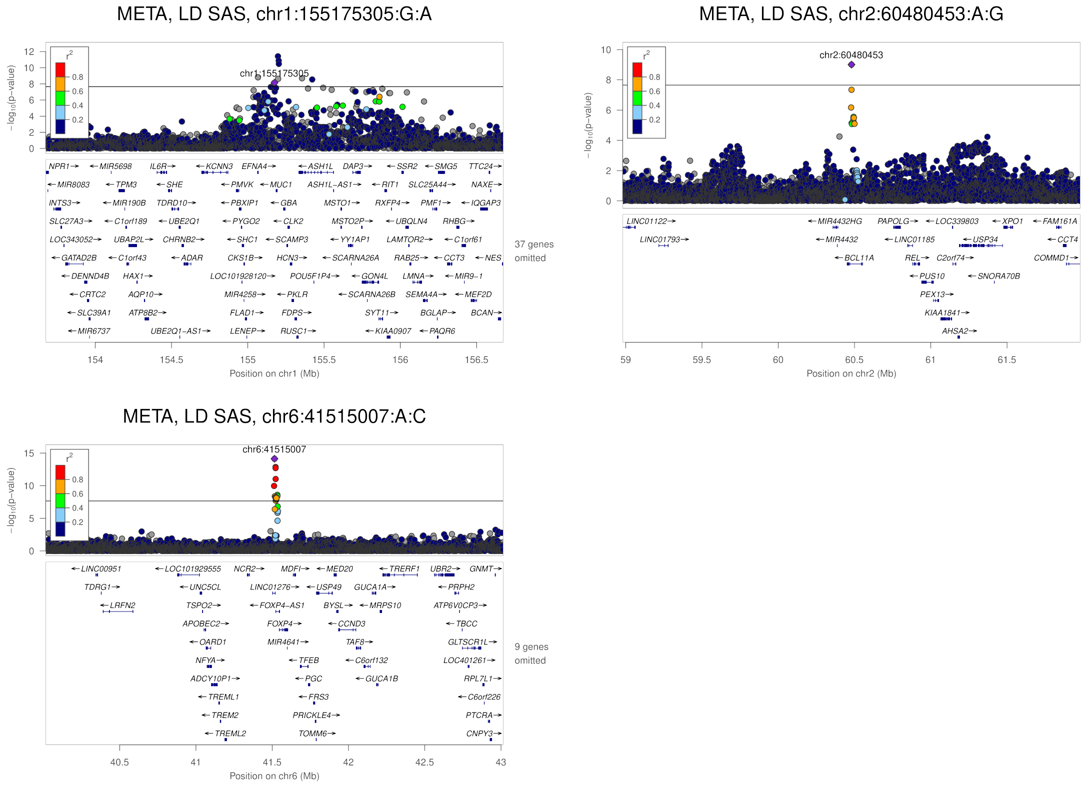

Supplementary Figure 16: Locuszoom figures for the multi-ancestry meta-analysis signals with different panels for LD calculated in the four populations of this study (AFR, EAS, EUR, SAS). r^2 values in the legend denote upper limits, *i.e.* 0.2=[0,0.2], 0.4=(0.2,0.4], 0.6=(0.4,0.6], 0.8=(0.6,0.8],1=(0.8,1]. The red dashed line shows the Bonferroni-corrected P-value=2.2×10^{-8} (tested variants in meta-analysis was equal to the EUR tested variants).

Sex- and age- stratified analysis

We performed sex- (< 60 vs. ≥ 60) and age-stratified analyses. We did not obtain significant evidence for sex- specific effects (Supplementary Figure 17). The locus at chr3:45801750:G:A (rs13071258) in the European population had a significantly stronger effect in the younger age group ($OR = 3.34, 95\%CI = 2.98 - 3.75$ vs. $OR = 2.1, 95\%CI = 1.88 - 2.34$).

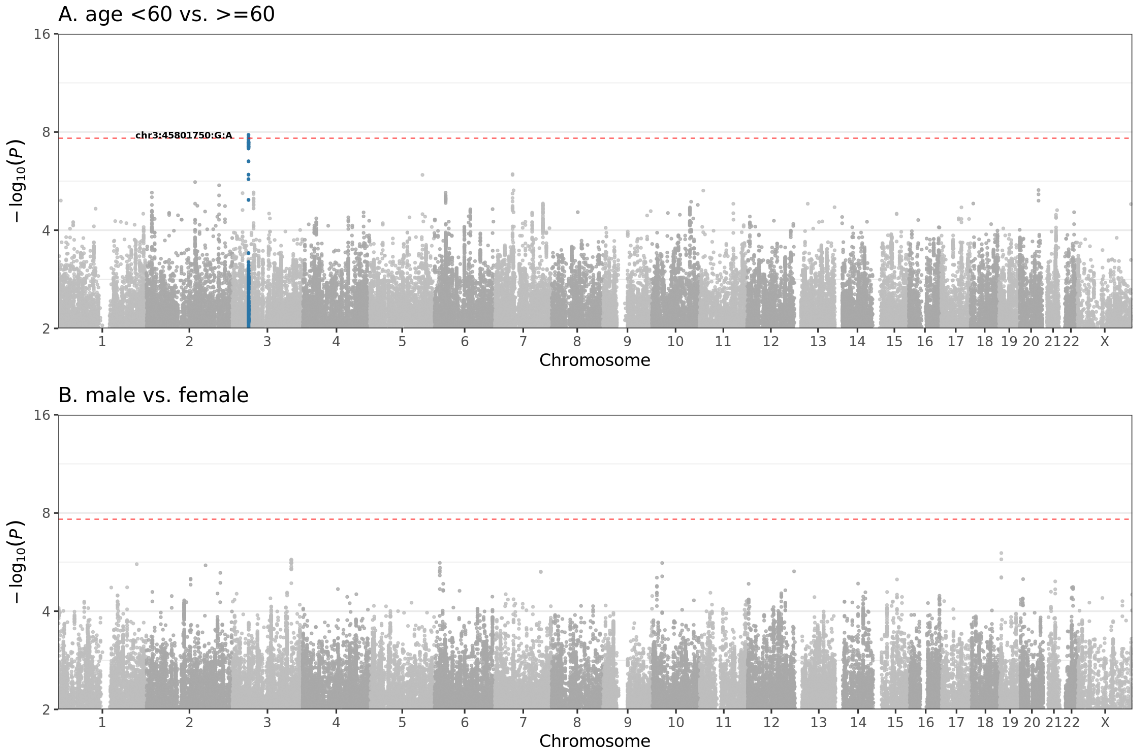

Supplementary Figure 17: Manhattan plot for t-test P-values obtained from comparison of stratified GWAS analyses by age and sex. GWAS analyses were run for stratified subsets of the severe vs. mild+100K analysis for individuals with (A) age >60 vs. ≥ 60 and (B) males vs. females. For each analysis we then performed a two-sided t-test comparing between-group effect sizes per variant. Red dashed line corresponds to Bonferroni-corrected P-value $= 2 \times 10^{-8}$.

Matched case-control analysis

In order to assess whether the observed imbalance in age, sex and BMI (Supplementary Figures 9, 10) had an effect on our results, we also performed a matched case-control analysis. We first selected a subset of cases (n_{EUR}=3864, n_{SAS}=555, n_{AFR}=265, n_{EAS}=168) and controls (n_{EUR}=31,371, n_{SAS}=3,044, n_{AFR}=1,043, n_{EAS}=274) for which we had BMI information. We then performed propensity score matching with Rfunction *matchit* to match a subset of controls to cases based on age, sex and BMI and run GWAS analyses with *SAIGE*, including BMI as a covariate in addition to the other primary covariates (i.e, *sex*, *age*, $age \times sex$, age^2 and 20 PCs) and separately for each ancestry group (Supplementary Figure 18).

As 22 out 25 association signals were discovered in the European population, we first assessed how the effect size and P-values changed in the matched GWAS analysis versus the original unmatched study for EUR. We observed that they were strongly correlated both genome-wide and for our lead variants (Supplementary Figure 19, Supplementary Figure 20, left panels). For our lead variants, we also performed a GWAS analysis for EUR that used a random sub-sample of unmatched controls of the same size as the size of the controls of the matched study to assess the effect of the matching procedure versus the loss of power due to reduction in sample size of the matched study. The reduction in significance in the matched study is of similar magnitude as that of the unmatched study with the same sample size (Supplementary Figure 20, left versus middle panels). Adding BMI as covariate in the unmatched study produced similar estimates for effect sizes and P-values (Supplementary Figure 20, right panels).

We also assessed how results for the three associations that were discovered with the multi-ancestry meta-analysis were affected by case/control mismatches for age, sex and bmi by comparing the effect sizes across ancestries and meta-analysed results for the original versus the matched study (left vs. right panels, Supplementary Figure 20).

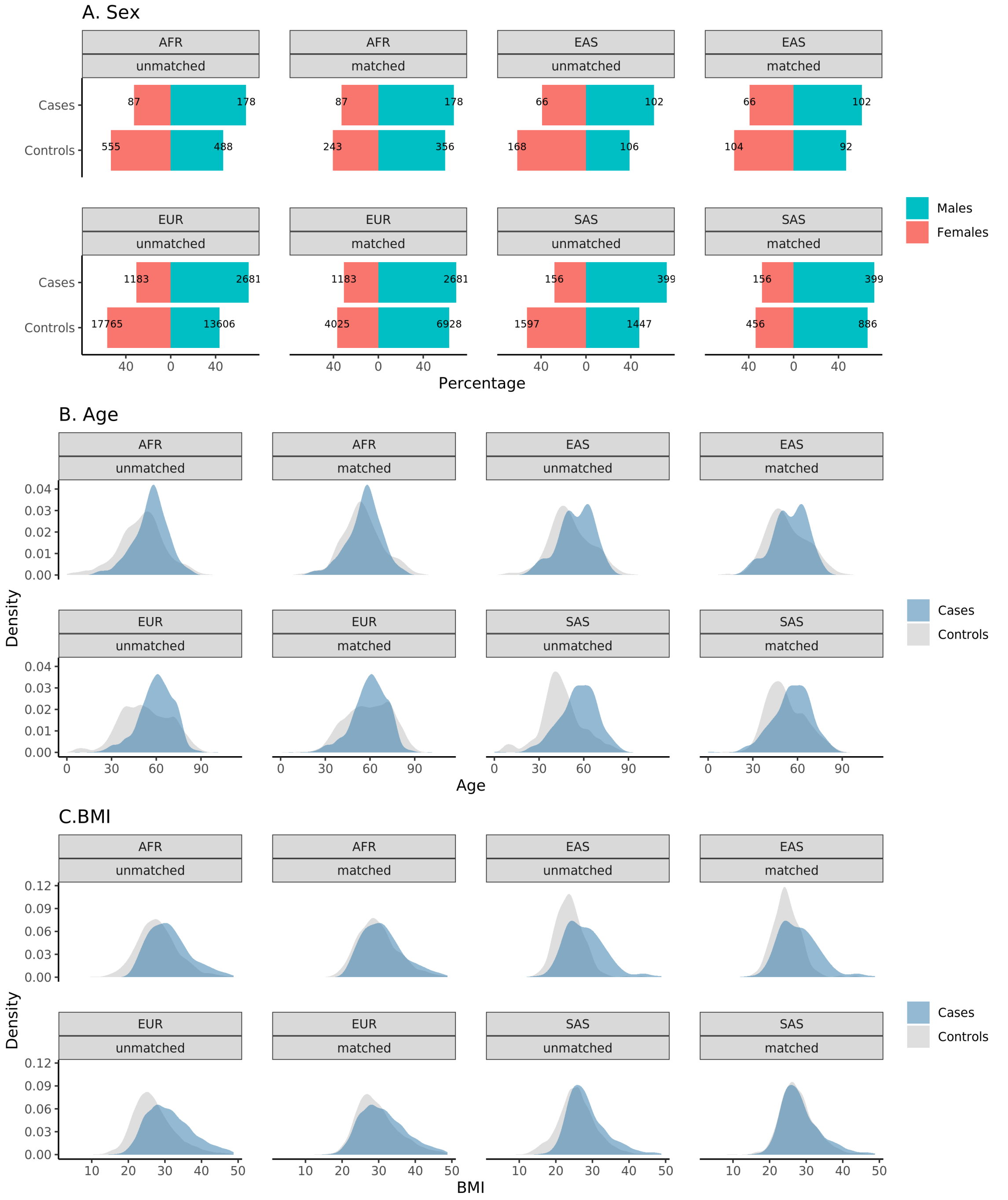

Supplementary Figure 18: Sex, age and BMI distributions for cases and unmatched and matched controls. The "unmatched" panels for each ancestry include all individuals that were used in the main analyses of this study and for which we had BMI measurements. The matched control cohorts (a subset of the unmatched controls) were generated with propensity score matching.

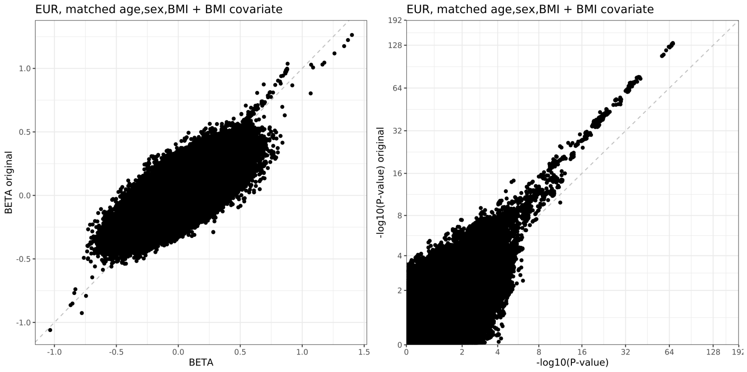

Supplementary Figure 19: Genome-wide Results for effect size (BETA) and P-values for a EUR SAIGE GWAS analysis using the age,sex,bmi- matched case/control data. For this analysis default covariates of age, sex, age × sex, 20 PCs and BMI were used.

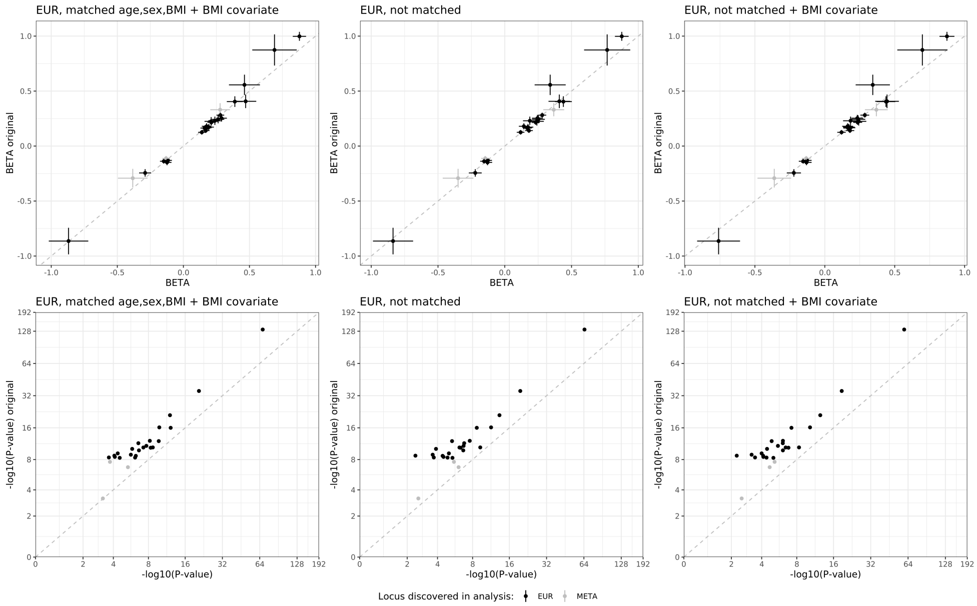

Supplementary Figure 20: Results for lead variants of this study comparing effect size (BETA) and P-values for EUR GWAS analyses using cases with matched and unmatched controls. Left panel shows the results of the matched study which used default covariates of age, sex, age × sex and 20 PCs. Middle panels show results of a study using unmatched controls of the same sample size as the matched study and using covariates as the principal study gwas using only default covariates. Right panels show results of an unmatched study using default + BMI as covariate. Results for EUR-discovered loci are shown in black and with grey the multi-ancestry meta-analysis results are shown. Error bars for BETA represent standard errors of estimates.

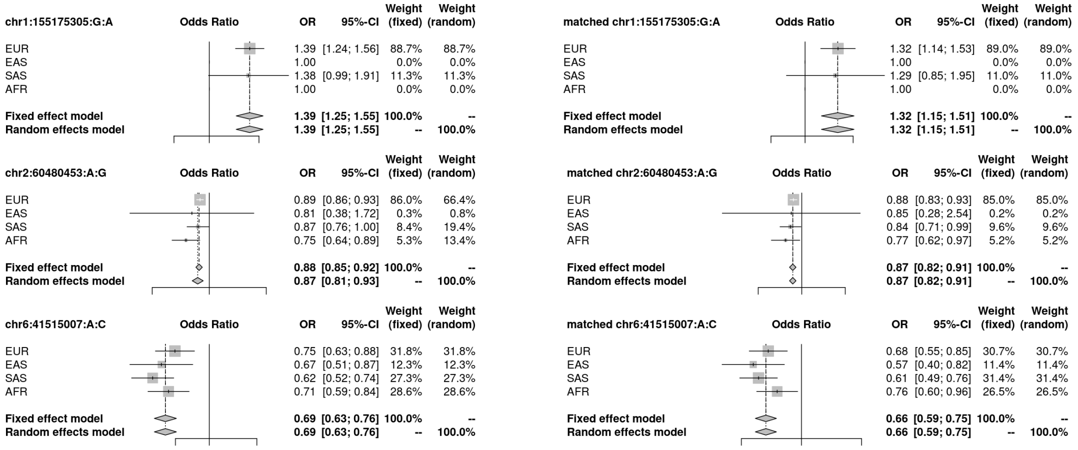

Supplementary Figure 21: Comparison of effect sizes between unmatched (left panels) and matched (right panels) control results for the three loci that were found significant in the multi-ancestry meta-analysis. Whiskers show 95% CI. For the matched study, controls were matched to cases by propensity score matching to cases for which we had BMI information (using age, sex and bmi as matching covariates and performed separately for each ancestry): cases n_{EUR}=3864, n_{SAS}=555, n_{AFR}=265, n_{EAS}=168; controls n_{EUR}=31,371, n_{SAS}=3,044, n_{AFR}=1,043, n_{EAS}=274.

GWAS analysis using the mild Covid-19 cohort only as controls

Lead variant	AF_{case}	$AF_{100K+mild}$	AF_{100K}	AF_{mild}	OR	OR_{CI}	Pval	OR_{mild}	OR_{mild} CI	$Pval_{mild}$	Gene
chr1:155066988:C:T	0.0101	0.00521	0.00526	0.00464	2.4	1.82-3.16	6.8E-10	1.96	1.19-3.24	0.00813	*EFNA4*
chr1:155175305:G:A	0.0408	0.0309	0.0308	0.0265	1.39	1.24-1.55	7.16E-09	1.33	1.04-1.72	0.0255	*TRIM46*
chr1:155197995:A:G	0.0874	0.112	0.112	0.12	0.783	0.732-0.838	1.02E-12	0.643	0.547-0.757	1.01E-07	*THBS3*
chr2:60480453:A:G	0.363	0.39	0.389	0.391	0.884	0.849-0.919	9.85E-10	0.901	0.817-0.994	0.0365	*BCL11A*
chr3:45796521:G:T	0.162	0.133	0.132	0.16	1.29	1.21-1.37	9.9E-17	1.02	0.894-1.15	0.81	*SLC6A20*
chr3:45859597:C:T	0.143	0.0679	0.0682	0.0687	2.71	2.51-2.94	1.97E-133	2.11	1.81-2.45	4.71E-22	*LZTFL1*
chr3:146517122:G:A	0.0938	0.0787	0.0785	0.0774	1.25	1.16-1.35	4.94E-09	1.28	1.09-1.5	0.00292	*PLSCR1*
chr5:131995059:C:T	0.193	0.167	0.167	0.158	1.2	1.13-1.26	7.65E-11	1.3	1.15-1.47	2.01E-05	*ACSL6*
chr6:32623820:T:C	0.323	0.353	0.353	0.347	0.878	0.841-0.917	3.26E-09	0.887	0.803-0.981	0.0193	*HLA-DQA1*
chr6:41515007:A:C	0.981	0.986	0.986	0.984	0.687	0.625-0.756	7.59E-15	0.915	0.642-1.3	0.624	*LINC01276*
chr9:21206606:C:G	0.0195	0.0124	0.0125	0.0113	1.74	1.45-2.09	1.93E-09	1.4	0.956-2.04	0.0838	*IFNA10*
chr11:34482745:G:A	0.348	0.381	0.38	0.393	0.871	0.835-0.909	1.61E-10	0.831	0.753-0.918	0.000241	*ELF5*
chr12:132489230:GC:G	0.522	0.495	0.496	0.483	1.13	1.09-1.18	2.08E-09	1.16	1.05-1.27	0.00257	*FBRSL1*
chr13:112889041:C:T	0.249	0.221	0.22	0.223	1.18	1.12-1.24	3.71E-11	1.2	1.07-1.33	0.0015	*ATP11A*
chr15:93046840:T:A	0.986	0.993	0.993	0.988	0.422	0.333-0.534	8.61E-13	0.796	0.531-1.19	0.269	*RGMA*
chr16:89196249:G:A	0.167	0.145	0.145	0.155	1.19	1.12-1.26	4.4E-09	1.08	0.954-1.23	0.22	*SLC22A31*
chr17:46152620:T:C	0.202	0.232	0.232	0.231	0.862	0.82-0.906	4.19E-09	0.879	0.782-0.989	0.0314	*KANSL1*
chr17:49863260:C:A	0.0404	0.0279	0.0281	0.0269	1.5	1.33-1.7	4.19E-11	1.32	1.02-1.7	0.034	.
chr19:4717660:A:G	0.365	0.305	0.304	0.305	1.32	1.27-1.38	3.91E-36	1.37	1.24-1.51	2.17E-10	*DPP9*
chr19:10305768:G:A	0.11	0.0905	0.0906	0.0947	1.28	1.19-1.37	1.57E-11	1.25	1.07-1.45	0.0039	*ZGLP1*
chr19:10352442:G:C	0.0664	0.0479	0.0481	0.0405	1.5	1.36-1.65	6.98E-17	1.64	1.35-2	5.94E-07	*TYK2*
chr19:48697960:C:T	0.469	0.438	0.438	0.439	1.15	1.1-1.2	3.55E-11	1.08	0.977-1.19	0.134	*FUT2*
chr21:33230000:C:A	0.357	0.31	0.309	0.314	1.24	1.19-1.3	9.69E-22	1.17	1.06-1.29	0.00212	*IFNAR2*
chr21:33287378:C:T	0.294	0.265	0.265	0.261	1.18	1.12-1.23	3.53E-12	1.21	1.09-1.34	0.000334	*IL10RB*
chr21:33959662:T:TAC	0.0982	0.0808	0.0809	0.083	1.26	1.17-1.36	1.24E-09	1.21	1.03-1.42	0.0216	*LINC00649*

Supplementary Table 10: Allele frequency comparison and GWAS results for lead variants of the study using unrelated individuals with EUR predicted ancestry with COVID-19 severe individuals as cases and COVID-19 positive individuals with only mild symptoms as controls. Allele frequencies shown are calculated for individuals with EUR predicted ancestry that are part of severe COVID-19 cases (AF_{case}, n=5,989), EUR controls used in the main study GWAS comprised of 100K and mild ($AF_{100K+mild}$, n=42,891), EUR controls comprised of 100K (AF_{100K}, n=41,384) and EUR controls comprised of COVID-19 mild individuals (AF_{mild}, n=1,507) and were calculated using plink2 with the reference allele being hg_{38}. Odds ratio with 95% confidence interval and P-value from main study results from table 1 (OR, OR_{CI}, $Pval$) are compared with results from a GWAS analysis that used only COVID-19 individuals with mild symptoms as controls (OR_{mild}, OR_{mild} CI, $Pval_{mild}$). Provided variant ids correspond to chr:pos$_{hg38}$:ref$_{hg38}$:alt.

Heritability

We estimated the SNP-based heritability values of Covid-19 severity and four Covid-19 phenotypes of HGI v6 by applying both the high-definition likelihood (HDL) [8] and LD score regression (LDSC) [9] methods on the GWAS summary statistics. The HDL method is expected to produce more consistent estimate than LDSC. [8]

Except for the GenOMICC severity phenotype of Covid-19, which had an estimated heritability of 5.7% (s.e. 1.7%) by HDL and 11.3% (s.e. 2.5%) by LDSC, the other Covid-19 phenotypes all had heritability estimates close to zero (Supplementary Fig. 22, Supplementary Table 11).

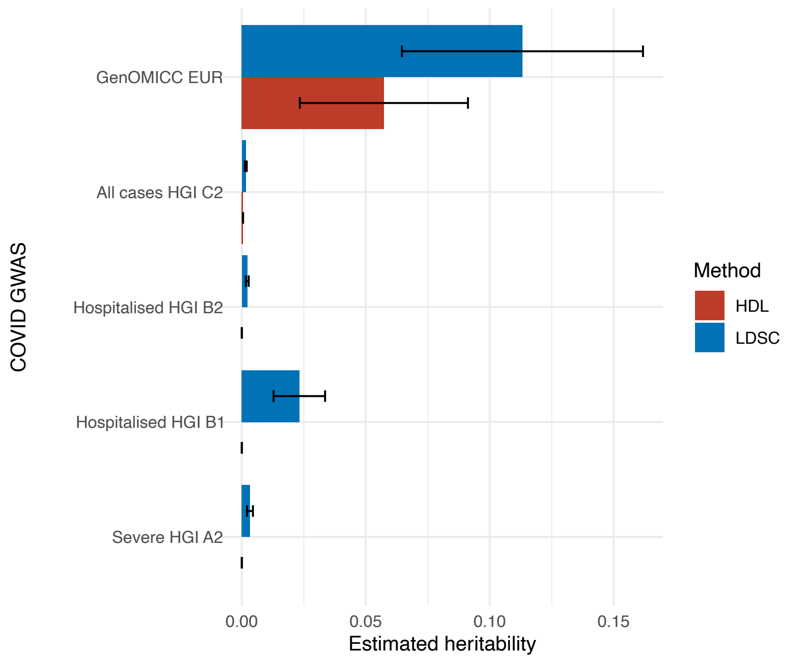

Supplementary Figure 22: Heritability estimates of Covid-19 based on GWAS summary statistics. Whiskers represent the estimated 95% confidence intervals. See Supplementary Table 11 for details.

GWAS	n	$Heritability_{HDL}$	SE_{HDL}	$Pvalue_{HDL}$	$Heritability_{LDSC}$	SE_{LDSC}	$Intercept_{LDSC}$	$Intercept_{SE}$
GenOMICC EUR	5,989	0.057	0.017	0.00092	0.11	0.025	0.98	0.0095
All cases HGI C2	112,612	0.0005	2e-06	0	0.0016	0.0002	1	0.0072
Hospitalised HGI B2	24,274	0	0		0.0022	0.0003	1	0.007
Hospitalised HGI B1	14,480	0	0		0.023	0.0053	1	0.0065
Severe HGI A2	8,779	0	0		0.0033	0.0006	1	0.008

Supplementary Table 11: Heritability estimates of Covid-19 based on GWAS summary statistics. The SNP-based narrow-sense heritabilities of Covid-19 severity and four alternatively-defined Covid-19 phenotypes were estimated using both the high-definition likelihood (HDL) and LD score regression (LDSC) methods. Comparisons are made with HGIv6[5] ALL leave 23andme 20210607 analyses; A-C2 used population controls, B1 used test-negative controls.

TWAS

eQTL TWAS

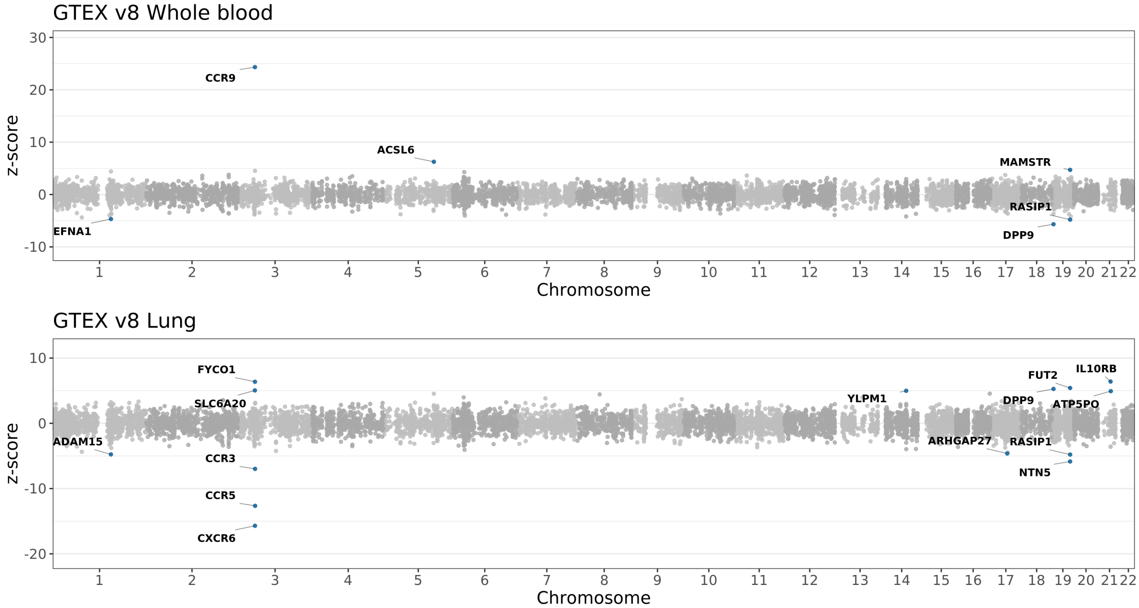

Supplementary Figure 23: TWAS results from analysis of eQTL models from whole blood and lung tissues in GTEXv8. Z-scores showing the direction of effect for the genotype-inferred expression of transcripts that encode protein-coding genes in whole blood and lung tissue (GTEx v.8) are shown (with tested genes n=10,473 and n=12,484, respectively). All significant genes at Bonferroni-corrected threshold P-value 4.77×10^{-6} and 4×10^{-6} for whole blood and Lung, respectively, are highlighted with blue and annotated.

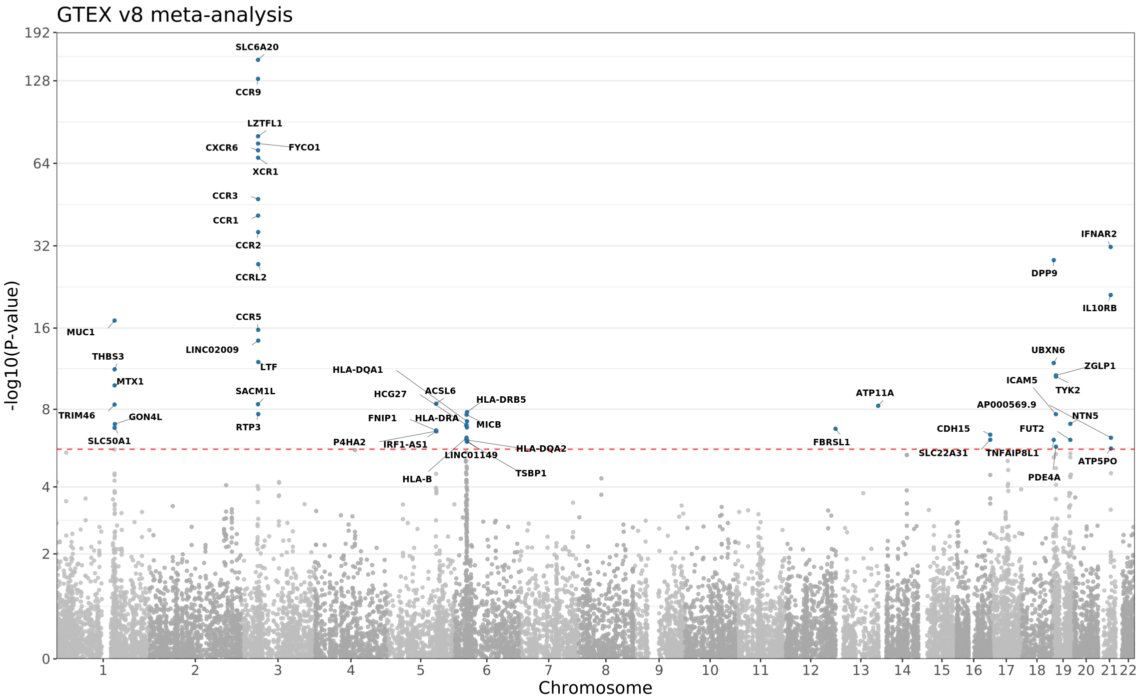

Supplementary Figure 24: TWAS results from meta-analysis of eQTL models from all tissues in GTEXv8. The number of tested genes was 21,813 and significant genes at Bonferroni-corrected threshold $P < 2.3 \times 10^{-6}$ (red dashed line) are highlighted with blue and annotated.

Colocalisation sensitivity analysis

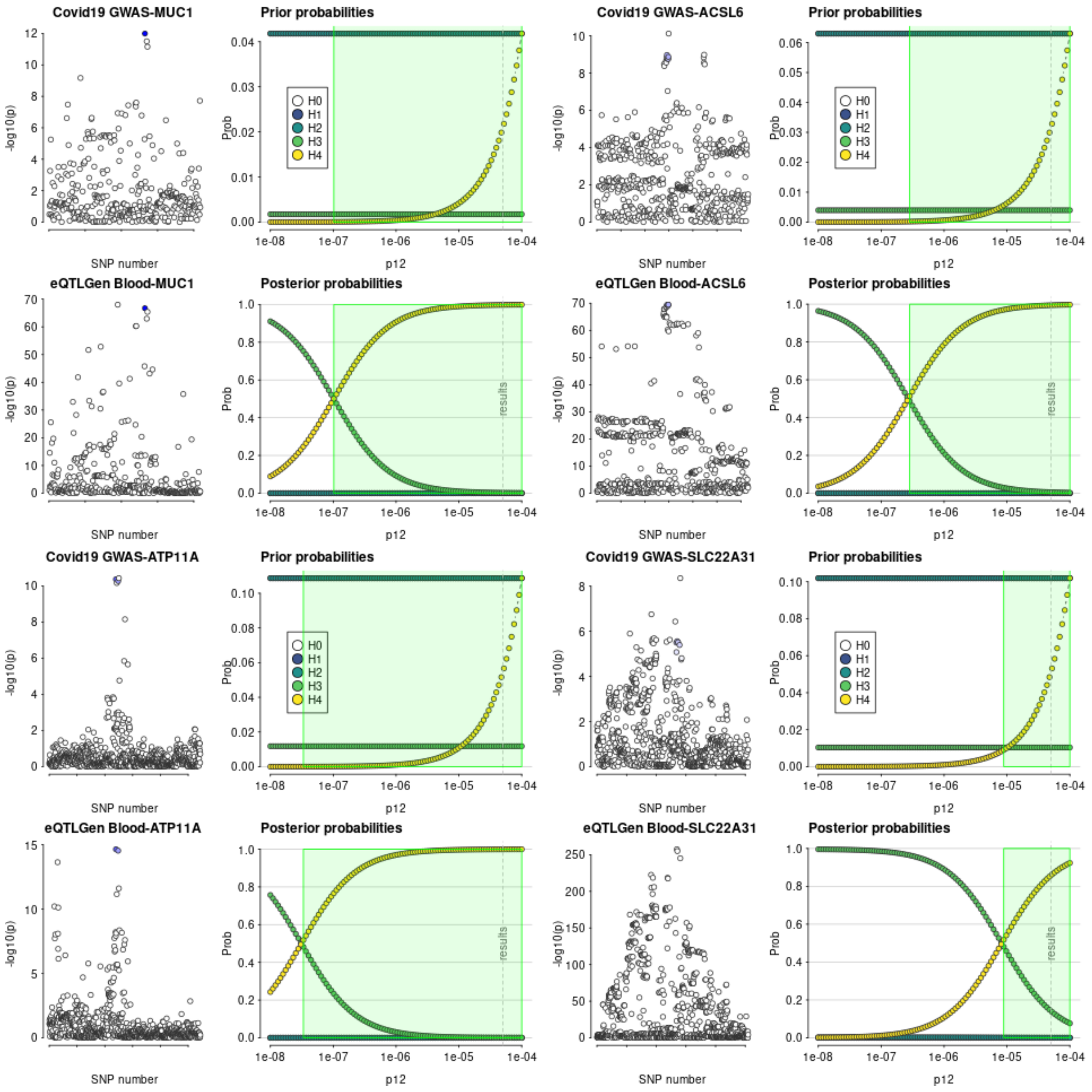

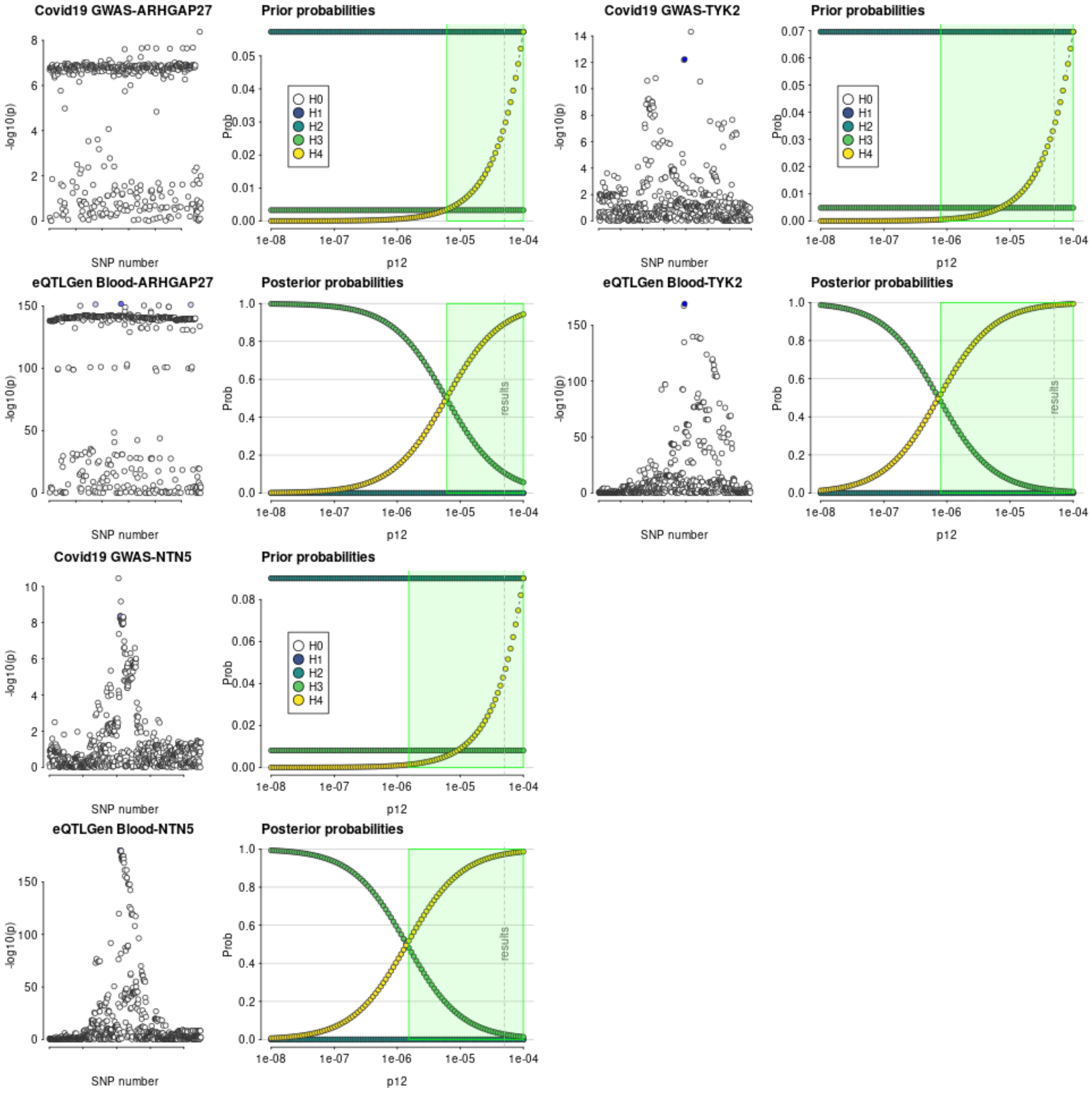

Supplementary Figure 25: Sensitivity analysis for colocalisation of TWAS-significant loci in blood (eQTLgen data) with GWAS signals. Evidence for the following hypotheses is plotted across a range of prior probabilities: H0 - neither trait has a genetic association in the region; H1 - only trait 1 has a genetic association in the region; H2 - only trait 2 has a genetic association in the region; H3 - both traits are associated, but with different causal variants; H4 - both traits are associated and share a single causal variant.

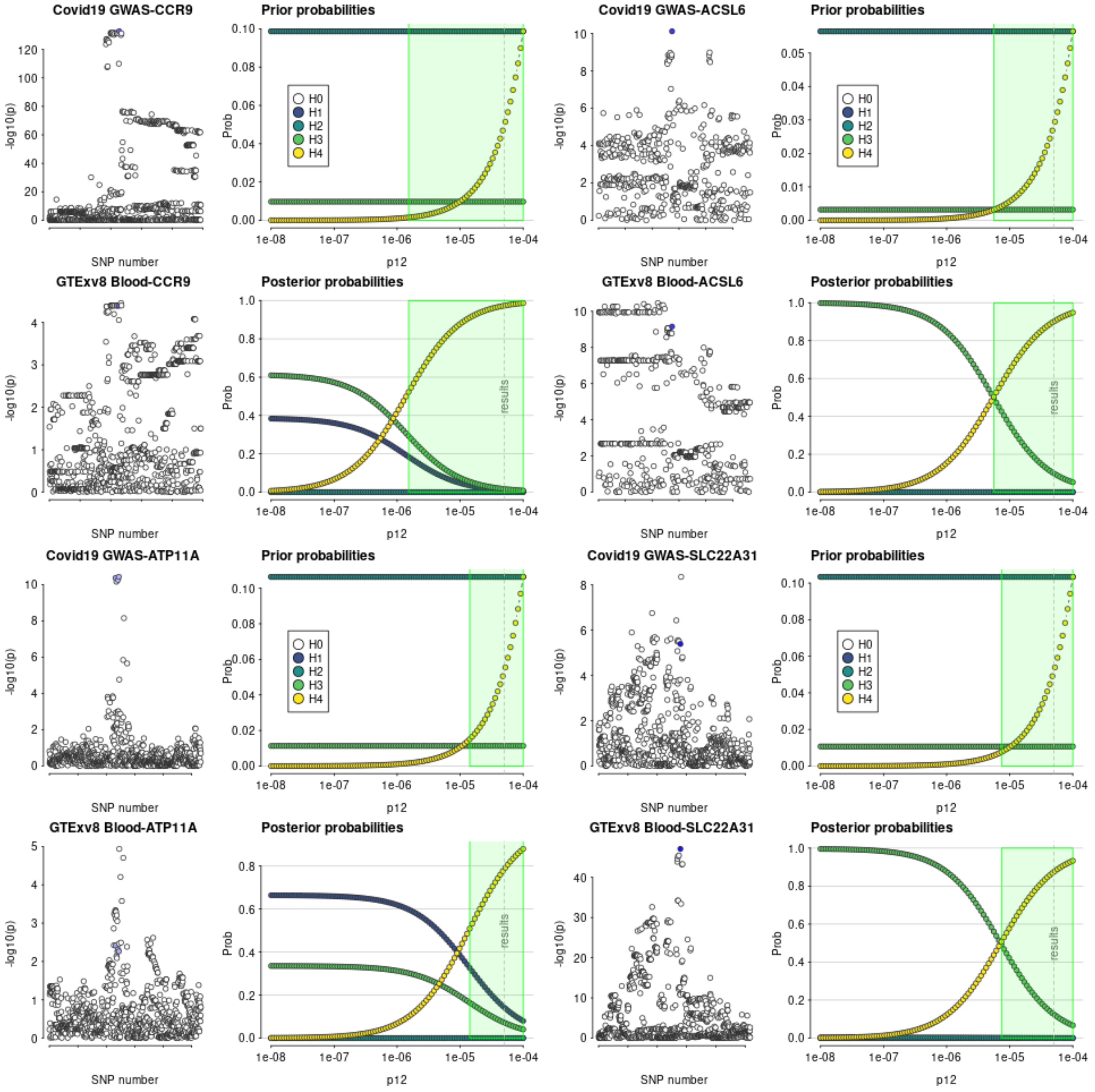

Covid19 GWAS-CCR9
Prior probabilities
Covid19 GWAS-ACSL6
Prior probabilities
GTExv8 Blood-CCR9
Posterior probabilities
GTExv8 Blood-ACSL6
Posterior probabilities
Covid19 GWAS-ATP11A
Prior probabilities
Covid19 GWAS-SLC22A31
Prior probabilities
GTExv8 Blood-ATP11A
Posterior probabilities
GTExv8 Blood-SLC22A31
Posterior probabilities
H0
H1
H2
H3
H4
-log10(p)
SNP number
Prob
p12
results

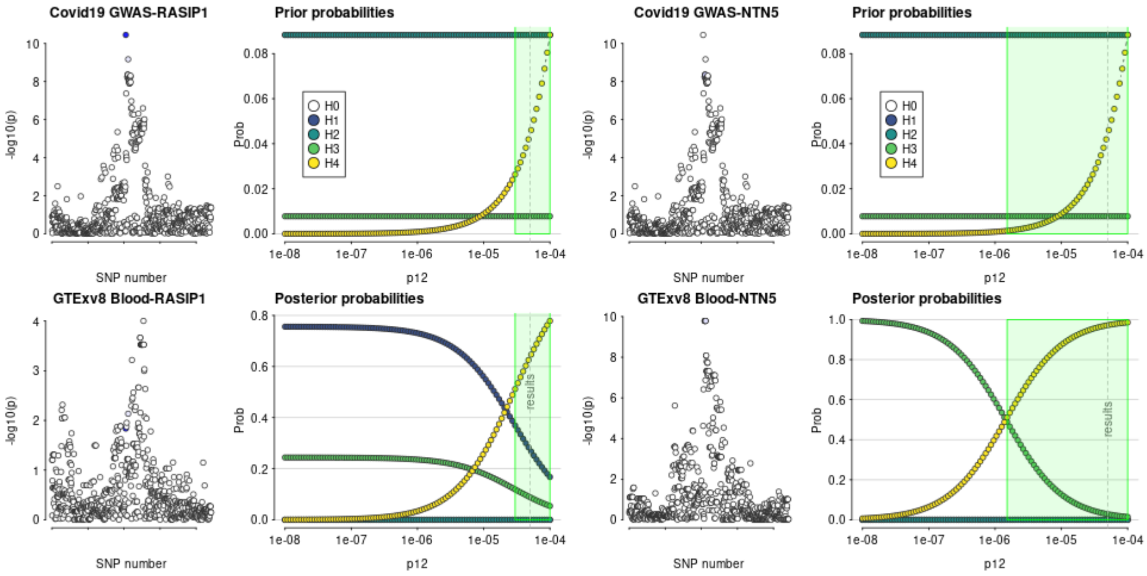

Supplementary Figure 26: Sensitivity analysis for colocalisation of TWAS-significant loci in blood (GTEXv8 data) with GWAS signals. Evidence for the following hypotheses is plotted across a range of prior probabilities: H0 - neither trait has a genetic association in the region; H1 - only trait 1 has a genetic association in the region; H2 - only trait 2 has a genetic association in the region; H3 - both traits are associated, but with different causal variants; H4 - both traits are associated and share a single causal variant.

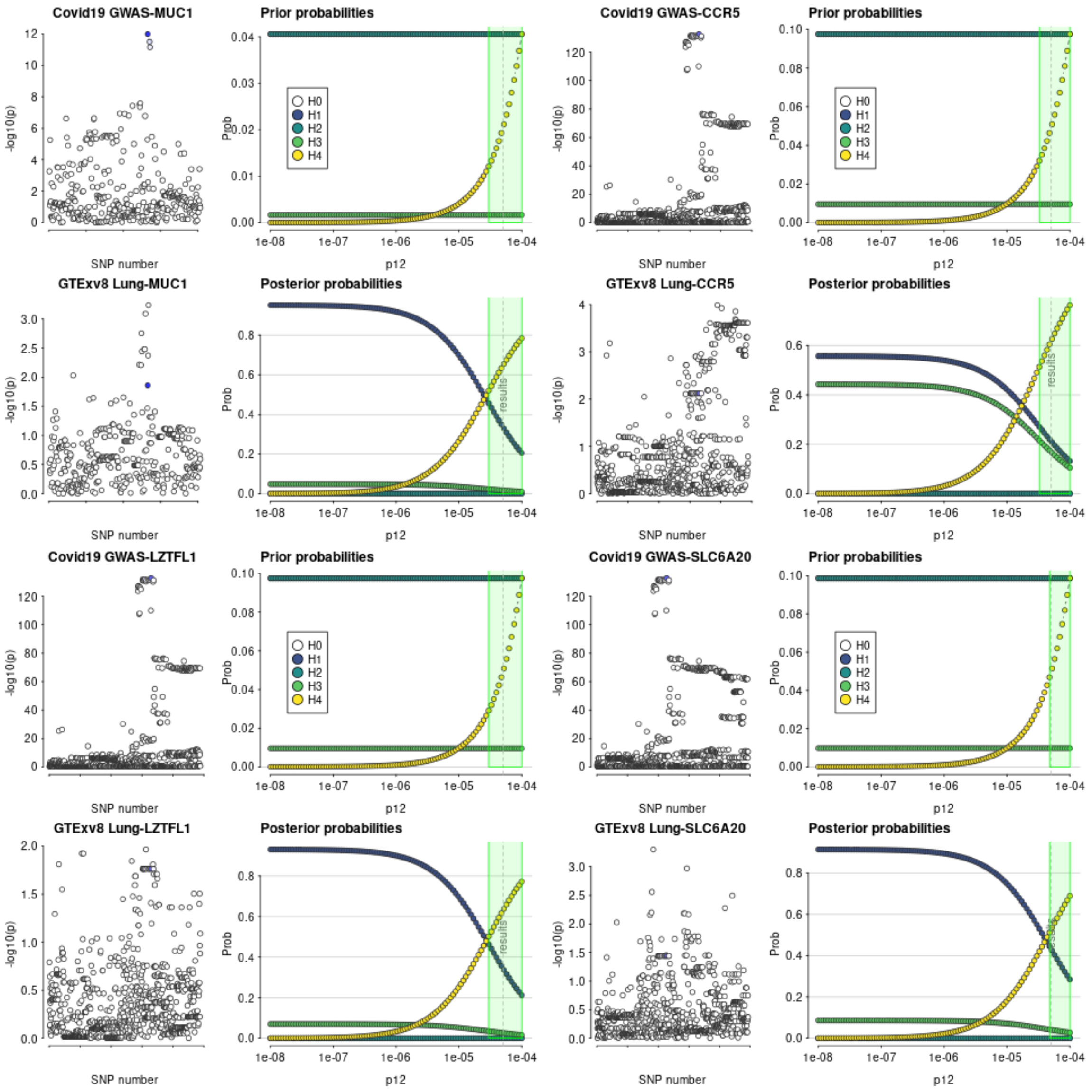
Covid19 GWAS-MUC1
Prior probabilities
Covid19 GWAS-CCR5
Prior probabilities
GTExv8 Lung-MUC1
Posterior probabilities
GTExv8 Lung-CCR5
Posterior probabilities
Covid19 GWAS-LZTFL1
Prior probabilities
Covid19 GWAS-SLC6A20
Prior probabilities
GTExv8 Lung-LZTFL1
Posterior probabilities
GTExv8 Lung-SLC6A20
Posterior probabilities
-log10(p)
SNP number
Prob
p12
H0
H1
H2
H3
H4
results
1e-08
1e-07
1e-06
1e-05
1e-04

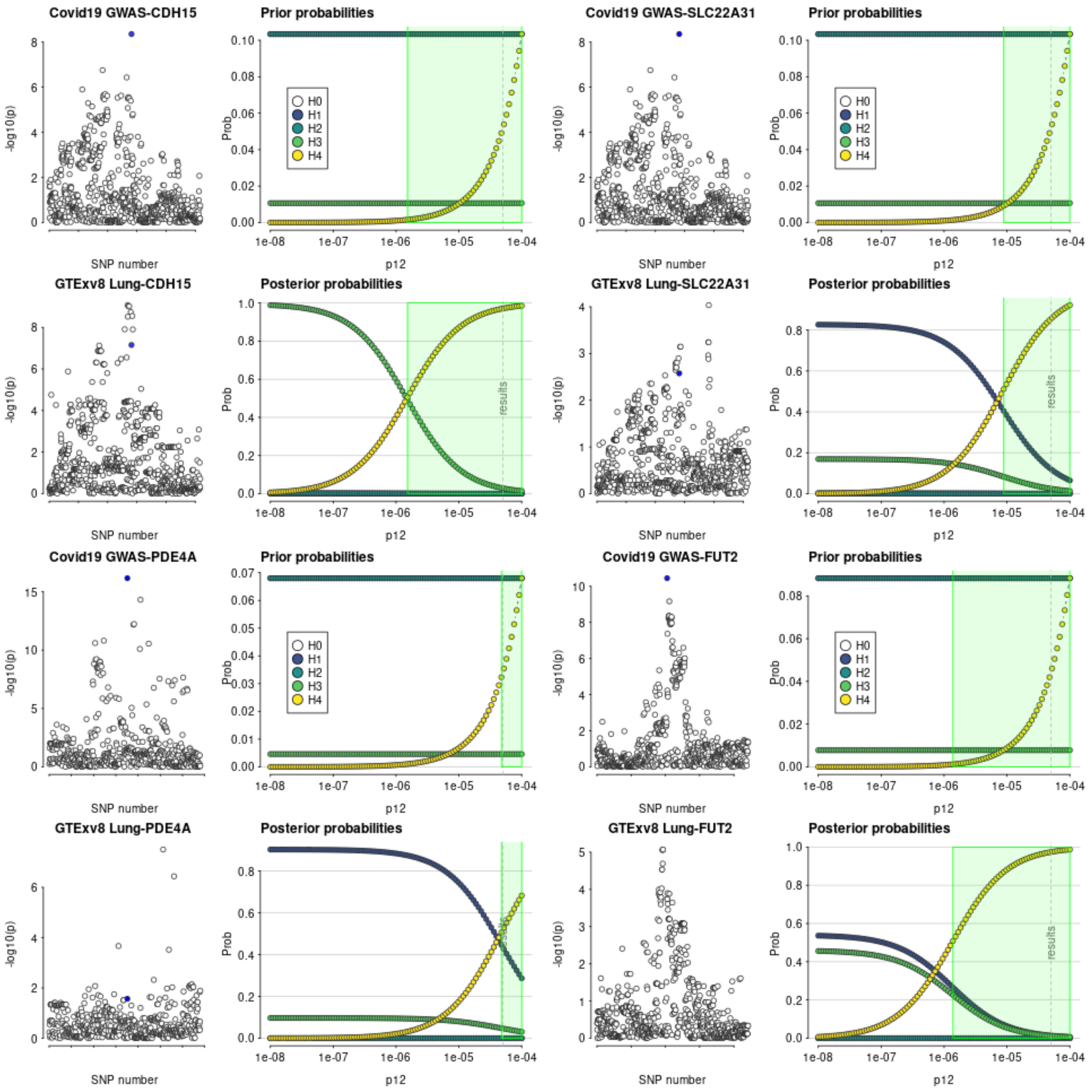
Covid19 GWAS-CDH15
Prior probabilities
Covid19 GWAS-SLC22A31
Prior probabilities
GTExv8 Lung-CDH15
Posterior probabilities
GTExv8 Lung-SLC22A31
Posterior probabilities
Covid19 GWAS-PDE4A
Prior probabilities
Covid19 GWAS-FUT2
Prior probabilities
GTExv8 Lung-PDE4A
Posterior probabilities
GTExv8 Lung-FUT2
Posterior probabilities
-log10(p)
SNP number
Prob
p12
H0
H1
H2
H3
H4
results

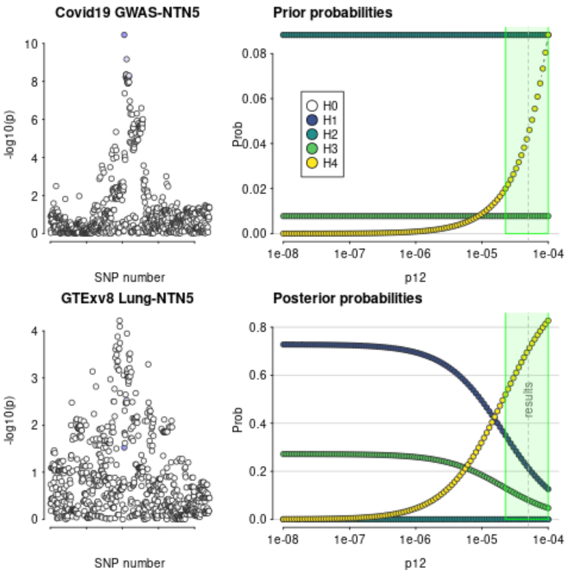

Supplementary Figure 27: Sensitivity analysis for colocalisation of TWAS-significant loci in lung (GTEXv8 data) with GWAS signals. Evidence for the following hypotheses is plotted across a range of prior probabilities: H0 - neither trait has a genetic association in the region; H1 - only trait 1 has a genetic association in the region; H2 - only trait 2 has a genetic association in the region; H3 - both traits are associated, but with different causal variants; H4 - both traits are associated and share a single causal variant.

Colocalisation Summary

chr:pos (hg38)	rsid	REF	ALT	Expression
1:155066988	rs114301457	C	T	-
1:155175305	rs7528026	G	A	-
1:155197995	rs41264915	A	G	*MUC1*
2:60480453	rs1123573	A	G	-
3:45796521	rs2271616	G	T	*SLC6A20, CCR5*
3:45859597	rs73064425	C	T	*LZTFL1, CCR9*
3:146517122	rs343320	G	A	-
5:131995059	rs56162149	C	T	*ACSL6, FNIP1*
6:32623820	rs9271609	T	C	*HLA-DRB1*
6:41515007	rs2496644	A	C	-
9:21206606	rs28368148	C	G	-
11:34482745	rs61882275	G	A	-
12:132489230	rs56106917	GC	G	-
13:112889041	rs9577175	C	T	*ATP11A*
15:93046840	rs4424872	T	A	-
16:89196249	rs117169628	G	A	*SLC22A31, CDH15*
17:46152620	rs2532300	T	C	*ARHGAP27*
17:49863260	rs3848456	C	A	-
19:4717660	rs12610495	A	G	-
19:10305768	rs73510898	G	A	-
19:10352442	rs34536443	G	C	*TYK2, PDE4A*
19:48697960	rs368565	C	T	*FUT2, NTN5, RASIP1*
21:33230000	rs17860115	C	A	-
21:33287378	rs8178521	C	T	-
21:33959662	rs35370143	T	TAC	-

Supplementary Table 12: Lead signals with genes where is evidence of gene expression affecting disease severity, found by TWAS and colocalisation analysis.

Mendelian Randomisation

Exposure	Instruments	$nsnp$	$nsnp_{hgib2.23m}$	Gene
ABO.9253.52.3	rs10793953,rs113395696,rs138490087,rs138683771,rs144539704,rs150258577,rs28632066,rs3094373,rs3124755,rs3124762, rs35775139,rs41297217,rs4489379,rs609202,rs62574561,rs62574656,rs68032997,rs687289,rs7025839,rs71503180,rs72779222,rs8176765	22	20	ABO
C1GALT1C1.5735.54.3	rs10793957,rs11507714,rs12702585,rs176699,rs2519093,rs7787942,rs78712546	7	4	C1GALT1C1
CAMK1.3592.4.3	rs111360116,rs115128381,rs1200143,rs12029101,rs12035622,rs12093387,rs147474835,rs2090515,rs2301518, rs35866785,rs4525,rs57904876,rs6427197,rs72706368,rs72708017,rs72840032	16	17	CAMK1
CCL25.14068.29.3	rs1204494,rs12611310,rs2086149,rs214099,rs3136653,rs35106244	6	5	CCL25
CD209.3029.52.2	rs11791119,rs145526382,rs28632066,rs3094373,rs3124762,rs41296094,rs4804224,rs529565,rs60526253,rs656105,rs735240,rs7868232,rs8176765	13	11	CD209
F8.13499.30.3	rs11930928,rs138683771,rs28632066,rs3094373,rs505922,rs656105	6	5	F8
FAM3D.13102.1.3	rs10901250,rs11671705,rs149189328,rs3094373,rs3124762,rs34271003,rs550057,rs56292712,rs567493,rs601338,rs623179	11	15	FAM3D
GOLM1.8983.7.3	rs10901250,rs149189328,rs3094377,rs550057,rs601338,rs623179	6	5	GOLM1
ICAM1.4342.10.3	rs10407947,rs112407609,rs113337445,rs114170067,rs11666263,rs12974373,rs12983316,rs1801714,rs281421,rs5027494,rs510506, rs5498,rs55925728,rs62131890,rs6511610,rs73009557,rs76317591,rs76923681,rs78439352,rs78503958,rs8113091	21	24	ICAM1
ICAM5.5124.62.3	rs116988145,rs12459144,rs281439,rs45524632,rs635634,rs7249914	6	8	ICAM5
ICAM5.8245.27.3	rs139742916,rs281419,rs281439,rs35193259,rs45524632,rs4804510,rs635634,rs7249914	8	11	ICAM5
IL27RA.5132.71.3	rs1056143,rs35026308,rs62622787,rs7255254,rs74183061,rs8176743	6	7	IL27RA
IL3RA.13744.37.3	rs117164445,rs176699,rs2039184,rs2519093,rs28422057,rs3118667,rs8176693,rs9330460	8	8	IL3RA
PDGFRL.9713.67.3	rs116277633,rs138169253,rs1388604,rs140847751,rs17141074,rs17221605,rs17650274,rs180900701, rs36136468,rs4683234,rs56226331,rs58357264,rs59903532,rs77481436,rs9850642,rs9860011,rs9883208	17	19	PDGFRL
SELE.3470.1.2	rs11507716,rs11603123,rs117164445,rs12288924,rs138704916,rs2039184,rs2519093,rs3124758,rs554710,rs656105,rs739469,rs8176694,rs9330460	13	11	SELE
TLR4.LY96.3647.49.4	rs1252041,rs4986790,rs635634,rs6472812,rs67017252	5	-	TLR4:LY96

Supplementary Table 13: Exposures and instruments for the 16 significant protein in GSMR analysis. nsnps indicates the number of snps used in the analysis in GenOMICC and HGI B2 and 23andme. Gene corresponds to the Gene name for the significant exposure

Aggregate variant testing (AVT)

Aggregate variant testing on aggCOVID_v4.2 was performed using SKAT-O as implemented in SAIGE-GENE v0.44.5[10]. Variant and sample QC for the preparation of the aggregate files has been described elsewhere. In addition, the following filters were applied to the masked aggregate dataset:

- Bi-allelic SNPs only
- Minor allele frequency < 0.005
- Site wide missingness < 0.05
- Differential missingness between cases and controls, mid-p value $< 10^{-5}$

All the variants in the dataset were annotated using VEP v99.

Masks and Model

Two functional annotation masks were applied on top of the filters detailed. The first is a strict putative loss of function (*pLoF*) filter, where only variants that are annotated by Loftee as high confidence loss of function are included. The second is a more lenient filter (*missense*) where all variants from the strict filter are included, together with all variants that have a consequence of missense or worse as annotated by VEP, with a CADD_PHRED score of ≥ 10 (CADD version 1.5). The covariates used in the model were the same as for the single variant analysis: sex, age, age^2, $age * sex$ and 20 (population-specific) principal components generated from common variants (MAF $\geq 5\%$).

The tests were run separately by genetically predicted ancestry, on all protein-coding genes as annotated by Ensembl.

AVT results

Supplementary table 14 shows the number of tested genes per mask per predicted ancestry. These numbers were used to apply a Bonferonni correction on the SKAT-O P-values from SAIGE-GENE on a per population basis. The P-value thresholds for gene-wide significance were taken as $0.05/n * 2$, with n being the number of tested genes in that population, divided by 2 (the number of masks used). This makes the assumption that each gene was tested by both masks, which is conservative for the missense threshold.

**Cohort**	**Tested genes, pLof mask**	**P-value threshold, pLof mask**	**Tested genes, missense mask**	**P-value threshold, missense mask**
EUR	7,352	3.4e-06	18,631	1.3e-06
SAS	1,435	1.7e-05	17,291	1.4e-06
AFR	763	3.3e-05	16,125	1.6e-06
EAS	265	9.4e-05	12,519	2.0e-06

Supplementary Table 14: Number of tested genes per mask per predicted ancestry, with Bonferroni-corrected P-values used to assess gene-wide significance.

No significant associations were found across any of the populations. Supplementary figure 28 shows the Manhattan and Q-Q plots for each predicted ancestry using the *pLoF* mask, and supplementary

figure 29 shows the Manhattan and Q-Q plots for each predicted ancestry using the *missense* mask. Supplementary File AVTsuppinfo.xlsx, sheet A and sheet B, show the top ten genes by P-value for each predicted ancestry and all combined ancestries, respectively.

Supplementary File AVTsuppinfo.xlsx, sheet C, shows the top genes that were highlighted as part of the GWAS analysis, ranked by p value per predicted ancestry. Supplementary File AVTsuppinfo.xlsx, sheet D shows the SKAT-O P-values for the 13 genes involved in the regulation of type I and III interferon immunity that were implicated in severe Covid-19 pneumonia[11], ranked by p value per predicted ancestry.

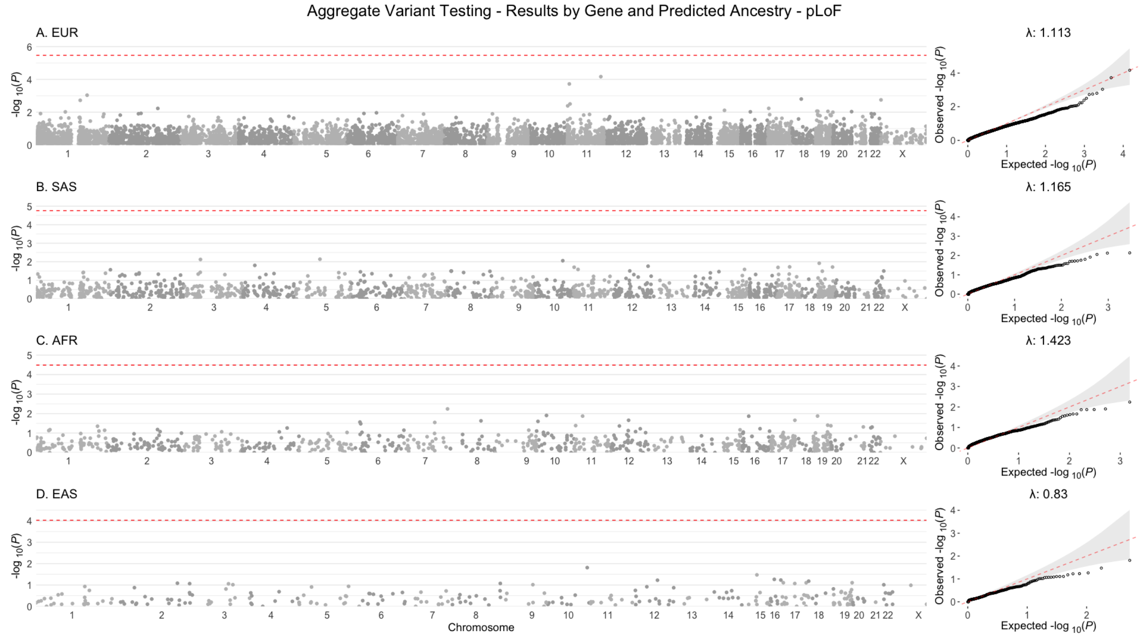

Supplementary Figure 28: Gene-level manhattan and Q-Q plots per predicted ancestry for the *pLof* mask. Each point in the manhattan plot represents a gene. Panels from top to bottom are for EUR (A), SAS (B), AFR (C) and EAS (D). The red dashed lines indicate Bonferonni corrected "gene-wide" P-values (see Supplementary Table 14).

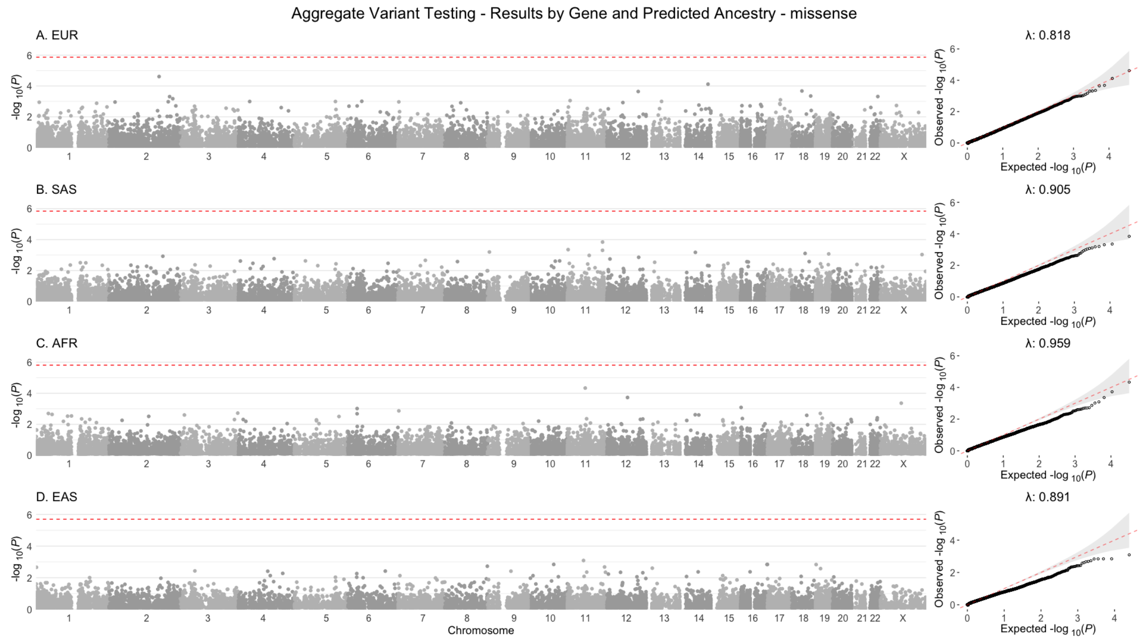

Supplementary Figure 29: Gene-level manhattan and Q-Q plots per predicted ancestry for the *missense* mask. Each point in the manhattan plot represents a gene. Panels from top to bottom are for EUR (A), SAS (B), AFR (C) and EAS (D). The red dashed lines indicate Bonferonni corrected "gene-wide" P-values (see Supplementary Table 14).

HLA Inference and Association Tests

HLA imputation using HIBAG

HLA types were imputed at two field (4-digit) resolution for all samples within aggV2 and aggCOVID_v4.2 for the following seven loci: HLA-A, HLA-C, HLA-B, HLA-DRB1, HLA-DQA1, HLA-DQB1, and HLA-DPB1 using the HIBAG package in R[12]. We used ancestry specific pre-fit classifiers trained on the Illumina 1M Duo genotyping array on individuals of either European, Asian, and African ancestry dependent on the assigned ancestry of the sample in hand. The list of HLA alleles represented in the reference panel is shown in Supplementary File HLAsuppinfo.xlsx (Sheet A). HIBAG requires genotyped data in PLINK format as input. We lifted over the GRCh38 variant calls from aggV2 and aggCOVID_v4.2 for the extended (xMHC) region to hg19, keeping the variants included in the pre-trained classifiers for the seven HLA loci which were present in both the aggV2 and aggCOVID_v4.2 call-sets to ensure that the variants used for the imputation were the same across the two datasets. We applied a threshold of T=0.5 on the posterior probabilities returned by HIBAG, as in the original publication.

HLA inference using HLA*LA and concordance between HIBAG and HLA*LA callsets

We used a second HLA inference method, HLA*LA[13], to assess concordance and ensure call rates were comparable between the two methods. HLA*LA (version fe00f82) was used with GRCh38 IMGT population reference graphs to infer classical HLA types at G-group resolution for the three class I genes (HLA-A, HLA-C, HLA-B) and four class II genes (HLA-DRB1, HLA-DQA1, HLA-DQB1, HLA-DPB1) that were also umputed with HIBAG. HLA*LA implements a graph alignment model for HLA type inference, based on the projection of linear alignments onto a variation graph. Whole-genome sequencing BAM/CRAM files including unmapped reads were used as input. Where CRAM files were used (alignments from aggCOVID_v4.2 cohort), the reference genome FASTA file used for the original alignment was also provided.

Note that at time of writing, only 82% of aggV2 and aggCOVID_v4.2 samples had their HLA types inferred by HLA*LA (Supplementary File HLAsuppinfo.xlsx (Sheet B)). All samples had their HLA types imputed using HIBAG.

For samples for which we had both HIBAG and HLA*LA calls (n=45,796), we compared the 4-digit resolution alleles called from HIBAG with the alleles called from HLA*LA. As HLA*LA calls alleles at G-group resolution, we took all 4-digit alleles belonging to each G-Group and compared these to the HIBAG calls. For example, if a sample is called as A*01:01:01G, the mapped HLA alleles at 4-digit resolution within HLA*LA are 01:01, 01:04, 01:10, 01:13, 01:14, 01:15, 01:22, 01:32, 01:37, 01:45, 01:56, 01:81, 01:87. These were compared against the HIBAG 4-digit calls. If the 4-digits matched exactly (in either diploid combination - i.e. 01:01 / 01:04 vs 01:04 / 01:01), then sample alleles were deemed concordant. We found that >96% of calls were identical between HIBAG and HLA*LA.

The percentage of concordant calls between HIBAG and HLA*LA by ancestry and locus is shown in Supplementary File HLAsuppinfo.xlsx (Sheet C).

HLA Association Tests

HLA calls from HIBAG were aggregated into a single multi-sample VCF file containing sample genotypes for all observed HLA calls. Per sample, the genotypes of any allele call with posterior probability < 0.5 were set to missing. If a sample then had a missing genotype for a particular allele, all other alleles at that locus were also set to missing. For each locus, samples that did not harbour a specific HLA allele (either in a heterozygous or homozygous alternate state), were set to homozygous reference; unless already set to missing from the above mask.

HLA association analysis (single variant association tests) was run under an additive model using SAIGE (logistic mixed-model regression) version 0.44.5; in an identical fashion to the SNV GWAS. The multi-sample VCF of aggregated HLA type calls from HIBAG were used as input. The set of 60K high-quality common SNPs aggCOVID_v4.2_aggV2_HQSNPs were used to create the GRM and variance ratio files. HLA association tests were run per ancestry (EUR, SAS, EAS, AFR) on unrelated individuals for the sev_vs_mld_aggV2 cohort. Each HLA association test was run using: sex, age, age^2, $age \times sex$, and the first 20 ancestral principle components as covariates. No minimum minor allele count / frequency threshold was set. Results can be seen in Supplementary File HLAsuppinfo.xlsx (Sheet D). Note this table combines the results for all ancestries (EUR, SAS, EAS, and AFR) - which is referenced in the first column of the table. The table is sorted by ancestry and alphabetically by allele.

HLA-DRB1*:04:01 was the only genome-wide significant HLA allele ($OR = 0.80, 95\%CI = 0.75 - 0.86, P = 1.6 \times 10^{-10}$, in EUR), having a protective effect (casesMAF: 9.6%, controlsMAF: 11.7%). In EUR, the DRB1*04:01 allele had a low rate of missingness (call rate >0.92 at T=0.5 posterior probability threshold), and was in Hardy-Weinberg equilibrium for both cases (p=0.32) and controls (p=0.17). The observed allele frequency for HLA-DRB1*04:01 was 0.1%, 0.08%, and 0.02% for AFR, EAS, and SAS cohorts respectively. A meta-analysis was performed using METAL with an inverse-variance weighted method across the four populations. DRB1*04:01 remained the only significant association ($OR = 0.80, 95\%CI = 0.75 - 0.86, P = 1.4 \times 10^{-10}$), Supplementary File HLAsuppinfo.xlsx (Sheet E).

We also ran our association analysis on EUR samples with concordant calls between HIBAG and HLA*LA using the same model as for the full analysis, and confirmed the observed association with HLA-DRB1*04:01 ($OR = 0.78, 95\%CIs : 0.75 - 0.81, P = 1.3 \times 10^{-11}$) which had a lower $P - value$ than for the lead variant ($OR = 0.88, 95\%CIs : 0.86 - 0.90, P = 4.4 \times 10^{-9}$), consistent with our results on the full HIBAG callset.

We also examined the robustness of our results to the choice of call threshold for the posterior probability for HIBAG and performed the association tests for EUR without any call threshold (CT=0, i.e. best guess), with CT=0.5, and with CT=0.7. We found that DRB1*04:01 remained the only significant locus across each of the three CT values tested with the Odds Ratios and P-values being extremely consistent across all alleles (Supplementary Figure 30).

HLA conditional Analysis

We conducted a conditional analysis, controlling for the HLA-DRB1*04:01 allele, on the main GWAS results within the extended MHC region. This analysis was performed on the European sev_vs_mld_aggV2 cohort. To do this, we firstly regressed out the effect of DRB1*04:01 (including age, sex, $age \times sex$, age^2, and the first 20 population PCs), and performed linear regression on

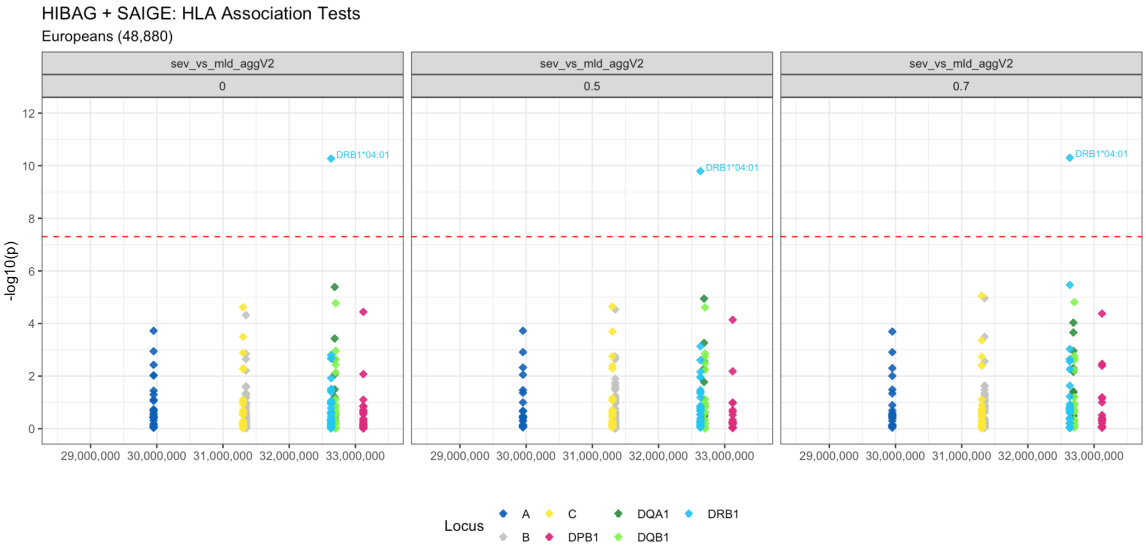

Supplementary Figure 30: Robustness of HLA association results to different posterior probability call thresholds for HIBAG.Manhattan plot of HLA allele associations across the extended MHC region with Covid-19 critical illness for the EUR cohort. Each panel corresponds to association results obtained using genotypes that were called using a different call threshold for HIBAG (0, 0.5 and 0.7, respectively). Diamonds represent the HLA each allele association, coloured by locus. The lead variant from the lead HLA allele is labelled. The dashed red line is the Bonferroni-corrected genome-wide significance threshold for Europeans.

the residuals using the GWAS variant genotypes as the dependent variable. No variants within the extended MHC remained genome-wide significant upon conditioning on DRB1*04:01. The top GWAS signal (chr6:32623820 T/C; $OR = 0.88, 95\%CI = 0.84 - 0.92, P = 3.3 \times 10^{-9}$) was attenuated following conditional analysis ($P = 0.001$). Extended data Figure 6 shows the results of the combined HLA and GWAS association results and the conditional analysis.

Enrichment analysis

Enrichment analysis was run with XGR algorithm[14] using 19 genes identified by TWAS and colocalisation analysis (Figure 2) and genes with missense mutations that were either lead variants (Table 1) or were part of the credible sets identified by the fine-mapping analysis with SusieR (Extended Data Table 1). The combined input gene list was: *MUC1*, *SLC6A20*, *CCR9*, *LZTFL1*, *CCR5*, *ACSL6*, *FNIP1*, *ATP11A*, *CDH15*, *SLC22A31*, *CDH15*, *IFNAR2*, *DPP9*, *IL10RB*, *TYK2*, *NTN5*, *FUT2*, *PDE4A*, *THBS3*, *PLSCR1*, *CSF2*, *IFNA10*.

Enriched Ontology	Term Name	Z-score	P-value	FDR	Genes
GO Biological Component	cytokine-mediated signaling pathway	12.2	2.8E-10	1.4E-09	*CCR5, CSF2, IFNA10, IFNAR2, IL10RB, MUC1, TYK2*
Reactome pathways	Regulation of IFNA signaling	14	6.8E-08	3.4E-07	*IFNA10, IFNAR2, TYK2*
KEGG pathways	Jak-STAT signaling pathway	7.93	4.8E-07	7.6E-07	*CSF2, IFNA10, IFNAR2, IL10RB, TYK2*
KEGG pathways	Cytokine-cytokine receptor interaction	7.1	5E-07	7.6E-07	*CCR5, CCR9, CSF2, IFNA10, IFNAR2, IL10RB*
GO Biological Component	type I interferon signaling pathway	10.9	7.3E-07	1.8E-06	*IFNA10, IFNAR2, TYK2*
GO Biological Component	defense response to virus	8.32	1.7E-06	2.8E-06	*IFNA10, IFNAR2, IL10RB, PLSCR1*
Reactome pathways	Interferon alpha/beta signaling	8.4	3.9E-06	9.7E-06	*IFNA10, IFNAR2, TYK2*
Reactome pathways	Cytokine Signaling in Immune system	5.08	6.8E-05	0.00011	*CSF2, IFNA10, IFNAR2, TYK2*
GO Biological Component	immune response	5.27	7.5E-05	9.4E-05	*CCR5, CCR9, CSF2, IL10RB*
Reactome pathways	Interferon Signaling	5.06	0.00014	0.00017	*IFNA10, IFNAR2, TYK2*
KEGG pathways	Natural killer cell mediated cytotoxicity	4.87	0.00019	0.00019	*CSF2, IFNA10, IFNAR2*
GO Cellular Component	integral component of plasma membrane	4.03	0.00024	0.0017	*CCR5, CCR9, IFNAR2, MUC1, PLSCR1, SLC6A20*
GO Cellular Component	cell surface	3.12	0.0027	0.0093	*CCR5, CCR9, CDH15*
GO Cellular Component	perinuclear region of cytoplasm	2.82	0.0043	0.01	*PDE4A, PLSCR1, THBS3*
GO Cellular Component	extracellular region	2.33	0.0089	0.016	*CSF2, IFNA10, IFNAR2, NTN5, THBS3*
GO Molecular Function	calcium ion binding	2.31	0.01	0.021	*CDH15, PLSCR1, THBS3*
GO Cellular Component	Golgi apparatus	2.09	0.015	0.02	*CDH15, FUT2, PLSCR1*
Reactome pathways	Immune System	1.92	0.018	0.018	*CSF2, IFNA10, IFNAR2, TYK2*
GO Biological Component	G protein-coupled receptor signaling pathway	1.68	0.03	0.03	*CCR5, CCR9, PDE4A*

Supplementary Table 15: Enrichment analysis was applied to 19 genes identified by TWAS and colocalisation analysis and/or harbouring missense mutations. These genes were input into the XGR algorithm[14] to look for enrichment in Gene Ontology (GO) terms (Biological component, Cellular component and Molecular function) and curated KEGG and Reactome pathways. The table shows all enrichment terms with a false-discovery rate (FDR)<0.05. The most significant enrichment was in the cytokine-mediated, interferon and Jak-STAT signalling pathways.

Meta-analysis by information content (MAIC)

In order to put the results in the context of existing knowledge of host genes implicated in SARS-CoV-2 replication or pathophysiology of Covid-19, we use meta-analyis by information content (MAIC)[15] to incorporate lists of named genes from a large systematic review of in vitro and in vivo studies.[16] Remarkably, the top 2000 named genes in our metaTWAS contributes 19.5% of the total information content in this composite analysis (Supplementary Figure 31). Full results are available at baillielab.net/maic/covid.

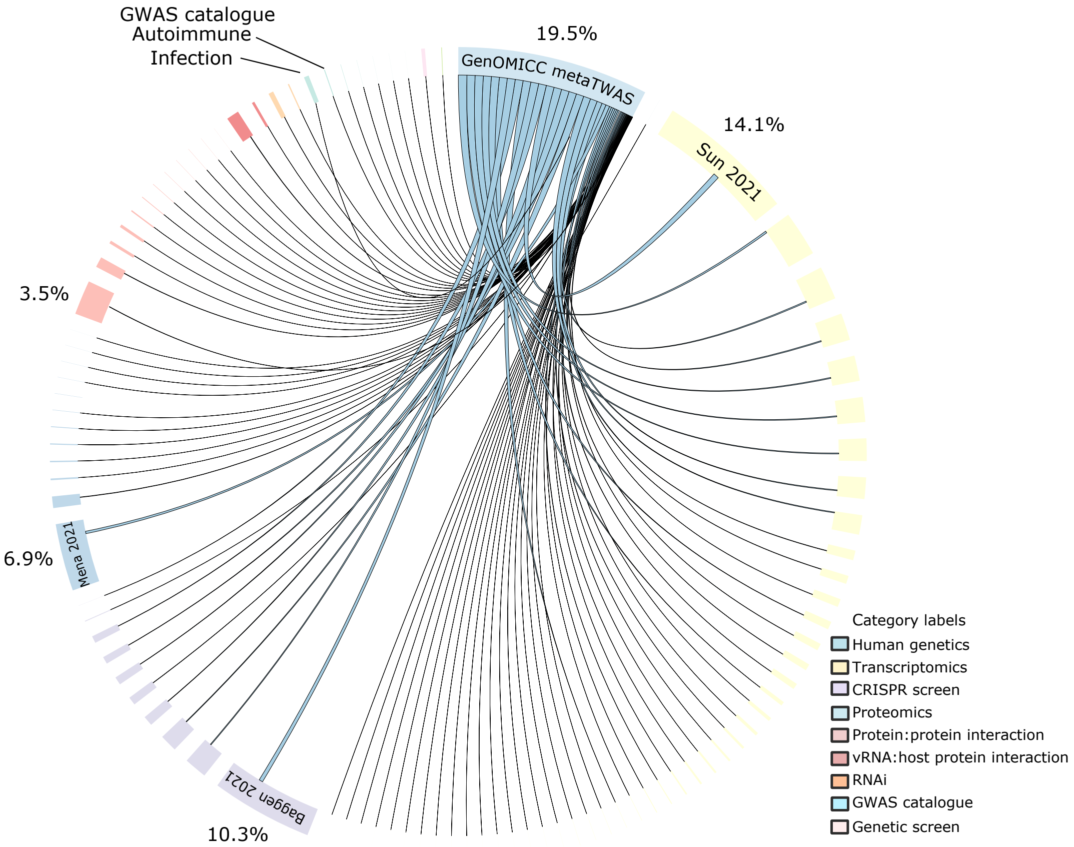

Supplementary Figure 31: Circular diagram of shared information content among data sources using MAIC analysis. Each data source is represented by a coloured block on the outer ring of the circle; the size of data source blocks is proportional to the summed information content of the input list—that is, the total contribution that this data source makes to the aggregate, calculated as the sum of the MAIC gene scores contributed by that list and represented numerically for datasets with the highest information content. Lines are coloured according to the dominant data source. Data sources within the same category share the same colour (legend). The largest categories and data sources are labelled. An interactive version of this figure is available at baillielab.net/maic/covid.

References

[1] Pairo-Castineira, E. *et al.* Genetic mechanisms of critical illness in Covid-19. *Nature* 1–1 (2020).

[2] Wu, Y., Zheng, Z., Visscher, P. M. & Yang, J. Quantifying the mapping precision of genome-wide association studies using whole-genome sequencing data. *Genome Biology* **18**, 86 (2017).

[3] Consortium, T. G. The GTEx Consortium atlas of genetic regulatory effects across human tissues. *Science* **369**, 1318–1330 (2020). URL https://science.sciencemag.org/content/369/6509/1318. Publisher: American Association for the Advancement of Science _eprint: https://science.sciencemag.org/content/369/6509/1318.full.pdf.

[4] Ellinghaus, D. *et al.* Genomewide association study of severe covid-19 with respiratory failure. *The New England journal of medicine* **383**, 1522–1534 (2020).

[5] The COVID-19 Host Genetics Initiative & Ganna, A. Mapping the human genetic architecture of COVID-19 by worldwide meta-analysis. *medRxiv* 2021.03.10.21252820 (2021).

[6] Degenhardt, F. *et al.* New susceptibility loci for severe COVID-19 by detailed GWAS analysis in European populations. *medRxiv* 2021.07.21.21260624 (2021).

[7] Pairo-Castineira, E. *et al.* Genetic mechanisms of critical illness in COVID-19. *Nature* **591**, 92–98 (2021).

[8] Ning, Z., Pawitan, Y. & Shen, X. High-definition likelihood inference of genetic correlations across human complex traits. *Nature Genetics* **52**, 859–864 (2020).

[9] Bulik-Sullivan, B. K. *et al.* LD Score regression distinguishes confounding from polygenicity in genome-wide association studies. *Nature Genetics* **47**, 291–295 (2015).

[10] Zhou, W. *et al.* Scalable generalized linear mixed model for region-based association tests in large biobanks and cohorts. *Nature Genetics* **52**, 634–639 (2020). URL https://www.nature.com/articles/s41588-020-0621-6.

[11] Zhang, Q. *et al.* Inborn errors of type I IFN immunity in patients with life-threatening COVID-19. *Science (New York, N.y.)* **370**, eabd4570 (2020). URL https://www.ncbi.nlm.nih.gov/pmc/articles/PMC7857407/.

[12] Zheng, X. *et al.* HIBAG - HLA genotype imputation with attribute bagging. *Pharmacogenomics Journal* **14**, 192–200 (2014).

[13] Dilthey, A. T. *et al.* HLA*LA—HLA typing from linearly projected graph alignments. *Bioinformatics* **35**, 4394–4396 (2019). URL https://doi.org/10.1093/bioinformatics/btz235. https://academic.oup.com/bioinformatics/article-pdf/35/21/4394/30330845/btz235.pdf.

[14] Fang, H., Knezevic, B., Burnham, K. L. & Knight, J. C. XGR software for enhanced interpretation of genomic summary data, illustrated by application to immunological traits. *Genome Medicine* **8**, 129 (2016). URL https://www.ncbi.nlm.nih.gov/pmc/articles/PMC5154134/.

[15] Li, B. *et al.* Genome-wide CRISPR screen identifies host dependency factors for influenza A virus infection. *Nature Communications* **11**, 164 (2020).

[16] Parkinson, N. *et al.* Dynamic data-driven meta-analysis for prioritisation of host genes implicated in COVID-19. *Scientific Reports* **10**, 22303 (2020).
