# Supplementary material for: Whole-genome sequencing reveals host factors underlying critical COVID-19
Source: Nature. 2022 Mar 7;607(7917):97–103. doi: 10.1038/s41586-022-04576-6 (PMC9259496; doi:10.1038/s41586-022-04576-6)
Supplement: Supplementary file 2 — Reporting Summary [file 41586_2022_4576_MOESM2_ESM.pdf]

nature portfolio

Corresponding author(s): J. Kenneth Baillie

Last updated by author(s): Jan 2, 2022

Reporting Summary

Nature Portfolio wishes to improve the reproducibility of the work that we publish. This form provides structure for consistency and transparency in reporting. For further information on Nature Portfolio policies, see our Editorial Policies and the Editorial Policy Checklist.

Statistics

For all statistical analyses, confirm that the following items are present in the figure legend, table legend, main text, or Methods section.

n/a	Confirmed	
☐	☒	The exact sample size (*n*) for each experimental group/condition, given as a discrete number and unit of measurement
☐	☒	A statement on whether measurements were taken from distinct samples or whether the same sample was measured repeatedly
☐	☒	The statistical test(s) used AND whether they are one- or two-sided<br>*Only common tests should be described solely by name; describe more complex techniques in the Methods section.*
☐	☒	A description of all covariates tested
☐	☒	A description of any assumptions or corrections, such as tests of normality and adjustment for multiple comparisons
☐	☒	A full description of the statistical parameters including central tendency (e.g. means) or other basic estimates (e.g. regression coefficient) AND variation (e.g. standard deviation) or associated estimates of uncertainty (e.g. confidence intervals)
☐	☒	For null hypothesis testing, the test statistic (e.g. *F*, *t*, *r*) with confidence intervals, effect sizes, degrees of freedom and *P* value noted<br>*Give P values as exact values whenever suitable.*
☐	☒	For Bayesian analysis, information on the choice of priors and Markov chain Monte Carlo settings
☒	☐	For hierarchical and complex designs, identification of the appropriate level for tests and full reporting of outcomes
☐	☒	Estimates of effect sizes (e.g. Cohen's *d*, Pearson's *r*), indicating how they were calculated

Our web collection on statistics for biologists contains articles on many of the points above.

Software and code

Policy information about availability of computer code

Data collection	Nucleon Kit (Cytiva), Chemagic 360 platform using Chemagic DNA blood kit (Perkin Elmer) . T Qubit and normalised Illumina TruSeq DNA PCR-Free High Throughput Sample Preparation kit, Illumina Hiseq X instrument (for 100,000 Genomes Project samples), NovaSeq instrument (for the COVID-19 critical and mild cohorts).
Data analysis	VerifyBAMID, DRAGEN(v3.2.22), Illumina North Star Version 4 Whole Genome Sequencing Workflow (NSV4, version 2.6.53.23), iSAAC Aligner (version 03.16.02.19), Starling Small Variant Caller (version 2.4.7), GVGCFGenotyper (GG) v3.8.1, vt v0.57721, gvcfgenotyper v2019.02.26, bcftools v1.10.2, plink 1.9, HiSeq+NSV4, HiSeq+Pipeline 2.0, NovaSeq+Pipeline 2.0, KING 2.1, plink2, GCTA v1.93.1_beta, Strelka2, SAIGE v0.44.5, GTCA 1.9.3, SusieR v0.11.42, VEPv104, metal 2018-08-28, MetaSubtract package (v1.60), R(v4.0.2), SAIGE-GENE v0.44.5, LOFTEE, VEPv99, MetaXCan (v0.6.5), coloc R package 5.1.0, R 3.6.3, GSMR, Heidi, HIBAG R package 1.8.3, XGR package (20-Apr-2020), LDSC (v1.0.1), HDL(v.1.4.0), REGENIEv2.2

For manuscripts utilizing custom algorithms or software that are central to the research but not yet described in published literature, software must be made available to editors and reviewers. We strongly encourage code deposition in a community repository (e.g. GitHub). See the Nature Portfolio guidelines for submitting code & software for further information.

Data

Policy information about availability of data

All manuscripts must include a data availability statement. This statement should provide the following information, where applicable:

- Accession codes, unique identifiers, or web links for publicly available datasets
- A description of any restrictions on data availability
- For clinical datasets or third party data, please ensure that the statement adheres to our policy

Full summary data in support of the findings of this study will available for download from https://genomicc.org/data concurrently with publication. Individual-level data can be analysed by qualified researchers in the UK Outbreak Data Analysis Platform at the University of Edinburgh by application at https://genomicc.org/data. Genomicc data for 1000,000 genomes participants and cases are available through the Genomics England research environment.
The full GWAS summary statistics for the 23andMe discovery data set will be made available through 23andMe to qualified researchers under an agreement with 23andMe that protects the privacy of the 23andMe participants. Please visit https://research.23andMe.com/dataset-access/ for more information and to apply to access the data.

Field-specific reporting

Please select the one below that is the best fit for your research. If you are not sure, read the appropriate sections before making your selection.

☒ Life sciences ☐ Behavioural & social sciences ☐ Ecological, evolutionary & environmental sciences

For a reference copy of the document with all sections, see nature.com/documents/nr-reporting-summary-flat.pdf

Life sciences study design

All studies must disclose on these points even when the disclosure is negative.

Sample size	Cases: n=7,491, controls n=48,400 (mild cases n=1630, 100k controls n=46,770).<br>European ancestry ncases=5,989 ncontrols=41,891<br>South Asian ancestry ncases=788, ncontrols=3,793<br>African ancestry ncases=440, ncontrols=1,350<br>East Asian ancestry ncases=274, ncontrols=366
Data exclusions	no exclusions
Replication	Data from the Host Genetics Initiative (HGI) data freeze 6 B2 analysis (hospitalised cases) were combined in a meta-analysis with data shared by 23andMe.Inc . We removed signals in HGI derived from GenOMICC cases for independence. 22 of the 25 independent GWAS signals were replicated.<br>Replication of rs28368148 and rs4424872 was attempted using a trans-ancestry meta-analysis UKB, AncestryDNA, Penn Medicine Biobank (PMBB), andGeisinger Health Systems (GHS) totaling 9937 hospitalized COVID-19 casesand 1,059,390 controls (COVID-19 negative or unknown).<br>1 extra loci (rs28368148) was replicated using this method. The loci not replicated correspond to the lead snps: rs9271609 in HLA region and rs4424872 next to RGMA.
Randomization	Not relevant to the study. There wasn't any allocation to experimental groups
Blinding	Not relevant to the study.

Reporting for specific materials, systems and methods

We require information from authors about some types of materials, experimental systems and methods used in many studies. Here, indicate whether each material, system or method listed is relevant to your study. If you are not sure if a list item applies to your research, read the appropriate section before selecting a response.

Materials & experimental systems

n/a	Involved in the study
☒	☐ Antibodies
☒	☐ Eukaryotic cell lines
☒	☐ Palaeontology and archaeology
☒	☐ Animals and other organisms
☐	☒ Human research participants
☒	☐ Clinical data
☒	☐ Dual use research of concern

Methods

n/a	Involved in the study
☒	☐ ChIP-seq
☒	☐ Flow cytometry
☒	☐ MRI-based neuroimaging

Human research participants

Policy information about studies involving human research participants

Population characteristics

Severe COVID-19 (n=7491). Significant comorbidities: 1,605. Died(60 days) 2154, Invasive Ventilation 4028.
mean age=60, mean BMI=29.9
European ancestry (n=5,989): males 4,062 females 1,927.
South Asian ancestry (n=788): males:586, females:202
East Asian ancestry(n=274) males:162, females:112
African ancestry(n=440) males:286, females 154
100K controls: 18,915 unaffected family members of rare diseases participants. 14,701 affected rare diseases participants, 1,005 not assessed for disease status, 12,149 cancer participants.
mean age: 51
mean BMI: 26.1
European ancestry (n=41,384): males 18,971females 22,413
South Asian ancestry (n=3698): males:1802, females:1896
East Asian ancestry(n=352) males:138, females:224
African ancestry(n=1,236) males:632, females:704
Mild COVID-19 cohort. mean age: 46.
European ancestry (n=1507): males:410, females:1,097
South Asian ancestry (n=95): males:43, females:52
East Asian ancestry(n=14) males:8, females:6
African ancestry(n=14) males:6, females:8

Recruitment

Critically ill patients recruited to the GenOMICC study (genomicc.org) had confirmed COVID-19 according to local clinical testing and were deemed, in the view of the treating clinician, to require continuous cardiorespiratory monitoring. In UK practice this kind of monitoring is undertaken in high dependency or intensive care units. Patients were recruited from 224 ICU across the UK.
Participants were recruited to the mild COVID-19 cohort on the basis of having experienced mild (non-hospitalised) or asymptomatic COVID-19. Participants volunteered to take part in the study via a microsite and were required to self-report the details of a positive COVID-19 test. Volunteers were prioritised for genome sequencing based on demographic matching with the critical COVID-19 cohort considering self-reported ancestry, sex, age and location within the UK.
Participants were enrolled in the 100,000 Genomes Project from families with a broad range of rare diseases, cancers and infection by 13 regional NHS Genomic Medicine Centres across England and in Northern Ireland, Scotland and Wales. For this analysis, participants for whom a positive SARS-CoV-2 test had been recorded as of March, 2021 were not included due to uncertainty in the severity of COVID-19 symptoms. Only participants for whom genome sequencing was performed from blood derived DNA were included and participants with haematological malignancies were excluded to avoid potential tumour contamination.

Ethics oversight

Research ethics committees (Scotland 15/SS/0110, England, Wales and Northern Ireland: 19/WM/0247. Current and previous versions of the study protocol are available at genomicc.org/protocol.
UKBiobank Study: ethical approval for the UK Biobank was previouslyobtained from the North West Centre for Research Ethics Committee(11/NW/0382). The work described herein was approved by UK Biobank under application number 26041.
GHS study: approval for DiscovEHR analyseswas provided by the Geisinger Health System Institutional Review Board under project number 2006-0258.
AncestryDNA study: all data for this research project was from subjects who provided prior informed consent to participate in AncestryDNA's Human Diversity Project, as reviewed and approved by our external institutional review board, Advarra (formerlyQuorum). All data was de-identified prior to use.
PMBBstudy: appropriate consent was obtained from each participant regarding storage of biological specimens, genetic sequencing and genotyping, andaccess to all available EHR data. This study was approved by the Institutional Review Board of the University of Pennsylvania and complied with the principles set out in the Declaration of Helsinki. Informed consent was obtained for all study participants.

Note that full information on the approval of the study protocol must also be provided in the manuscript.
